# Tetrabutylammonium Chlorite as an Efficient Oxidant for Controlled Oxidation of Sulfides to Sulfoxides

**DOI:** 10.1002/chem.202404279

**Published:** 2025-02-18

**Authors:** Yuki Itabashi, Shuto Ogata, Yusuke Shimada, Minato Kondo, Nagatoshi Nishiwaki, Tsuyoshi Inoue, Haruyasu Asahara, Kei Ohkubo

**Affiliations:** ^1^ Institute for Open and Transdisciplinary Research Initiatives (OTRI) Osaka University 1–6 Yamada-oka, Suita Osaka 565–0871 Japan; ^2^ Graduate School of Pharmaceutical Sciences Osaka University 1–6 Yamada-oka, Suita Osaka 565–0871 Japan; ^3^ School of Engineering Science Kochi University of Technology Tosayamada, Kami, Kochi 782–8502 Japan

**Keywords:** Chlorine dioxide, Oxygenation, Surface hydrophilization, Quaternary ammonium cation, Radical

## Abstract

Considering the demand for organosulfur materials and the challenges associated with currently used oxidation processes, in this study, we evaluated the counter‐cation of sodium chlorite (Na^+^ClO_2_^−^) with tetrabutylammonium chloride (Bu_4_N^+^Cl^−^) to synthesise tetrabutylammonium chlorite (Bu_4_N^+^ClO_2_^−^). Bu_4_N^+^ClO_2_^−^ exhibited good solubility in organic solvents like chloroform (5.2 mol L^−1^) and ethyl acetate (2.8 mol L^−1^). The oxygenation reaction of organosulfides with Bu_4_N^+^ClO_2_^−^ was examined in an HCl‐acidic ethyl acetate solution under ambient conditions, selectively forming the corresponding sulfoxides as mono‐oxygenated products. The reaction was initiated by the formation of chlorine dioxide (ClO_2_⋅) as an oxidant and an active species generated from Bu_4_N^+^ClO_2_^−^ with HCl. This oxygenation method also enables the surface oxidation of sulfide polymers like polyphenylene sulfide (PPS) resins to increase their surface hydrophilicity. Therefore, the use of Bu_4_N^+^ClO_2_^−^ for applications in organic solvents is an efficient, and highly selective approach for selective oxidation.

## Graphical abstract

Tetrabutylammonium chlorite exhibited excellent solubility in organic solvents and enabled selective oxidation of organosulfides and sulfide polymers under ambient conditions, enhancing surface hydrophilicity effectively.

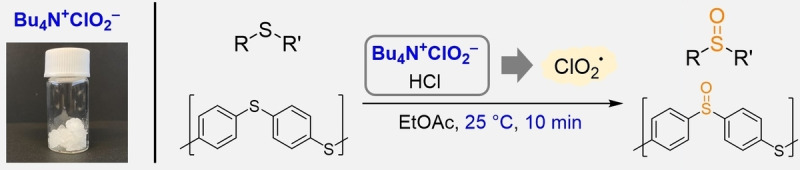

## Introduction

The sulfur atoms in organosulfur compounds can exhibit various oxidation states, providing a means to finely tune the physical and chemical properties of the molecules. Owing to this versatility, these compounds find application in a wide range of products, including natural products, pharmaceuticals, polymer resins, and organic electronic materials.[^1^, ^2^, ^3^, ^4^] Despite their importance, the selective synthesis of organosulfur compounds with specific oxidation states of sulfur presents considerable challenges.[^5^, ^6^, ^7^, ^8^] In the oxidation of sulfides, precise reaction control is critical because unreacted starting materials can remain or excessive oxidation can occur, leading to the formation of sulfones. This necessitates strict temperature control at low temperatures,^[9]^ the use of excess reagents,^[10]^ and extensive purification processes owing to the formation of multiple products. In addition, traditional oxidation methods often require toxic reagents like heavy metals[^11^, ^12^, ^13^] and produce over‐oxidised sulfone and byproducts.^[14]^ Therefore, there is a need to develop selective and efficient oxidation processes.

Sodium chlorite (Na^+^ClO_2_^−^), an easily accessible compound produced by the electrolysis of sodium chloride (NaCl), is known for its potent oxidising properties when combined with hydrochloric acid (HCl) to generate chlorine dioxide (ClO_2_⋅).^[15]^ It reverts to chloride ions (Cl^−^) after oxidation,[^16^, ^17^, ^18^] making it an environment‐friendly choice.^[19]^ However, the application of Na^+^ClO_2_^−^ in organic synthesis is limited due to its poor solubility in organic solvents, requiring the use of aqueous or mixed solvent systems. Organic cations like tetrabutylammonium (Bu_4_N^+^) have been shown to improve the solubility of chlorite salts, enabling more efficient and selective oxidation reactions in various organic solvents.

In this paper, we report an approach in which the counter‐cation of chlorite (ClO_2_^−^) is exchanged from the sodium cation (Na^+^) to Bu_4_N^+^, thereby facilitating its use in organic solvents. This modification enhances the solubility of ClO_2_^−^ in organic media and allows for precise control over its reactivity. Our findings demonstrate the successful selective oxidation of sulfides to sulfoxides under mild conditions. The use of tetrabutylammonium chlorite (Bu_4_N^+^ClO_2_^−^) for organic solvent applications addresses the solubility limitations associated with Na^+^ClO_2_^−^. This approach opens new avenues for selective oxidation of organosulfur compounds under milder conditions.

## Results and Discussion

### Synthesis of Bu_4_N^+^ClO_2_^−^

**Scheme 1:**
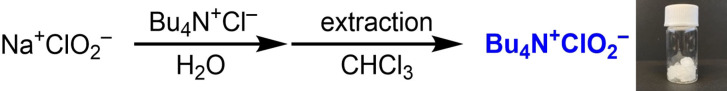
Countercation exchange from Na^+^ClO_2_^−^ to Bu_4_N^+^ClO_2_^−^ which obtained as a white solid in the bottle.

Because of the extremely low solubility of Na^+^ClO_2_^−^ in organic solvents (>0.46 mmol L^−1^ in ethyl acetate), Na^+^ was replaced with Bu_4_N^+^, which exhibits enhanced solubility in organic media. Na^+^ClO_2_^−^ was combined with excess Bu_4_N^+^Cl^−^ at a molar ratio exceeding 5 : 1. The mixture was dissolved in water and stirred at 25 °C for 2 h. The aqueous solution was then extracted with chloroform to isolate the organic phase. The product was then dried under reduced pressure at 25 °C for 24 h to yield Bu_4_N^+^ClO_2_^−^ as a white solid (Scheme 1). The thermal stability of Bu_4_N^+^ClO_2_^−^ was investigated by thermogravimetry‐differential thermal analysis. A rapid weight loss occurred from 198 °C, confirming an exothermic reaction due to pyrolysis at 242 °C. Thus, it is a stable compound under the conditions used in organic synthesis.

The solubility of ClO_2_^−^ salt in organic solvents was considerably improved by counter‐cation exchange of Bu_4_N^+^ to be 5.2 mol L^−1^ in chloroform and 2.8 mol L^−1^ in ethyl acetate. Further experiments involved the dissolution of Bu_4_N^+^ClO_2_^−^ in ethyl acetate. Upon addition of 0.8 equivalents of an ethyl acetate solution of HCl to the Bu_4_N^+^ClO_2_^−^ solution, an immediate change to yellow was observed [Equation 1].

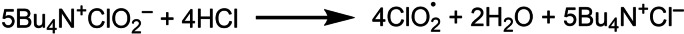




**Figure 1:**
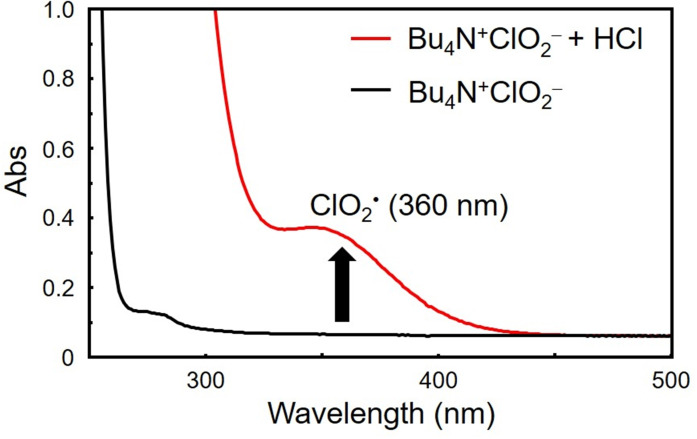
UV‐Vis absorption spectral change for generation of ClO_2_⋅ in the reaction of Bu_4_N^+^ClO_2_^−^ (1.8 mM) with HCl (1.4 mM) in ethyl acetate at 298 K.

Ultraviolet‐visible (UV‐Vis) absorption spectroscopic measurements revealed the generation of ClO_2_⋅, with a distinct absorption peak at 360 nm, indicating its formation and stability under these conditions (Figure 1).^[20]^

### Oxygenation of Sulfides to Sulfoxide with Bu_4_N^+^ClO_2_^−^

Using diphenyl sulfide (**1 a**) as the substrate, the reaction was conducted with 0.9 equivalents of Bu_4_N^+^ClO_2_^−^ and 0.7 equivalents of HCl in an ethyl acetate solution at 25 °C. Diphenyl sulfoxide (**2 a**) was selectively obtained in a high yield of 99 % within 10 min (Table 1, entry 1). The over‐oxidised product, diphenyl sulfone (**3 a**), was not observed under these conditions. This indicates the successful control of the oxidation process. ClO_2_⋅ was generated in an amount corresponding to 80 % of the added Bu_4_N^+^ClO_2_^−^ and in an equimolar ratio with HCl, as indicated in Equation (1). Consequently, 0.7 equivalents of ClO_2_⋅ was formed relative to the substrate under these conditions, and the reaction proceeded to completion in less than 1 equivalent of oxidant. This observation indicates the involvement of a radical chain mechanism with molecular oxygen or the in‐situ generation of a secondary oxidant from chlorine dioxide, which subsequently drives oxidation.

**Table 1:**
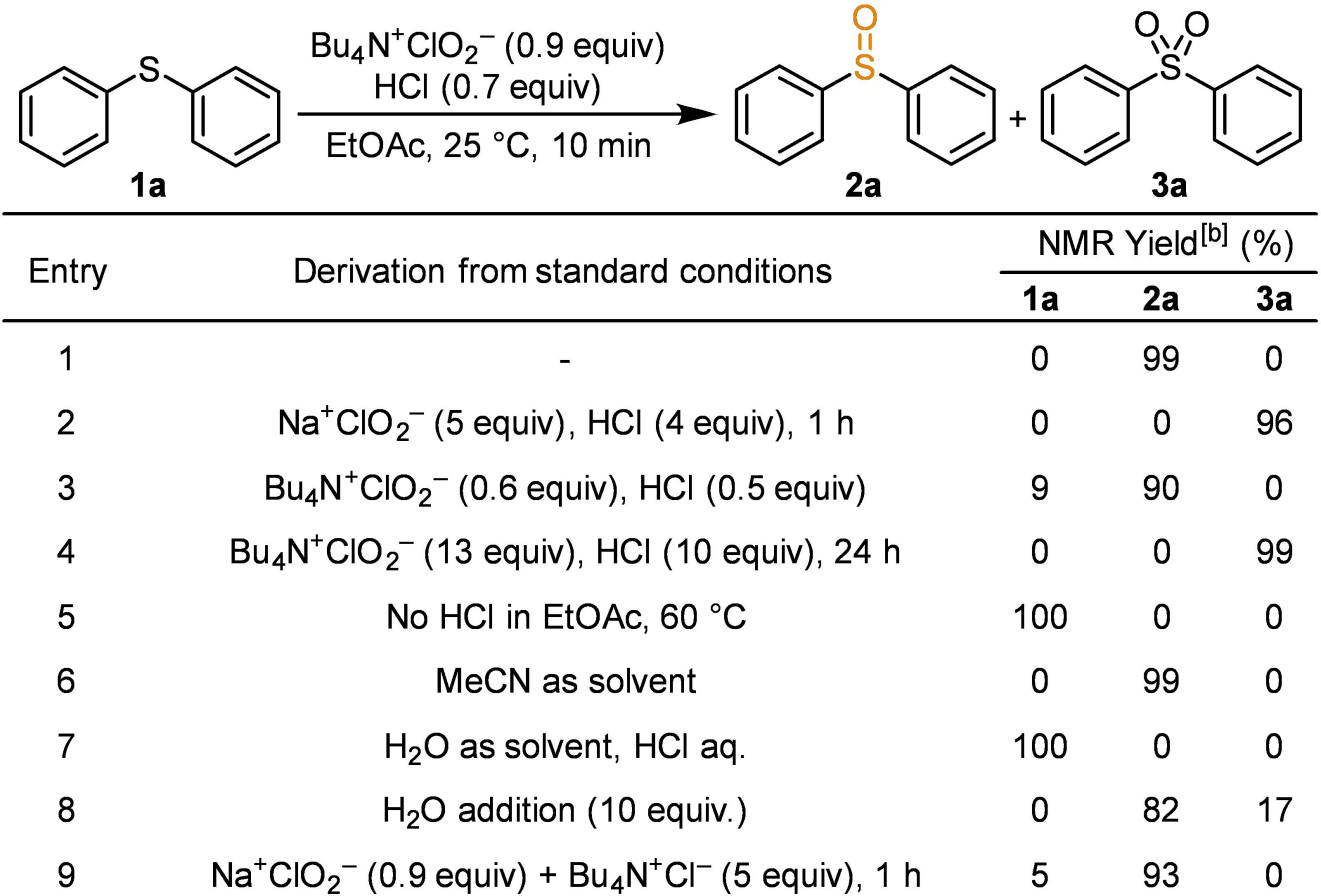
Optimisation of the reaction conditions.^[a]^

[a] Reaction conditions represent modifications instead of the standard conditions. [b] Determined using 1,1,2,2‐tetrachloroethane as an internal standard.

When Na^+^ClO_2_^−^ was employed in place of Bu_4_N^+^ClO_2_^−^ in the presence of HCl in organic media, the reaction did not occur to completion with equimolar amounts of the reagents due to the insolubility of Na^+^ClO_2_^−^, affording sulfone **3 a**. However, when the reaction was conducted with an excess of reagents for 1 h, sulfide **1 a** was fully consumed, forming sulfone **3 a** as the sole product in high yield (entry 2). When the amounts of Bu_4_N^+^ClO_2_^−^ and HCl in an ethyl acetate solution were reduced to 0.6 and 0.5 equivalents, respectively, sulfoxide **2 a** was obtained at 90 %; however, the small amount of sulfide **1 a** remained (entry 3). Using more than 13 equivalents of Bu_4_N^+^ClO_2_^−^ and 10 equiv. of HCl followed by stirring for 24 h led to complete oxidation of sulfide **1 a** to sulfone **3 a** with a yield of 99 % (entry 4). In the absence of HCl, no oxidation occurred, even upon heating (entry 5). Changing the solvent to acetonitrile did not affect the reaction progress (entry 6). These results suggest that the solvent does not directly participate in the reaction mechanism; rather, it serves as a medium to exclude water and maintain a suitable reaction environment. However, no reaction occurred in aqueous media (entry 7). Under these aqueous conditions, when water‐soluble bis(hydroxyethyl)sulfide was treated with NaClO_2_ and HCl, the substrate was fully consumed. However, the reaction produced the sulfoxide in only 48 % yield along with 44 % of the sulfone, resulting in reduced overall selectivity. Adding 10 equivalents of water to the standard conditions resulted in excessive oxidation, producing 17 % of sulfone **3 a** and decreased selectivity (entry 8). By replacing Bu_4_N^+^ClO_2_^−^ with 0.9 equivalents of Na^+^ClO_2_^−^ and 5 equivalents of Bu_4_N^+^Cl^−^, the reaction yielded 93 % of sulfoxide **2 a** in 1 h, and 5 % of sulfide **1 a** was recovered (entry 9).

**Figure 2:**
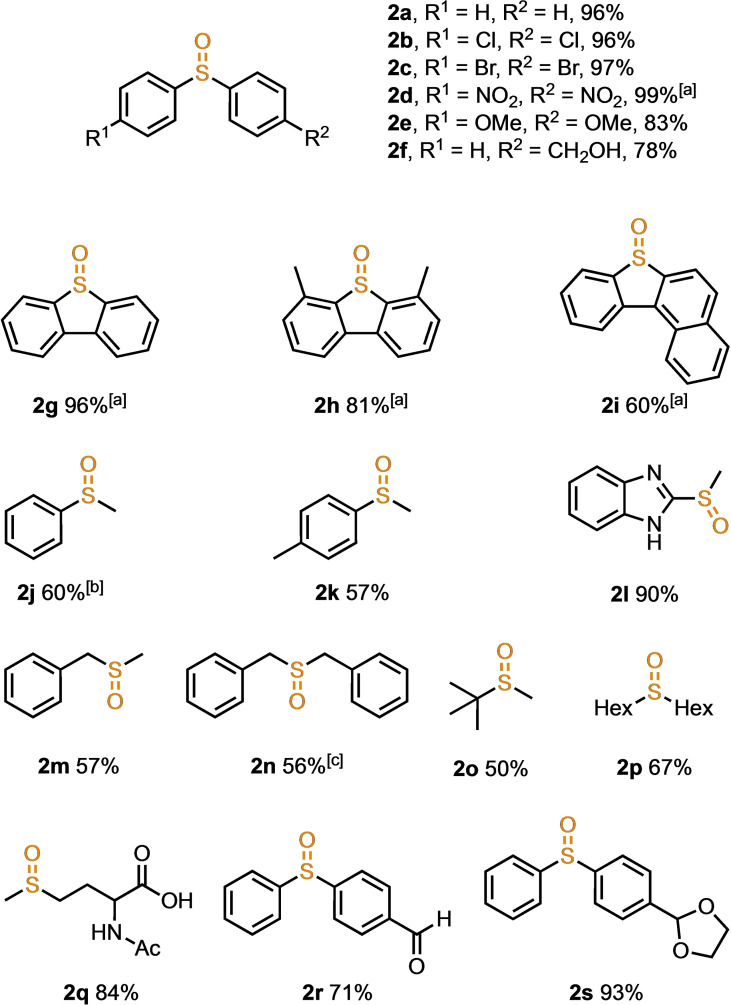
Substrate scope for the oxygenation of sulfides with Bu_4_N^+^ClO_2_^−^. [a] Reaction was conducted using Bu_4_N^+^ClO_2_^−^ (1.9 equiv), HCl (1.5 equiv) for 3 h. [b] Diphenyl disulfide was obtained in 20 % as a byproduct. [c] Benzoic acid was obtained in 32 % as a byproduct.

The reaction was carried out using 0.5 mmol of **1 a**, providing the desired compound **2 a** in 96 % isolated yield (Figure 2). Scaling up the reaction was also successful, as **2 a** was obtained in 95 % yield from 1.0 g of **1 a**. When *para*‐substituted diaryl sulfides were used, the chloro‐ and bromo‐substituted substrates reacted similarly to the diphenyl sulfide, yielding products **2 b** and **2 c** in high yields. For a substrate bearing a nitro group, the reaction required 1.9 equivalents of Bu_4_N^+^ClO_2_^−^ and 1.5 equivalents of HCl, extending to over 3 h to achieve 99 % yield of **2 d**. High yields were achieved using methoxy‐ and hydroxymethyl‐substituted substrates, producing **2 e** and **2 f**. Dibenzothiophene yielded **2 g** efficiently by increasing the amount of reagent and the reaction time. Dimethylbenzothiophene also resulted in **2 h** in high yield under the same conditions, whereas benzonaphthothiophene produced **2 i** in 60 %. Using monoaryl sulfide substrates with a methyl substituent led to complete consumption of the starting material; phenyl substrate produced **2 j** in 60 % yield, whereas diphenyl disulfide was obtained as byproduct in 20 % yield. *p*‐tolyl substrates resulted in similar outcomes, affording **2 k** in moderate yield. In contrast, benzimidazole‐substituted substrate reacted quantitatively, yielding **2 l** at 90 %. Benzyl‐substituted substrates afforded **2 m** and **2 n** in moderate yields. Benzoic acid was obtained as a byproduct from **1 n** in 32 %, indicating that C−S bond cleavage of alkyl‐substituted sulfides during oxidation contributed to the reduced yields. Other dialkyl sulfides yielded **2 o** at 50 % and **2 p** in 67 %. Using acetyl‐protected methionine as a substrate resulted in 84 % yield of **2 q**, demonstrating the applicability of the process for oxidising biomolecules like proteins and peptides. Although formyl groups are typically oxidised to carboxylic acids by hypochlorous acid, as seen in the Pinnick oxidation,[^21^, ^22^, ^23^] the formyl group remained unoxidised in this reaction, selectively oxidising the sulfur to yield oxide **2 r** in 71 %. The substrate with acetal protection of the formyl group afforded **2 s** in 93 % yield without deprotection due to nonaqueous conditions.

### Oxygenation of Polyphenylene Sulfide (PPS)

**Scheme 2:**
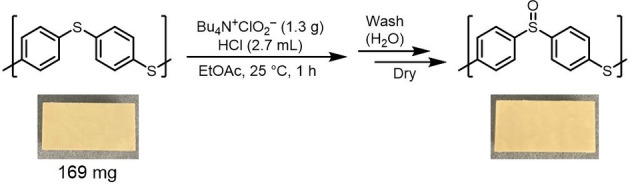
Surface oxidation of a polyphenylene sulfide plate (PPS) with Bu_4_N^+^ClO_2_^−^ and HCl in EtOAc.

**Figure 3:**
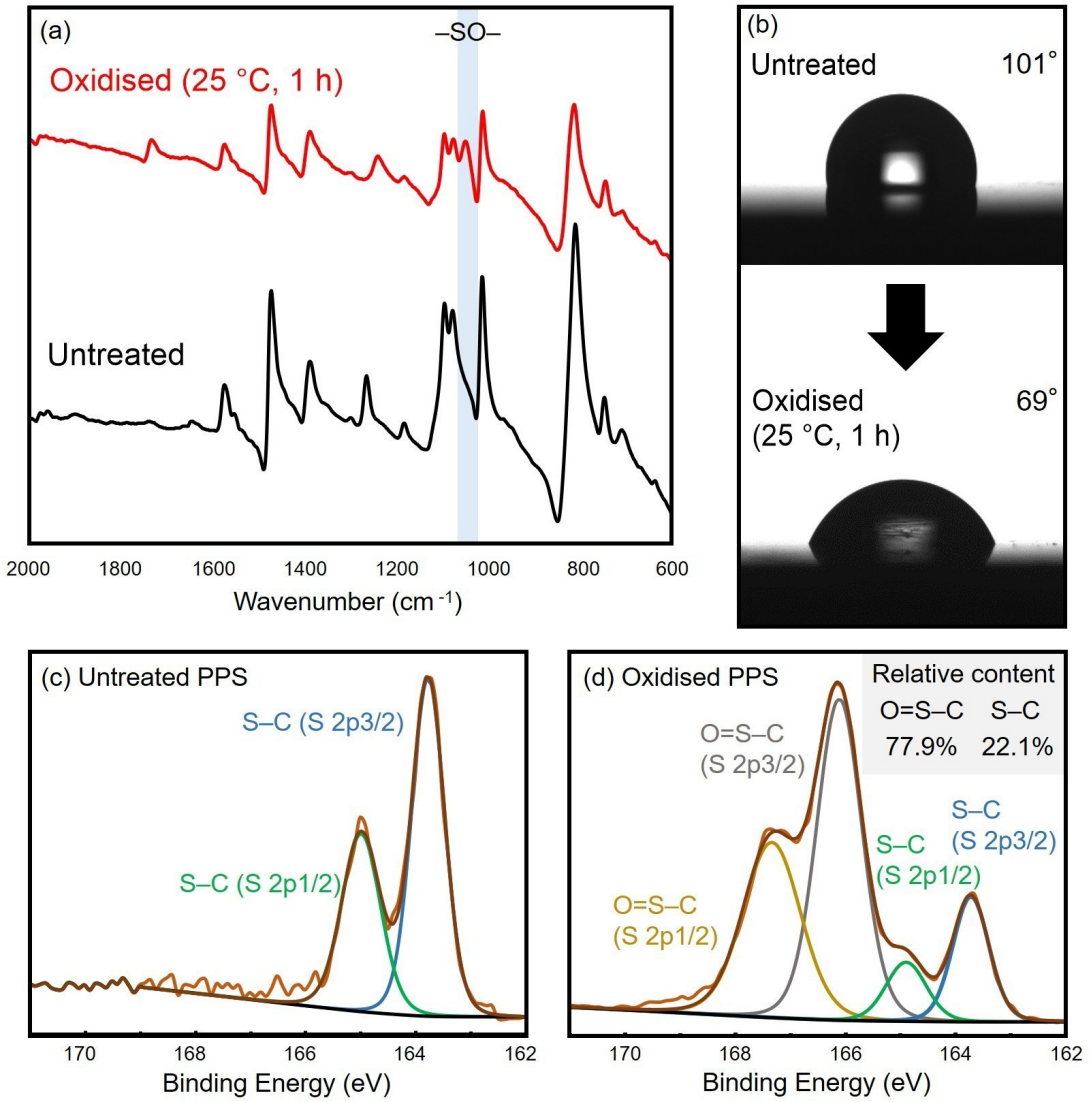
Analyses for surface oxidation of PPS plate: [a] FT‐IR spectra (ATR); [b] water contact angle; [c] XPS spectra for the untreated PPS; [d] XPS spectra for the oxidised PPS.

Polyphenylene sulfide (PPS) is widely used as a versatile polymer; however, its surface is hydrophobic. Utilising this reaction for hydrophilisation offers several advantages without compromising the strength of the material.[^24^, ^25^] It enhances the antifouling properties, preventing the adhesion of hydrophobic substances, and reducing the maintenance costs for materials exposed to external environments. In addition, the improved wettability of the surface aids in the uniform application of paints and facilitates the spread of liquids on the surface. These benefits are crucial for enhancing the functionality of polymeric materials for industrial applications. PPS was immersed for 1 h in the same reaction solution used for the low molecular weight compounds, washed with water, and dried (Scheme 2). Post‐reaction Fourier‐transform infrared (FT‐IR) spectroscopy revealed a peak at 1050 cm^−1^ due to the SO groups. Furthermore, measurements of the water contact angle on the surface showed a decrease from 101° to 69° owing to the oxidation reaction, as shown in Figure 3, indicating successful hydrophilisation. The surface subjected to oxidation was analysed using X‐ray photoelectron spectroscopy (XPS). Prior to oxidation, the binding energies of S were observed at 164 eV for S−C (S 2p3/2) and 165 eV for S−C (S 2p1/2). After oxidation, new peaks corresponding to O=S−C were observed at 166 eV (S 2p3/2) and 167 eV (S 2p1/2). These results indicate that the ratio of surface oxidation, defined as SO/S, was 77.9/22.1, demonstrating that most of the surface sulfur was converted to the sulfoxide form.

### Reaction Mechanism Based on Theoretical Calculations

**Scheme 3:**
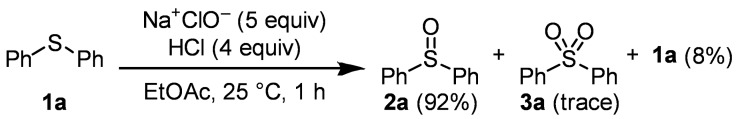
Oxidation of diphenyl sulfide by Na^+^ClO^−^.

**Scheme 4:**
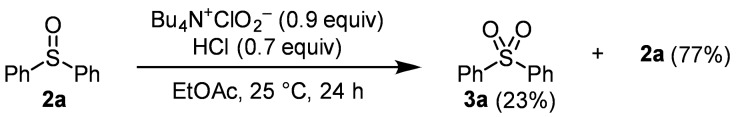
Oxidation of diphenyl sulfoxide.

When sulfide **1 a** was reacted in the presence of HCl and sodium hypochlorite (Na^+^ClO^−^), despite using an excess of reagents and extended reaction time, only a small amount of sulfone **3 a** was obtained, with 8 % recovery of the starting material and 92 % yield of sulfoxide **2 a** (Scheme 3). The detailed experimental procedures are shown in the supporting information (SI) S9. This indicates that the generated chlorine dioxide reacts with the substrate, and the subsequent hypochlorous acid formed is capable of oxidising sulfide **1 a** but has little influence on the further oxidation of the sulfoxide. Thus, the oxidation of sulfoxide **2 a** to sulfone **3 a** was predominantly attributed to excess ClO_2_⋅. The oxidation to the second stage was considerably slower than that to the first, as evidenced by only 23 % progression of the reaction using sulfoxide **2 a** as the substrate over 24 h, demonstrating the selectivity of this reaction (Scheme 4).

Reactivity comparisons were made by reducing the reagent to 0.2 equivalents and conducting the reaction under standard conditions in air (Table 2, entry 1) in an inert argon atmosphere (entry 2), and in an oxygen atmosphere (entry 3). No differences in reactivity were observed under standard conditions or in argon or oxygen atmospheres. This reaction does not involve electron‐transfer oxidation mediated by molecular oxygen but is a system where ClO_2_⋅ alone acts as the oxidant. This is because the free energy change associated with electron transfer is negative (Δ*G*_et_=−0.73 eV), determined from the one‐electron reduction potential of ClO_2_⋅ (*E*_red_ = +0.70 V vs. SCE)^[26]^ and one‐electron oxidation potential of diphenyl sulfide (*E*_ox_ = +1.43 V vs. SCE).^[27]^

**Table 2:**
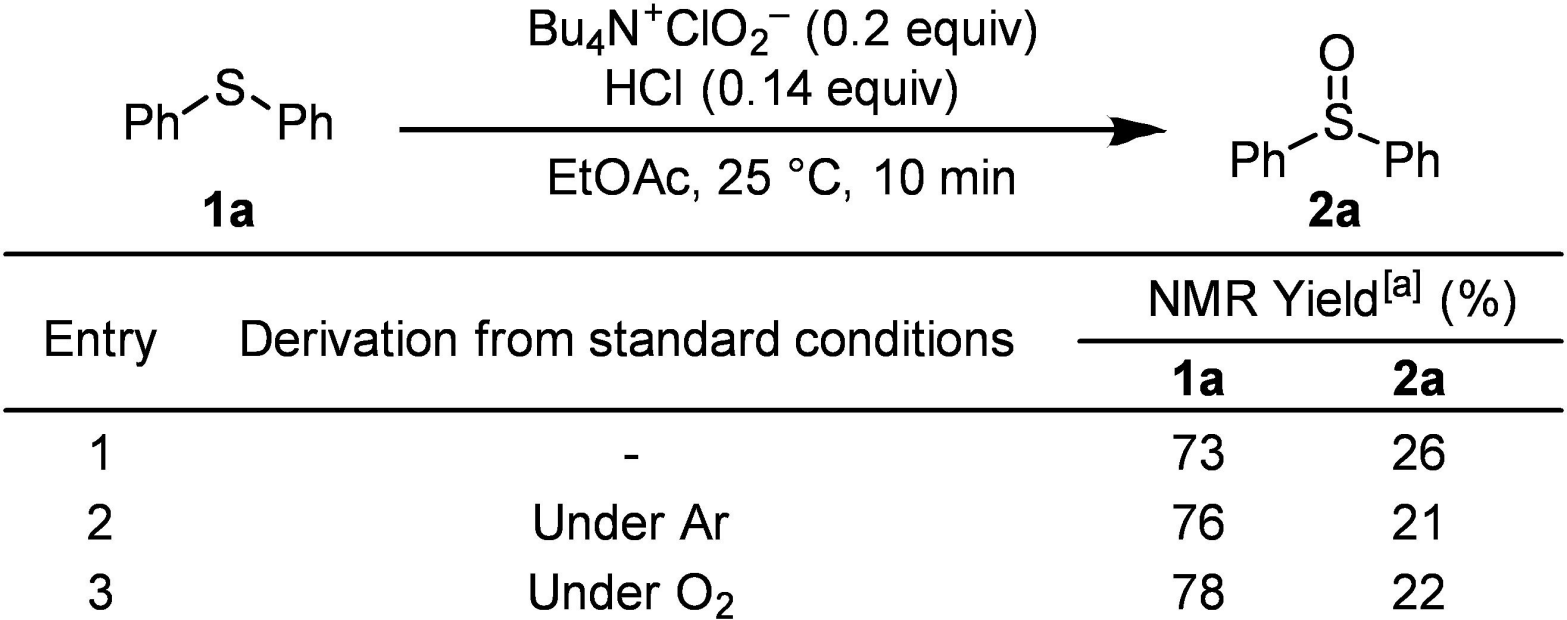
Oxidation under Ar or O_2_ atmosphere.

[a] Determined by use of 1,1,2,2‐tetrachloroethane as an internal standard.

**Figure 4:**
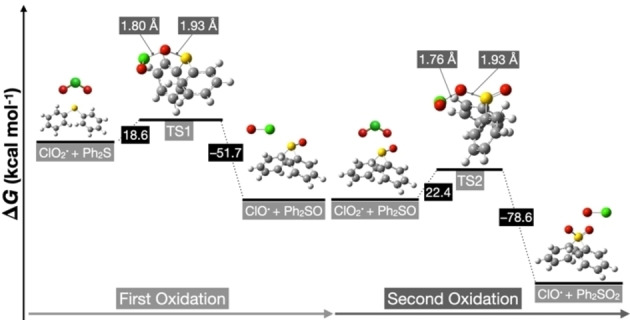
DFT calculation analyses for oxidation of sulfide to sulfoxide and sulfone.

The density functional theory (DFT) calculations were performed at the UM06–2X/6–311+G (2d, p) level of theory using the SMD solvation model in acetonitrile (Figure 4). When sulfide **1 a** reacts with ClO_2_⋅, a transition state (TS1) is formed, in which the sulfur atom of sulfide **1 a** bonds with the oxygen atom of ClO_2_⋅. This transition state exists at an energy level 18.6 kcal mol^−1^ higher than the reactant system. In TS1, the O−S and Cl−O bond lengths are 1.93 Å and 1.80 Å, respectively. The cleavage of the Cl−O bond leads to the formation of chlorine monoxide radical (ClO⋅) and sulfoxide **2 a**, and the reaction proceeds exothermically from TS1 by −51.7 kcal mol^−1^.

In the second oxidation step, starting from sulfoxide **2 a** and ClO_2_⋅, the transition state (TS2) involves a similar bonding interaction with the sulfur atom of sulfoxide **2 a** and the oxygen atom of ClO_2_⋅, positioned at an energy level 22.4 kcal mol^−1^ higher than the reactant system. The bond lengths in TS2 are 1.93 Å for O−S and 1.76 Å for Cl−O, with the Cl−O bond being 0.04 Å shorter than that of TS1. The energy level of TS2 is 3.8 kcal mol^−1^ higher than that of TS1. Thus, while both oxidation steps have sufficient activation energy to proceed at 25 °C, the first oxidation occurs at a faster rate owing to smaller activation energy, resulting in the selective formation of the first‐stage product. The reaction involving TS2 also proceeds exothermically by −51.7 kcal/mol, with the cleavage of the Cl−O bond leading to the formation of chlorine monoxide radical and sulfone **3 a**.

**Figure 5:**
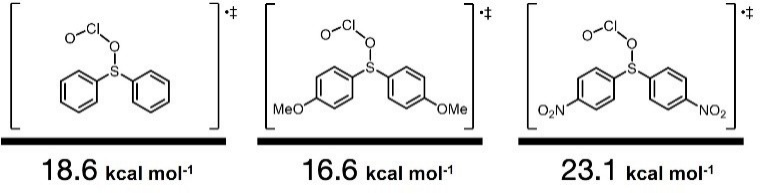
Comparison of TS1 energies of diphenyl sulfide with ClO_2_⋅, based on DFT calculations.

Furthermore, an investigation on the substituent effects at the para position relative to sulfur in TS1 revealed that the activation energy was 16.6 kcal mol^−1^ for the *para*‐OMe substrate and 23.1 kcal mol^−1^ for the *para*‐NO_2_ substrate (Figure 5). Electron‐donating groups lower the activation energy, whereas electron‐withdrawing groups increase it, indicating that the electronic state of the S atom determines the reactivity. This finding is consistent with experimental results showing that the *para*‐NO_2_ substituted substrate requires an extended reaction time of 3 h and an increased number of equivalents of reagents compared to the standard conditions for the reaction to complete.

**Scheme 5:**
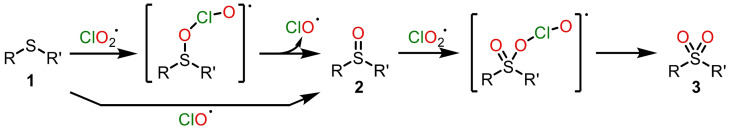
Proposed mechanism for the oxygenation of sulfides.

The overall reaction mechanism is illustrated in Scheme 5. Chlorine dioxide is generated in situ via the reaction between Bu_4_N^+^ClO_2_^−^ and HCl. Next, it directly reacts with sulfide **1** to produce sulfide **2**, concurrently producing chlorine monoxide (ClO⋅).^[28]^ This product further reacts with another molecule of sulfide **1** to yield a second equivalent of sulfoxide **2**. Thus, a single molecule of chlorine dioxide functions as a dual oxidant, completing the reaction with less than one equivalent of the oxidant. While excess chlorine dioxide can overoxidise the resulting sulfoxide **2** to sulfone **3**, under stoichiometric conditions, quantum chemical calculations indicate that the oxidation transition state (TS1) for sulfide oxidation is lower in energy than the corresponding transition state (TS2) for sulfoxide oxidation. This energy difference allows the first oxidation to proceed selectively and rapidly, preventing over‐oxidation and yielding sulfoxide **2** as the exclusive product.

## Conclusions

In summary, the highly selective oxidation of sulfides to sulfoxides under mild conditions was successfully achieved by employing Bu_4_N^+^ClO_2_^−^ in organic media instead of Na^+^ClO_2_^−^ in aqueous or mixed solvents as the chlorine dioxide source. This method could be applied to the selective oxidation of a variety of sulfides, including a sulfide with formyl group and *N*‐acetylmethionine, in good to excellent yields. In addition, PPS surface was hydrophilised by utilising this method. Therefore, the use of Bu_4_N^+^ClO_2_^−^ for organic solvent applications is an efficient and highly selective approach for selective oxidation.

## Experimental Section

### Procedure for the Synthesis of Bu_4_N^+^ClO_2_^−^

To a 500 mL round‐bottom flask, Bu_4_N^+^Cl^−^ (23.1 g, 83.1 mmol), Na^+^ClO_2_^−^ (57.5 g, 508 mmol), and distilled water (250 mL) were added, and the mixture was stirred for 2 h at 25 °C. The reaction mixture was extracted with chloroform (250 mL ×3), and the organic layer was washed with a Na^+^ClO_2_^−^ saturated aqueous solution (250 mL). It was concentrated under reduced pressure at 40 °C to 25 °C for 24 h to produce Bu_4_N^+^ClO_2_^−^ as a white solid (28.6 g).

### General Procedure for the Synthesis of 2 a

To a 50 mL screw vial, diphenyl sulfide **1 a** (93.1 mg, 0.50 mmol), Bu_4_N^+^ClO_2_^−^ (170 mg, 0.45 mmol), ethyl acetate (12 mL) and HCl in ethyl acetate solution (350 μL, 0.35 mmol) were added, and the mixture was stirred for 10 min at 25 °C. The reaction was quenched with aqueous Na_2_S_2_O_3_ solution (6 mL, 0.7 mmol), and extracted with ethyl acetate (12 mL ×2). The organic layer was washed with brine (12 mL), and dried over anhydrous Na_2_SO_4_. It was concentrated under reduced pressure to afford the desired product **2 a** as a white solid (97.0 mg, 0.48 mmol, 96 % yield). The detailed spectral data (^1^H NMR, ^13^C NMR and HRMS) summarised in SI† S17–43.

### Theoretical Calculations

The density functional theory (DFT) calculations were performed by Gaussian 16 (Revision C.02; Gaussian Inc., Wallingford, CT, USA) with a 52‐processor HPC5000‐XIL216TS−D8. The functional and basis set were UM06–2X and 6–311+G (2d, p) with the SMD solvation model in acetonitrile. Optimized ground‐state geometries were examined by frequency analysis to possess no negative frequency. Optimized transition states geometries were examined by frequency analysis to possess only one imaginary frequency. For each transition states, intrinsic reaction coordinate (IRC) analysis was performed to ensure that it connects the reactant and product. The Cartesian coordinates are shown in SI S7–S16.

## Conflict of Interests

The authors declare no conflict of interest.

1

## Supporting information

As a service to our authors and readers, this journal provides supporting information supplied by the authors. Such materials are peer reviewed and may be re‐organized for online delivery, but are not copy‐edited or typeset. Technical support issues arising from supporting information (other than missing files) should be addressed to the authors.

Supporting Information

## Data Availability

The data that support the findings of this study are available in the supplementary material of this article.
