# Supplementary material for: Tetrabutylammonium Chlorite as an Efficient Oxidant for Controlled Oxidation of Sulfides to Sulfoxides
Source: Chemistry. 2025 Feb 18;31(17):e202404279. doi: 10.1002/chem.202404279 (PMC11924991; doi:10.1002/chem.202404279)
Supplement: Supplementary file 1 — Supporting Information [file CHEM-31-e202404279-s001.pdf]

Chemistry–A European Journal

Supporting Information

Tetrabutylammonium Chlorite as an Efficient Oxidant for Controlled Oxidation of Sulfides to Sulfoxides

Yuki Itabashi, Shuto Ogata, Yusuke Shimada, Minato Kondo, Nagatoshi Nishiwaki, Tsuyoshi Inoue, Haruyasu Asahara,* and Kei Ohkubo*

Supporting Information (SI)

Tetrabutylammonium Chlorite as an Efficient Oxidant for Controlled Oxidation of Sulfides to Sulfoxides

Yuki Itabashi,[a] Shuto Ogata,[b] Yusuke Shimada,[b] Minato Kondo,[c] Nagatoshi Nishiwaki,[c] Tsuyoshi Inoue,[a,b] Haruyasu Asahara,*[a,b] and Kei Ohkubo*[a]

[a] Institute for Open and Transdisciplinary Research Initiatives (OTRI), Osaka University, 1-6 Yamadaoka, Suita, Osaka 565-0871, Japan. E-mail: ohkubo@irdd.osaka-u.ac.jp
[b] Graduate School of Pharmaceutical Sciences, Osaka University, 1-6 Yamadaoka, Suita, Osaka 565-0871, Japan. E-mail: asahara@phs.osaka-u.ac.jp
[c] School of Engineering Science, Kochi University of Technology, Tosayamada, Kami, Kochi 782-8502, Japan.

Table of contents

1.	General Information	S3
2.	Preparation for UV-Vis absorption spectroscopy	S3
3.	Synthetic procedure of sulfides	S3
4.	General procedure for the selective oxidation of sulfides	S4
5.	Procedure for the surface oxidation of PPS	S5
6.	Mechanistic experiments	S5
7.	DFT calculation	S7
8.	Spectral data	S16
9.	NMR spectra	S22
10.	References for SI	S44

1. General Information

Unless otherwise noted, all materials were purchased from Aldrich Inc., Tokyo Chemical Industry, Wako Chemicals, and other commercial suppliers and were used without further purification. NMR spectra were recorded by a Bruker AVANCE NEO 400 spectrometer, with chemical shifts calibrated using residual undeuterated solvent ($CHCl_3$ at 7.26 ppm for ^{1}H NMR, 77.16 ppm for ^{13}C NMR; DMSO at 2.50 ppm for ^{1}H NMR, 39.52 ppm for ^{13}C NMR). The following abbreviations indicate the multiplicities: s, singlet. d, doublet. t, triplet. q, quartet. m, multiplet. br, broad. IR spectra were recorded by a Perkin Elmer Spectrum Two FTIR equipped with a diamond window. All spectra were acquired at 8 cm^{-1} resolutions over 8 scans in the scan range of 500–4000 cm^{-1}. High-resolution mass spectra were obtained using an AB SCEIX Triplet TOF 4600 mass spectrometer. The density functional theory (DFT) calculations were performed using Gaussian 16 (Revision C.02; Gaussian Inc., Wallingford, CT, USA) with a 52-processor HPC5000-XIL216TS-D8. UV-Vis absorption spectroscopy analysis was carried out with a JASCO V-750 UV-Vis Spectrophotometer. The water contact angle (WCA) was determined using the sessile drop method with a Kyowa Interface Science Drop Master DM300 averaging the results of five measurements. XPS spectra were recorded using a Ulvac-PHI ESCA 3057 instrument.

2. UV-Vis absorption spectral measurements

UV-Vis spectral measurements were carried out to confirm the generation of chlorine dioxide in the reaction of tetrabutylammonium chlorite with hydrogen chloride. The spectra were recorded on a JASCO V-750 UV-Vis Spectrophotometer. To a 9 mL screw-cap vial, tetrabutylammonium chlorite (68 mg, 0.18 mmol), hydrogen chloride in ethyl acetate solution (140 μL, 0.14 mmol), ethyl acetate (5 mL) were added, and the mixture was stirred for 10 minutes at 25 °C. After the solution was diluted sevenfold, UV-Vis spectroscopic measurements were performed at 298 K (Fig. 1, $Bu_4N^+ClO_2^-$ + HCl). For comparison, UV-Vis spectroscopy of tetrabutylammonium chlorite only was also conducted (Fig. 1, $Bu_4N^+ClO_2^-$).

3. Synthetic procedures of sulfides

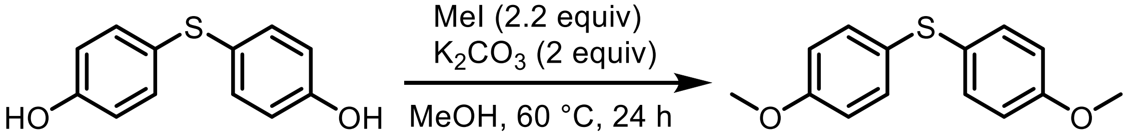

1e

Bis(4-methoxyphenyl) sulfide[1,2]: To a 30 mL test tube, bis(4-hydroxyphenyl) sulfide (1.31 g, 6.0 mmol), potassium carbonate (1.70 g, 12 mmol), methanol (12 mL), and iodomethane (1.85 g, 13 mmol) were added, and the mixture was stirred for 24 hours at 60 °C. The reaction was quenched with water (28 mL) and aqueous 36% hydrogen chloride solution (430 μL). The solution was extracted with

chloroform (28 mL × 2), and the combined organic layers were dried over anhydrous Na_2SO_4. The solvent was removed under reduced pressure, and the residue was purified by silica gel column chromatography (hexane/ethyl acetate = 5:1) to afford the desired product **1e** as a white solid (1.12 g, 4.5 mmol, 76% yield). **^{1}H NMR (400 MHz, $CDCl_3$)** δ(ppm): 7.28 (d, *J* = 8.8 Hz, 4H), 6.84 (d, *J* = 8.8 Hz, 4H), 3.79 (s, 6H). **^{13}C NMR (100 MHz, $CDCl_3$)** δ(ppm): 159.0, 132.8, 127.5, 114.8, 55.4. **HRMS (MALDI)**: calcd for $C_{14}H_{14}O_2S$ $[M+H]^+$: 246.0709, found: 246.0711.

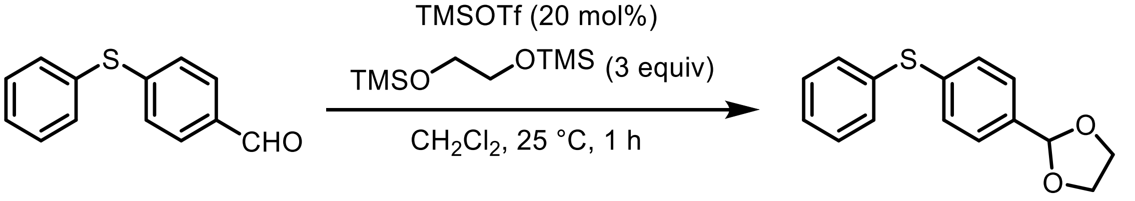

1s

2-(4-(phenylthio)phenyl)-1,3-dioxolane[3]: To a 7 mL test tube, 4-(phenylthio) benzaldehyde (254 mg, 1.2 mmol), dichloromethane (4.7 mL), trimethylsilyl trifluoromethanesulfonate (53.0 mg, 0.24 mmol), and ethylenedioxybis(trimethylsilane) (743 mg, 3.6 mmol) were added, and the mixture was stirred for 1 hour at 25 °C. The reaction was quenched by the addition of anhydrous pyridine (80 μL) at 0 °C, and the solution was poured into saturated aqueous $NaHCO_3$ solution (5 mL). The organic layer was extracted with chloroform (5 mL × 2), and dried over anhydrous Na_2SO_4. The solvent was removed under reduced pressure. To decompose any remaining reagent, the residue was dissolved in methanol (2 mL), K_2CO_3 were added, and the mixture was stirred for 3 hours at 25 °C. The reaction mixture was poured into saturated aqueous $NaHCO_3$ solution (5 mL). The solution was extracted with chloroform (5 mL × 2), and the combined organic layers were dried over anhydrous Na_2SO_4. The solvent was removed under reduced pressure, and the residue was purified by silica gel column chromatography (hexane/ethyl acetate = 10:1) to afford the desired product **1s** as a yellow oil (293 mg, 1.1 mmol, 94% yield). **^{1}H NMR (400 MHz, $CDCl_3$)** δ(ppm): 7.45–7.20 (m, 9H), 5.78 (s, 1H), 4.14–4.01 (m, 4H). **^{13}C NMR (100 MHz, $CDCl_3$)** δ(ppm): 137.3, 136.7, 135.3, 131.5, 130.6, 129.3, 127.4, 103.4, 65.4. **HRMS (MALDI)**: calcd for $C_{15}H_{15}O_2S$ $[M+H]^+$: 259.0787, found: 259.0789.

4. General procedure for the selective oxidation of sulfides

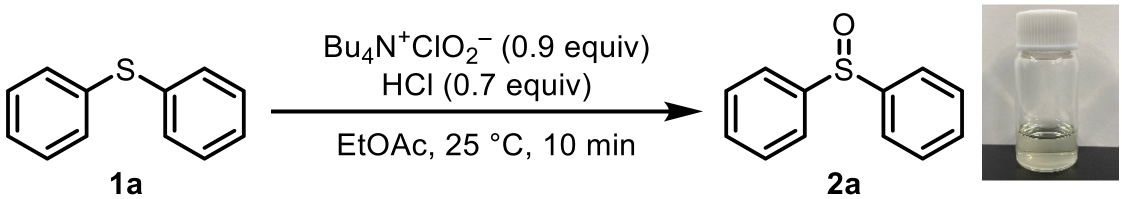

1a **2a**

General procedure for the synthesis of 2a: To a 50 mL screw-cap vial, diphenyl sulfide **1a** (93.1 mg, 0.50 mmol), tetrabutylammonium chlorite (170 mg, 0.45 mmol), ethyl acetate (12 mL) and hydrogen

chloride in ethyl acetate solution (350 µL, 0.35 mmol) were added, and the solution was stirred for 10 minutes at 25 °C. The reaction was quenched by the addition of an aqueous $Na_2S_2O_3$ solution (6 mL, 0.70 mmol), and extracted with ethyl acetate (12 mL × 2). The combined organic layers were washed with brine (12 mL), and dried over anhydrous Na_2SO_4. The solvent was removed under reduced pressure to afford the desired product **2a** as a white solid (97.0 mg, 0.48 mmol, 96 % yield).

General procedure for the synthesis of 2a on a gram-scale: To a 100 mL round-bottom flask, diphenyl sulfide **1a** (1.00 g, 5.4 mmol), tetrabutylammonium chlorite (1.72 g, 4.7 mmol), ethyl acetate (30 mL) and hydrogen chloride in ethyl acetate solution (3.38 mL, 3.8 mmol) were added, and the solution was stirred for 1 hour at 25 °C. The solvent was removed under reduced pressure, and the residue was purified by silica gel column chromatography (ethyl acetate) to afford the desired product **2a** as a white solid (1.04 g, 5.1 mmol, 95 % yield).

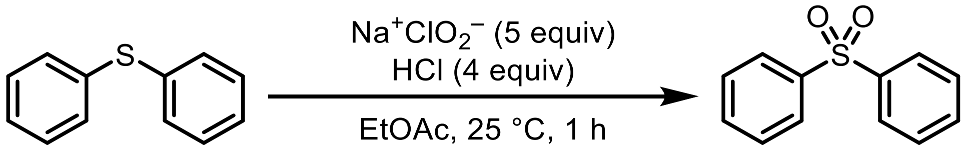

1a **3a**

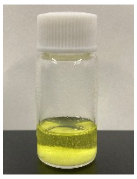

General procedure for the synthesis of 3a: To a 30 mL screw vial, diphenyl sulfide **1a** (37.3 mg, 0.20 mmol), sodium chlorite (112 mg, 1.0 mmol), ethyl acetate (4 mL), and hydrogen chloride in ethyl acetate solution (800 µL, 0.80 mmol) were added, and the mixture was stirred for 1 hour at 25 °C. The reaction was quenched by the addition of an aqueous $Na_2S_2O_3$ solution (3 mL, 1.6 mmol), and extracted with ethyl acetate (5 mL × 3). The combined organic layers were washed with brine (5 mL), and dried over anhydrous Na_2SO_4. The solvent was removed under reduced pressure to afford the desired product **3a** as a white solid (41.4 mg, 0.19 mmol, 95% yield).

5. Procedure for the surface oxidation of PPS

The PPS plate (PPS 800, 10 × 120 × 120 mm, Takiron Polymer Co., Ltd.) was used. To a 30 mL sample vial, PPS (169 mg), tetrabutylammonium chlorite (1.3 g), ethyl acetate (3 mL), and hydrogen chloride in ethyl acetate solution (2.7 mL) were added, and the mixture was stirred for 1 hour at 25 °C. The oxidised PPS was washed with water, and dried under vacuum.

6. Mechanistic experiments

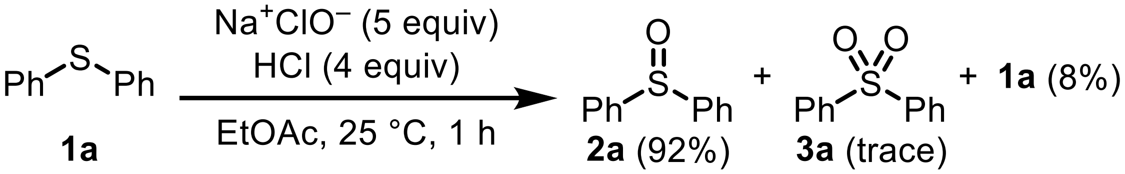

1a **2a** (92%) **3a** (trace)

To a 30 mL screw-cap vial, diphenyl sulfide **1a** (36.9 mg, 0.20 mmol), sodium hypochlorite (89.0 mg, 1.2 mmol), ethyl acetate (3 mL), and hydrogen chloride in ethyl acetate solution (800 µL, 0.80 mmol) were added, and the solution was stirred for 1 hour at 25 °C. The reaction was quenched by the addition of an aqueous $Na_2S_2O_3$ solution (3 mL, 1.6 mmol), and extracted with ethyl acetate (5 mL × 2). The combined organic layers were washed with brine (5 mL), and dried over anhydrous Na_2SO_4. The solution was concentrated under reduced pressure, and the NMR yield was determined using 1,1,2,2-tetrachloroethane as an internal standard.

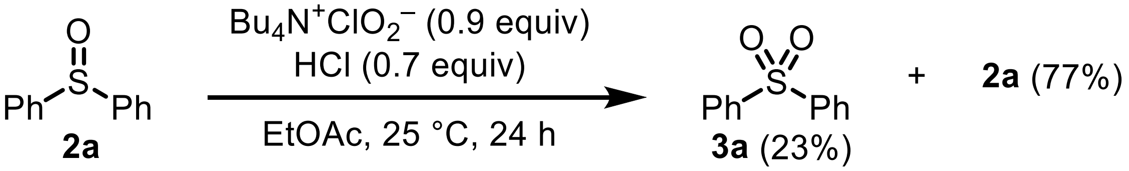

To a 30 mL screw-cap vial, diphenyl sulfoxide **2a** (40.5 mg, 0.20 mmol), tetrabutylammonium chlorite (68 mg, 0.18 mmol), ethyl acetate (5 mL), and hydrogen chloride in ethyl acetate solution (140 µL, 0.14 mmol) were added, and the solution was stirred for 24 hours at 25 °C. The reaction was quenched by the addition of an aqueous $Na_2S_2O_3$ solution (3 mL, 0.30 mmol), and extracted with ethyl acetate (5 mL × 2). The combined organic layers were washed with brine (5 mL), and dried over anhydrous Na_2SO_4. The solution was concentrated under reduced pressure, and the NMR yield was determined using of 1,1,2,2-tetrachloroethane as an internal standard.

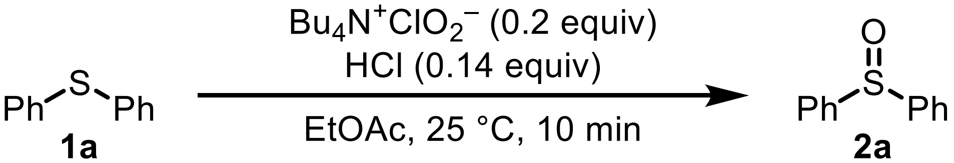

Entry	Derivation from standard conditions	NMR Yield (%)	
		**1a**	**2a**
1	-	73	26
2	Under Ar	76	21
3	Under O_2	78	22

To a 30 mL screw-cap vial, diphenyl sulfide **1a** (186 mg, 1.0 mmol), tetrabutylammonium chlorite (68.0 mg, 0.18 mmol), ethyl acetate (25 mL), and hydrogen chloride in ethyl acetate solution (140 µL, 0.14 mmol) were added, and the solution was stirred for 10 minutes at 25 °C under air, argon, or oxygen. The reaction was quenched by the addition of an aqueous $Na_2S_2O_3$ solution (15 mL, 0.30 mmol), and extracted with ethyl acetate (25 mL × 2). The combined organic layers were washed with brine (25 mL) and dried over anhydrous Na_2SO_4. The solution was concentrated under reduced pressure, and the NMR yield was determined using of 1,1,2,2-tetrachloroethane as an internal standard.

7. Theoretical calculations

The theoretical calculations were performed by Gaussian 16 (Revision C.02; Gaussian Inc., Wallingford, CT, USA)[4] using UM06-2X/6-311+G (2d, p) level theory with the SMD solvation model in acetonitrile. Optimized ground-state geometries were examined by frequency analysis to possess no negative frequency. Optimized transition states geometries were examined by frequency analysis to possess only one imaginary frequency. For each transition states, intrinsic reaction coordinate (IRC) analysis was performed to ensure that it connects the reactant and product.

Cartesian coordinates

First oxidation reactant (figure 4) $E = -1471.8582$ Hartree

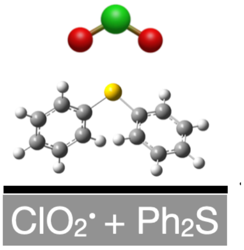

S	25.79115401	-0.19491531	-1.45364237
O	-74.31417457	-1.19076507	-0.48023179
Cl	-74.32518410	0.02985562	0.35521988
O	-74.79414098	1.26434787	-0.31125899
C	25.06018755	-1.44017356	-0.40754869
C	24.33816409	-2.46373801	-1.01407378
C	25.25208760	-1.44435929	0.97302750
C	23.81442594	-3.49394586	-0.24018069
H	24.18050145	-2.45374670	-2.08606507
C	24.70807137	-2.46449459	1.74012991
H	25.82119349	-0.65243703	1.44551790
C	23.99156575	-3.49434966	1.13699317
H	23.25509659	-4.28873689	-0.71856846
H	24.85437745	-2.46000137	2.81353450
H	23.57363463	-4.29114583	1.73971513
C	25.53472000	1.30239353	-0.51987034
C	24.29570482	1.60506602	0.04301876
C	26.58700113	2.20668828	-0.41731537
C	24.12121274	2.80401767	0.71835503
H	23.47396822	0.90382716	-0.04389977
C	26.39758785	3.41678400	0.24265200
H	27.55219535	1.96546749	-0.84638482
C	25.16932078	3.71538856	0.81650637
H	23.15906198	3.03235249	1.16077990
H	27.21971514	4.11817714	0.31741034
H	25.02614855	4.65301785	1.33926336

First oxidation product (figure 4) $E = -1471.9077$ Hartree

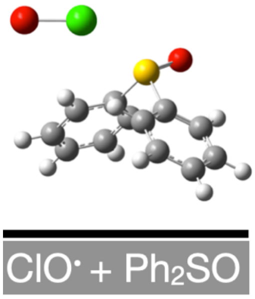

S	18.38209074	-0.00026684	1.49149768
O	16.87982686	-0.00090887	1.46719046
Cl	-80.22553953	0.00011213	-0.40538455
O	-81.21908357	-0.00040690	0.81662556
C	18.90926516	1.35012153	0.41646262
C	17.98488575	1.91797852	-0.44497720
C	20.21906590	1.80214852	0.49002833
C	18.39197413	2.95992300	-1.26985056
H	16.96561041	1.55057172	-0.45807997
C	20.61637102	2.84067574	-0.34292477
H	20.92025465	1.35606166	1.18690315
C	19.70457539	3.41701550	-1.22132523
H	17.68239819	3.41564246	-1.94949149
H	21.63516078	3.20502709	-0.29864968
H	20.01657057	4.23067516	-1.86451092
C	18.91040559	-1.35016159	0.41639245
C	17.98628712	-1.91918156	-0.44454438
C	20.22075324	-1.80069462	0.48943840
C	18.39417911	-2.96077438	-1.26946997
H	16.96659896	-1.55292230	-0.45717721
C	20.61885524	-2.83888096	-0.34355342
H	20.92175529	-1.35371408	1.18593325
C	19.70731552	-3.41637129	-1.22147339
H	17.68480917	-3.41739784	-1.94872202
H	21.63807438	-3.20207895	-0.29968781
H	20.01994063	-4.22976608	-1.86468817

Second oxidation reactant (figure 4) $E = -1547.0441$ Hartree

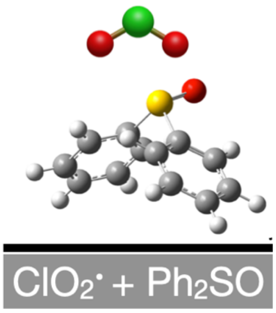

S	23.11658535	-0.92789469	0.93802993
O	-77.21998686	-1.29532310	-0.47157079
Cl	-77.03783770	-0.08340647	0.35674478
O	-77.31023063	1.20542894	-0.31609065

C	22.80534605	0.70876026	0.24412324
C	23.18674388	1.82586974	0.96923179
C	22.17413675	0.81278599	-0.98714358
C	22.94257621	3.08527384	0.43462208
H	23.65849286	1.70328544	1.93693747
C	21.94041446	2.07668420	-1.51469516
H	21.86532939	-0.07524063	-1.52773457
C	22.32476367	3.20975399	-0.80521360
H	23.23355204	3.96959289	0.98818970
H	21.44948519	2.17579637	-2.47482602
H	22.13509236	4.19339550	-1.21699753
C	24.69755027	-1.21460922	0.11579404
C	24.70701286	-1.72797962	-1.17341941
C	25.86769447	-0.93496918	0.80216826
C	25.92852255	-1.94548329	-1.79809597
H	23.77845554	-1.95803588	-1.68462109
C	27.08442044	-1.16173514	0.16924942
H	25.81873441	-0.55434829	1.81543976
C	27.11428922	-1.66178416	-1.12792968
H	25.95371990	-2.34440565	-2.80452406
H	28.00922204	-0.94873642	0.69130805
H	28.06467960	-1.83848075	-1.61645234
O	23.42650383	-0.72185611	2.39366043

Second oxidation product (figure 4) $E = -1547.1336$ Hartree

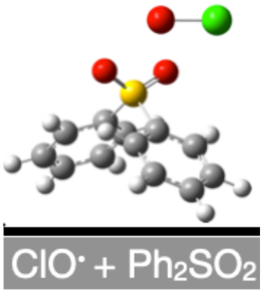

S	-17.22978257	-0.91401912	-0.39480492
O	-16.47245658	-1.66561136	0.58705398
Cl	82.39683422	-0.55133669	-0.15563458
O	82.66174987	0.91528418	0.35365905
C	-16.91582711	0.81092446	-0.12319859
C	-17.07180998	1.69780278	-1.17995630
C	-16.54624389	1.23586144	1.14608453
C	-16.84708375	3.04918714	-0.95503368
H	-17.35431089	1.33470291	-2.16020888
C	-16.32472188	2.58978051	1.35751970
H	-16.42612563	0.51917337	1.94901968
C	-16.47627624	3.49217895	0.31001412
H	-16.95828395	3.75489254	-1.76852189
H	-16.03032211	2.93893576	2.33913350
H	-16.30107539	4.54741922	0.48012486
C	-18.95305680	-1.10866611	-0.02069635
C	-19.32897077	-1.41236921	1.28089185
C	-19.88594845	-0.92745253	-1.03306186

C	-20.68035585	-1.54016829	1.57194438
H	-18.57734678	-1.55469310	2.04728495
C	-21.23399918	-1.05815993	-0.72864824
H	-19.56158518	-0.69795882	-2.04041103
C	-21.62834271	-1.36207905	0.57004222
H	-20.99186133	-1.78215101	2.58018097
H	-21.97545012	-0.92606318	-1.50642930
H	-22.68139702	-1.46377638	0.80219953
O	-17.04630417	-1.18398438	-1.80760258

TS1 (figure 4) *E* = –1471.8286 Hartree, Imaginary frequency = –791.95

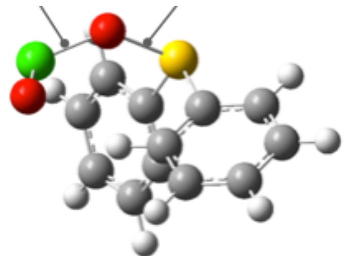

S	0.08046038	-0.20101053	-1.47589583
O	-0.29360084	1.66922016	-1.18075910
Cl	-1.01618112	2.60294811	0.18149586
O	0.04652652	3.08675137	1.20708356
C	-1.22473385	-0.84139992	-0.45571569
C	-2.51787565	-0.43675912	-0.77938319
C	-0.98674726	-1.75712378	0.56231845
C	-3.58507176	-0.95208470	-0.05895420
H	-2.68682129	0.27636456	-1.57753675
C	-2.06658884	-2.26135467	1.27601829
H	0.01931442	-2.07918718	0.79809458
C	-3.36132578	-1.86088482	0.96990064
H	-4.59274896	-0.63847574	-0.30107305
H	-1.88973076	-2.97222282	2.07335263
H	-4.19744886	-2.25809033	1.53178660
C	1.54865483	-0.39957776	-0.51509268
C	1.66656857	0.17839217	0.74617541
C	2.59319063	-1.11568940	-1.09006496
C	2.85188145	0.01466510	1.44559099
H	0.85327514	0.75521202	1.16933495
C	3.77695678	-1.26602645	-0.37857739
H	2.48304765	-1.55280909	-2.07517233
C	3.90409850	-0.70569587	0.88584503
H	2.95736775	0.45737245	2.42814392
H	4.59615056	-1.82295861	-0.81527776
H	4.82785614	-0.82569159	1.43829095

TS2 (figure 4) *E* = –1547.0084 Hartree, Imaginary frequency = –684.29

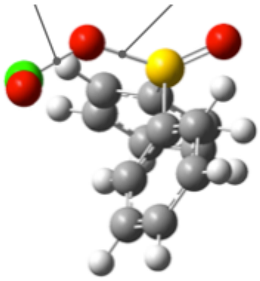

S	0.22942505	-0.12826078	1.27676641
O	-0.36348667	1.69355423	1.03867521
Cl	-1.17142785	2.52843420	-0.28879259
O	-2.69810016	2.33137055	-0.19834075
C	1.73208775	-0.13395921	0.32252048
C	2.25981221	1.03878600	-0.19521214
C	2.35542102	-1.36747437	0.15959903
C	3.44768384	0.96644079	-0.91327311
H	1.76820460	1.98776684	-0.03377140
C	3.53814340	-1.42027646	-0.56014281
H	1.92216407	-2.26553406	0.58344223
C	4.08086350	-0.25578918	-1.09660227
H	3.87713831	1.87092242	-1.32453913
H	4.03544734	-2.37110554	-0.70344159
H	5.00577926	-0.30341554	-1.65788857
C	-0.99533275	-0.85055422	0.20716778
C	-0.93972785	-0.65094156	-1.16594990
C	-2.02691508	-1.55084884	0.81529268
C	-1.95904168	-1.17314122	-1.94978314
H	-0.11672745	-0.11498965	-1.62260942
C	-3.03784930	-2.06665065	0.01527154
H	-2.03701304	-1.69422160	1.88827758
C	-3.00551125	-1.87462635	-1.36085214
H	-1.93037172	-1.03430842	-3.02301359
H	-3.85021533	-2.61818926	0.47104155
H	-3.79722123	-2.27856004	-1.97957311
O	0.39514745	-1.03434509	2.43654940

TS1 (*p*-MeO-C_6H_4)$_2$–S (Figure 5) $E = -1700.8672$ Hartree, Imaginary frequency = –795.99

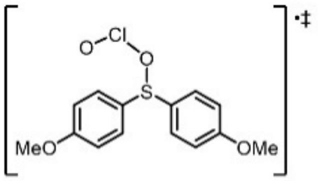

S	-0.04860366	0.56562521	1.68027337
O	0.19877849	2.33155562	0.92146124
Cl	0.83140657	2.95082835	-0.64798803
O	-0.27570878	3.07633749	-1.73456911
C	1.30517648	-0.22122938	0.85260686
C	2.56004509	0.35956244	0.98537780
C	1.15368845	-1.42497804	0.16495305
C	3.67390395	-0.24701978	0.42122127
H	2.67750411	1.29346329	1.52225018

C	2.25891283	-2.02821145	-0.39922067
H	0.18147371	-1.89213403	0.07091207
C	3.52430634	-1.44509007	-0.27797494
H	4.64087148	0.22332286	0.52938408
H	2.16191610	-2.96056101	-0.94129357
C	-1.47662490	0.03552989	0.80535912
C	-1.59210800	0.21327745	-0.57615554
C	-2.51754959	-0.51993381	1.53674967
C	-2.74421173	-0.18730601	-1.21365617
H	-0.79092995	0.67239385	-1.14261821
C	-3.68407729	-0.91950720	0.89710529
H	-2.42578272	-0.65058886	2.60849521
C	-3.79722611	-0.75658257	-0.48294927
H	-2.85964646	-0.06021202	-2.28294238
H	-4.48442337	-1.35260298	1.47958420
O	4.53866570	-2.10992842	-0.86627818
O	-4.87956021	-1.11237307	-1.19892598
C	5.84157977	-1.54509448	-0.77507123
H	6.49961993	-2.22601681	-1.30871984
H	5.87244615	-0.56045983	-1.24631897
H	6.16072112	-1.46799978	0.26636693
C	-5.98011371	-1.68462040	-0.50024645
H	-6.73541761	-1.89194156	-1.25372616
H	-5.68717257	-2.61423252	-0.00818352
H	-6.37904388	-0.98402822	0.23613614

TS1 (p-NO_2-C_6H_4)$_2$–S (Figure 5) $E = -1880.8217$ Hartree, Imaginary frequency = –787.32

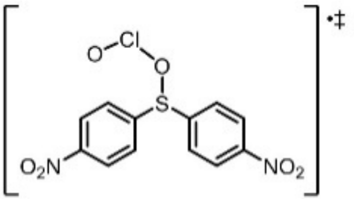

S	-0.02622711	1.03992892	1.67017437
O	0.12124364	2.61818789	0.60259578
Cl	0.77121384	2.96681905	-1.04934221
O	-0.28629391	2.72491322	-2.15459360
C	1.34088070	0.16498323	0.94970708
C	2.57386304	0.81279415	0.99156978
C	1.20894449	-1.12549588	0.44918106
C	3.69571065	0.16251650	0.50969757
H	2.66063629	1.81634302	1.38900420
C	2.33142710	-1.77582208	-0.03629225
H	0.25268752	-1.63116917	0.43738882
C	3.54864237	-1.11826497	0.00319553
H	4.66348758	0.64292666	0.52387933
H	2.25886379	-2.77807381	-0.43365851
C	-1.45876299	0.30030434	0.94813698
C	-1.61224404	0.24161000	-0.43456635
C	-2.43966240	-0.15998689	1.82016749
C	-2.76654188	-0.31399349	-0.95571264
H	-0.85239053	0.63194294	-1.10003769

C	-3.60039368	-0.70930958	1.29897917
H	-2.30314213	-0.09893739	2.89244183
C	-3.73280916	-0.77775775	-0.07626392
H	-2.91779184	-0.37754378	-2.02390600
H	-4.37992195	-1.07960752	1.94909854
N	4.73939373	-1.81208241	-0.51018036
N	-4.96061396	-1.37074486	-0.63378719
O	4.59739863	-2.93591576	-0.94809431
O	5.80134631	-1.22441449	-0.46761178
O	-5.04975259	-1.45928793	-1.84128507
O	-5.81634383	-1.73977568	0.14433811

First ClO$^{\bullet}$ oxidation reactant $E = -1396.7218$ Hartree

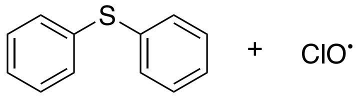

S	19.01191391	-0.05691528	-1.24602473
O	-77.94091836	-0.76788149	-0.06341083
Cl	-79.05470629	0.33978231	0.05061502
C	19.85660303	-1.40347228	-0.43837255
C	19.13357784	-2.55592255	-0.14673521
C	21.22150790	-1.34571698	-0.16066249
C	19.77779738	-3.65266973	0.41678959
H	18.07074539	-2.59570385	-0.35375328
C	21.85192401	-2.43571511	0.42134858
H	21.78603350	-0.45094595	-0.39565500
C	21.13397188	-3.59373903	0.70779847
H	19.21043782	-4.54803360	0.64005786
H	22.91128214	-2.38424131	0.64206633
H	21.63181972	-4.44426346	1.15682396
C	19.61496033	1.37110094	-0.36496078
C	19.72770662	1.38023406	1.02437569
C	19.91103722	2.51756234	-1.09615380
C	20.14988979	2.53170787	1.67286810
H	19.48926830	0.49020846	1.59479348
C	20.31289961	3.67435023	-0.43642123
H	19.83328225	2.50625178	-2.17690369
C	20.43944521	3.68331828	0.94621435
H	20.24279691	2.53222120	2.75221351
H	20.54026194	4.56426130	-1.01070898
H	20.76287961	4.58141446	1.45776042

First ClO$^{\bullet}$ oxidation product $E = -1396.7506$ Hartree

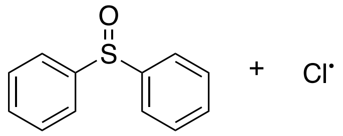

S	-14.01912291	-0.02149558	1.60932013
O	-12.81044461	-0.68660730	2.20436765
Cl	86.93066561	-0.09817818	-0.02109381
C	-14.65696157	-1.15225627	0.35492812
C	-13.86825352	-2.21831229	-0.04470651
C	-15.92724727	-0.94366409	-0.16363469

C	-14.36216820	-3.09202953	-1.00662394
H	-12.88992301	-2.35892777	0.39910440
C	-16.40847164	-1.81997612	-1.12795700
H	-16.53572422	-0.11335290	0.17800660
C	-15.62665126	-2.89112942	-1.54888848
H	-13.75873458	-3.93116929	-1.33041151
H	-17.39735951	-1.67106415	-1.54349045
H	-16.00832247	-3.57564690	-2.29644285
C	-13.40364691	1.23778166	0.47255944
C	-12.07627679	1.19161950	0.07850255
C	-14.26516821	2.23384534	0.03566988
C	-11.60353826	2.16511604	-0.79338734
H	-11.42944704	0.40950845	0.45795390
C	-13.78323358	3.19860209	-0.84040163
H	-15.29530523	2.26219501	0.37356453
C	-12.45570482	3.16311288	-1.25415124
H	-10.56847973	2.14455847	-1.11157144
H	-14.44242312	3.98274251	-1.19156890
H	-12.08213944	3.92071446	-1.93206776

First ClO• oxidation TS *E* = –1396.7203 Hartree, Imaginary frequency = –698.80

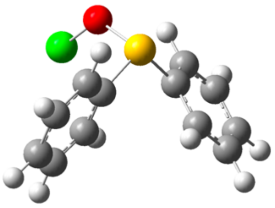

S	0.04864510	0.29839433	-1.37858369
O	-0.08515324	2.11493416	-0.75286915
Cl	-0.20259814	2.71479504	0.94664618
C	-1.36127980	-0.39611352	-0.54220600
C	-2.47151884	0.40880165	-0.32113244
C	-1.37088603	-1.75071275	-0.22117946
C	-3.60910843	-0.15658777	0.24330123
H	-2.44093012	1.45945950	-0.57708239
C	-2.51029688	-2.29809820	0.34935516
H	-0.50141645	-2.36895306	-0.40959800
C	-3.62865579	-1.50332913	0.58148252
H	-4.47811084	0.46377939	0.42349717
H	-2.52416635	-3.34929681	0.60843816
H	-4.51600365	-1.93684578	1.02585719
C	1.43643910	-0.27073093	-0.44842206
C	1.38288809	-0.45251357	0.93172299
C	2.61631559	-0.47454637	-1.16017364
C	2.53037035	-0.85450563	1.59867185
H	0.46197273	-0.28487380	1.47534548
C	3.75759915	-0.87032916	-0.47706113
H	2.64096762	-0.32879136	-2.23379792
C	3.71510632	-1.06266499	0.89879558
H	2.49898154	-1.00164866	2.67099453
H	4.67776857	-1.03389354	-1.02363527
H	4.60617267	-1.37625194	1.42836077

Second ClO$^•$ oxidation reactant *E* = −1471.9077 Hartree

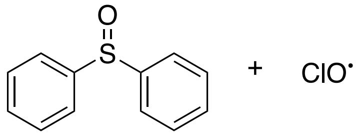

S	-19.29910914	-0.03421246	1.58763859
O	82.64022030	-0.77382480	0.73385613
Cl	82.97024116	0.34111795	-0.32843582
C	-19.53284403	-1.35762197	0.38363581
C	-18.42016409	-1.93248798	-0.21333080
C	-20.82028053	-1.78229092	0.09770770
C	-18.60907910	-2.94914910	-1.14152392
H	-17.42049779	-1.59791967	0.04155878
C	-20.99706711	-2.80356802	-0.82791871
H	-21.66259942	-1.32190046	0.60041555
C	-19.89471147	-3.38242131	-1.44812458
H	-17.75154848	-3.40856098	-1.61720371
H	-21.99651116	-3.14816249	-1.06343820
H	-20.03733950	-4.17936648	-2.16757994
C	-19.27586021	1.33027310	0.40620653
C	-18.08760429	1.65143252	-0.23473767
C	-20.44420579	2.03988089	0.18301623
C	-18.07998324	2.70259068	-1.14270803
H	-17.17975325	1.09446059	-0.02963335
C	-20.42366810	3.09323468	-0.72397164
H	-21.34690947	1.77123701	0.71874272
C	-19.24631757	3.42066987	-1.38738291
H	-17.16141800	2.96553783	-1.65247930
H	-21.32832487	3.65874960	-0.91089072
H	-19.23472166	4.24353498	-2.09158498
O	-20.58997169	0.08476599	2.34715344

Second ClO$^•$ oxidation product *E* = −1471.9765 Hartree

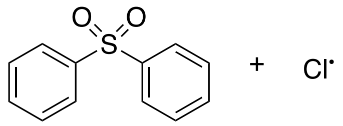

S	13.25449613	-0.09219730	1.30241903
O	12.20749762	0.24379857	2.24764900
Cl	-87.33577989	0.07537728	0.00388519
C	13.49757629	1.30652430	0.23850607
C	12.45636946	2.20835510	0.06477161
C	14.71460776	1.45236715	-0.41403635
C	12.64516430	3.28868986	-0.78646915
H	11.52126125	2.07058087	0.59312189
C	14.89009389	2.53665811	-1.26278410
H	15.51049791	0.73585225	-0.25311338
C	13.85770845	3.45018376	-1.44807806
H	11.84661228	4.00559203	-0.92964872
H	15.83407133	2.67004251	-1.77585313
H	14.00010470	4.29514592	-2.11054202
C	12.63300449	-1.35815402	0.22595917
C	11.26318473	-1.45179613	0.01881422

C	13.53226270	-2.20888682	-0.40267017
C	10.78438452	-2.42968415	-0.84219200
H	10.58624876	-0.77789047	0.52893329
C	13.04027586	-3.18277986	-1.26108486
H	14.59468270	-2.11538376	-0.21543232
C	11.67116123	-3.29064223	-1.48013185
H	9.71903811	-2.52114548	-1.01197203
H	13.72619526	-3.85897766	-1.75544527
H	11.29228726	-4.05260682	-2.15021123
O	14.55382170	-0.51660816	1.78632391

Second ClO$^{•}$ oxidation TS E = –1471.9001 Hartree, Imaginary frequency = –493.70

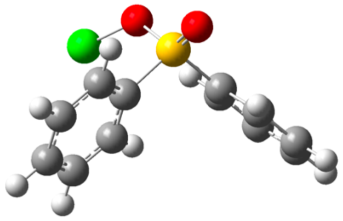

S	0.05458847	0.05608685	1.24892088
O	-0.17986085	1.96740021	0.99671361
Cl	-0.98428672	2.84179756	-0.30438942
C	1.54906481	-0.20646155	0.31807387
C	2.11550624	0.81824848	-0.42680967
C	2.11557187	-1.47452837	0.39237964
C	3.28370925	0.55226617	-1.13056462
H	1.66073973	1.79893929	-0.45272957
C	3.28281335	-1.71947260	-0.31414509
H	1.65347522	-2.24976031	0.99088725
C	3.86354920	-0.70898516	-1.07474619
H	3.74188966	1.33793967	-1.71763069
H	3.73834498	-2.70056503	-0.27054824
H	4.77617705	-0.90661026	-1.62339828
C	-1.25391116	-0.51137420	0.18062288
C	-1.17833744	-0.34245969	-1.19534857
C	-2.35328237	-1.09249709	0.79670837
C	-2.24129773	-0.77902325	-1.97378748
H	-0.30948549	0.10734561	-1.65905534
C	-3.40880513	-1.52090049	0.00280010
H	-2.37854930	-1.21671196	1.87164374
C	-3.35289500	-1.36362088	-1.37689792
H	-2.19655427	-0.66328905	-3.04919148
H	-4.27249906	-1.98075601	0.46592697
H	-4.17774002	-1.70228125	-1.99155405
O	0.06655394	-0.83481858	2.43301437

8. Spectral data

Diphenyl sulfoxide (**2a**)[5]

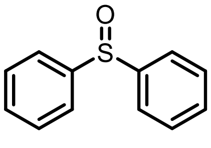

The title compound was prepared according to the general procedure. White solid (97.0 mg, 0.48 mmol, 96 % yield). **^{1}H NMR (400 MHz, CDCl$_3$)** δ (ppm): 7.74–7.56 (m, 4H), 7.54–7.35 (m, 6H). **^{13}C NMR (100 MHz, CDCl$_3$)** δ (ppm): 145.6, 131.1, 129.4, 124.8. **HRMS (MALDI)**: calcd for $C_{12}H_{11}OS$ $[M+H]^+$: 203.0525, found: 203.0524.

Bis(4-chlorophenyl) sulfoxide (**2b**)[6]

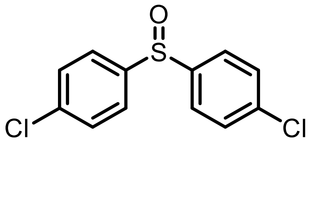

The title compound was prepared according to the general procedure. White solid (130 mg, 0.48 mmol, 96% yield). **^{1}H NMR (400 MHz, CDCl$_3$)** δ (ppm): 7.56 (d, *J* = 8.8 Hz, 4H), 7.43 (d, *J* = 8.8 Hz, 4H). **^{13}C NMR (100 MHz, CDCl$_3$)** δ (ppm): 143.8, 137.7, 129.8, 126.1. **HRMS (MALDI)**: calcd for $C_{12}H_9OSCl_2$ $[M+H]^+$: 270.9746, found: 270.9744.

Bis(4-bromophenyl) sulfoxide (**2c**)[7]

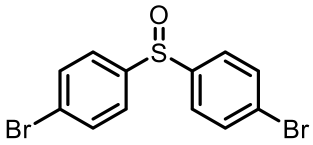

The title compound was prepared according to the general procedure. White solid (174 mg, 0.48 mmol, 97% yield). **^{1}H NMR (400 MHz, CDCl$_3$)** δ (ppm): 7.60 (d, *J* = 8.8 Hz, 4H), 7.49 (d, *J* = 8.8 Hz, 4H). **^{13}C NMR (100 MHz, CDCl$_3$)** δ (ppm): 141.6, 133.3, 129.3, 127.7. **HRMS (MALDI)**: calcd for $C_{12}H_9OSBr_2$ $[M+H]^+$: 360.8715, found: 360.8712.

Bis(4-nitrophenyl) sulfoxide (**2d**)[5]

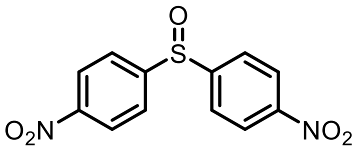

The title compound was prepared according to the general procedure with 1.9 equivalents of $TBAClO_2$ and 1.5 equivalents of hydrogen chloride for 3 hours. Yellow solid (152.0 mg, 0.49 mmol, 99% yield). **^{1}H NMR (400 MHz, CDCl$_3$)** δ (ppm): 8.34 (d, *J* = 9.2 Hz, 4H), 7.88 (d, *J* = 9.2 Hz, 4H). **^{13}C NMR (100 MHz, CDCl$_3$)** δ (ppm): 151.5, 149.8, 125.4, 125.0. **HRMS (MALDI)**: calcd for $C_{12}H_9N_2O_5S$ $[M+H]^+$: 293.0227, found: 239.0222.

Bis(4-methoxyphenyl) sulfoxide (**2e**)[4]

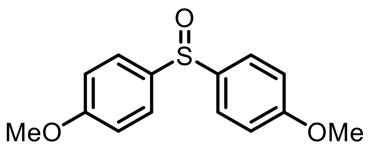

The title compound was prepared according to the general procedure. The mixture was purified by silica gel column chromatography (hexane/ethyl acetate = 2:1). Orange solid (109 mg, 0.42 mmol, 83% yield). **^{1}H NMR (400 MHz, CDCl$_3$)** δ (ppm): 7.52 (d, *J* = 8.8 Hz, 4H), 6.94 (d, *J* = 8.8 Hz, 4H), 3.80 (s, 6H). **^{13}C NMR (100 MHz, CDCl$_3$)** δ (ppm): 161.8, 137.0, 126.9, 114.7, 55.5. **HRMS (MALDI)**: calcd for $C_{14}H_{15}O_3S$ $[M+H]^+$: 263.0736, found:

263.0737.

(4-(phenylsulfinyl)phenyl) methanol (**2f**)[8]

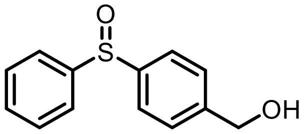

The title compound was prepared according to the general procedure. The mixture was purified by silica gel column chromatography (hexane/ethyl acetate = 2:1). White solid (90.5 mg, 0.39 mmol, 78% yield). **^{1}H NMR (400 MHz, CDCl$_3$)** δ (ppm): 7.64–7.51 (m, 4H), 7.49–7.35 (m, 5H), 4.68 (s, 2H). **^{13}C NMR (100 MHz, CDCl$_3$)** δ (ppm): 145.3, 144.8, 144.2, 131.2, 129.4, 127.6, 125.1, 124.8, 64.3. **HRMS (MALDI)**: calcd for $C_{13}H_{12}O_2NaS$ [M+Na]$^+$: 255.0452, found: 255.0456.

Dibenzothiophene sulfoxide (**2g**)[9]

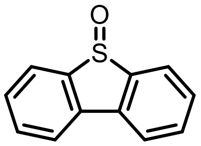

The title compound was prepared according to the general procedure with 1.9 equivalents of TBAClO$_2$ and 1.5 equivalents of hydrogen chloride for 3 hours. White solid (96.1 mg, 0.48 mmol, 96% yield). **^{1}H NMR (400 MHz, CDCl$_3$)** δ (ppm): 8.03–7.94 (m, 2H), 7.84–7.75 (m, 2H), 7.64–7.55 (m, 2H), 7.54–7.45 (m, 2H). **^{13}C NMR (100 MHz, CDCl$_3$)** δ (ppm): 145.1, 137.1, 132.6, 129.6, 127.5, 121.9. **HRMS (MALDI)**: calcd for $C_{12}H_9OS$ [M+H]$^+$: 201.0369, found: 201.0366.

4,6-Dimethyldibenzothiophene sulfoxide (**2h**)[10]

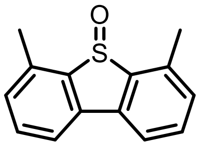

The title compound was prepared according to the general procedure with 1.9 equivalents of TBAClO$_2$ and 1.5 equivalents of hydrogen chloride for 3 hours on 0.30 mmol scale. The mixture was purified by silica gel column chromatography (hexane/ethyl acetate = 3:1). White solid (55.2 mg, 0.24 mmol, 81% yield). **^{1}H NMR (400 MHz, CDCl$_3$)** δ (ppm): 7.57 (d, J = 7.6 Hz, 2H), 7.44 (dd, J = 7.6, 7.6 Hz, 2H), 7.20 (d, J = 7.6 Hz, 2H), 2.72 (s, 6H). **^{13}C NMR (100 MHz, CDCl$_3$)** δ (ppm): 142.7, 139.1, 137.6, 132.7, 131.0, 119.5, 18.7. **HRMS (MALDI)**: calcd for $C_{14}H_{13}OS$ [M+H]$^+$: 229.0681, found: 229.0686.

Benzo[b]naphtho[1,2-d]thiophene sulfoxide (**2i**)[11]

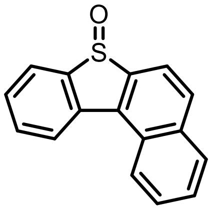

The title compound was prepared according to the general procedure with 1.9 equivalents of TBAClO$_2$ and 1.5 equivalents of hydrogen chloride for 3 hours on 0.20 mmol scale. The mixture was purified by preparative thin-layer chromatography (hexane/ethyl acetate = 2:1). Brown solid (30.0 mg, 0.12 mmol, 60% yield). **^{1}H NMR (400 MHz, CDCl$_3$)** δ (ppm): 8.73 (d, J = 8.4 Hz, 1H), 8.44 (d, J = 8.0 Hz, 1H), 8.07 (d, J = 7.6 Hz, 1H), 8.03 (d, J = 8.8 Hz, 1H), 7.97 (d, J = 8.8 Hz, 2H), 7.73–7.61 (m, 3H), 7.52 (ddd, J = 1.2, 7.6, 7.6 Hz, 1H). **^{13}C NMR (100 MHz, CDCl$_3$)** δ (ppm): 145.9, 144.0, 138.1, 136.1, 132.6, 130.8, 129.8, 129.6, 128.7, 128.3, 127.9, 125.4, 124.5, 122.5. **HRMS (MALDI)**:

calcd for $C_{16}H_{11}OS$ $[M+H]^+$: 251.0525, found: 251.0526.

Methyl phenyl sulfoxide (**2j**)[5]

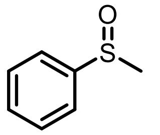

The title compound was prepared according to the general procedure. The mixture was purified by silica gel column chromatography (hexane/ethyl acetate = 1:3). Yellow oil (42.0 mg, 0.30 mmol, 60% yield). **^{1}H NMR (400 MHz, $CDCl_3$)** δ (ppm): 7.73–7.58 (m, 2H), 7.57–7.41 (m, 3H), 2.70 (s, 3H). **^{13}C NMR (100 MHz, $CDCl_3$)** δ (ppm): 145.7, 131.1, 129.4, 123.5, 44.0. **HRMS (MALDI)**: calcd for C_7H_9OS $[M+H]^+$: 141.0369, found: 141.0361.

Methyl 4-tolyl sulfoxide (**2k**)[5]

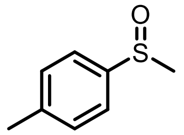

The title compound was prepared according to the general procedure. The mixture was purified by silica gel column chromatography (hexane/ethyl acetate = 1:3). Yellow oil (43.9 mg, 0.29 mmol, 57% yield). **^{1}H NMR (400 MHz, $CDCl_3$)** δ (ppm): 7.53 (d, *J* = 8.0 Hz, 2H), 7.32 (d, *J* = 8.0 Hz, 2H), 2.69 (s, 3H), 2.40 (s, 3H). **^{13}C NMR (100 MHz, $CDCl_3$)** δ (ppm): 142.5, 141.6, 130.1, 123.6, 44.0, 21.5. **HRMS (MALDI)**: calcd for $C_8H_{11}OS$ $[M+H]^+$: 155.0525, found: 155.0524.

2-(Methylsulfinyl)-1*H*-benzimidazole (**2l**)[12]

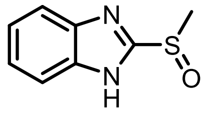

The title compound was prepared according to the general procedure with 1.7 equivalents of hydrogen chloride for 1 hour on 0.60 mmol scale. The mixture was purified by silica gel column chromatography (hexane/ethyl acetate = 1:1). White solid (97.3 mg, 0.54 mmol, 90% yield). **^{1}H NMR (400 MHz, $CDCl_3$)** δ (ppm): 7.79 (d, *J* = 7.6 Hz, 1H), 7.59 (d, *J* = 7.6 Hz, 1H), 7.34 (ddd, *J* = 5.6, 21.2, 21.2 Hz), 3.18 (s, 3H), 1.87 (s, 1H). **^{13}C NMR (100 MHz, $CDCl_3$)** δ (ppm): 153.7, 144.0, 134.4, 124.6, 123.4, 120.3, 112.2, 41.6. **HRMS (MALDI)**: calcd for $C_8H_9N_2OS$ $[M+H]^+$: 181.0431, found: 181.0436.

Benzyl methyl sulfoxide (**2m**)[13]

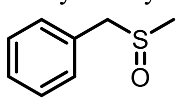

The title compound was prepared according to the general procedure. The mixture was purified by silica gel column chromatography (hexane/ethyl acetate = 1:1). Yellow oil (43.9 mg, 0.29 mmol, 57% yield). **^{1}H NMR (400 MHz, $CDCl_3$)** δ (ppm): 7.39–7.20 (m, 5H), 3.92 (dd, *J* = 12.8, 40.8 Hz, 2H), 2.38 (s, 3H). **^{13}C NMR (100 MHz, $CDCl_3$)** δ (ppm): 129.9, 129.5, 128.8, 128.2, 60.0, 37.1. **HRMS(MALDI)**: $C_8H_{11}OS$ $[M+H]^+$: 155.0525, found: 155.0526.

Dibenzyl sulfoxide (**2n**)[5]

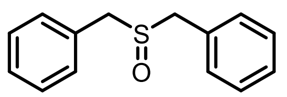

The title compound was prepared according to the general procedure on 0.20 mmol scale. The mixture was purified by preparative thin-layer

chromatography (hexane/ethyl acetate = 1:1). White solid (25.3 mg, 0.11 mmol, 56% yield). **^{1}H NMR (400 MHz, CDCl$_3$)** δ (ppm): 7.50–7.22 (m, 10H), 3.90 (dd, *J* = 9.2, 12.8 Hz, 4H). **^{13}C NMR (100 MHz, CDCl$_3$)** δ (ppm): 130.2, 130.2, 129.0, 128.4, 57.3. **HRMS (MALDI)**: calcd for $C_{14}H_{15}OS$ $[M+H]^+$: 231.0838, found: 231.0838.

tert-Butyl methyl sulfoxide (**2o**)[14]

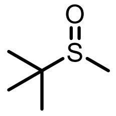

The title compound was prepared according to the general procedure on 1.0 mmol scale. Yellow oil (60.5 mg, 0.50 mmol, 50% yield). **^{1}H NMR (400 MHz, CDCl$_3$)** δ (ppm): 2.33 (s, 3H), 1.19 (s, 9H). **^{13}C NMR (100 MHz, CDCl$_3$)** δ (ppm): 52.8, 31.8, 22.4. **HRMS (MALDI)**: calcd for $C_5H_{11}OS$ $[M+H]^+$: 119.0525, found: 119.0520.

Dihexyl sulfoxide (**2p**)[15]

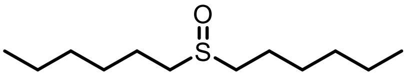

The title compound was prepared according to the general procedure. The mixture was purified by silica gel column chromatography (hexane/ethyl acetate = 1:1). Colorless oil (73.8 mg, 0.34 mmol, 67% yield). **^{1}H NMR (400 MHz, CDCl$_3$)** δ (ppm): 2.77–2.55 (m, 4H), 1.86–1.65 (m, 4H), 1.54–1.36 (m, 4H), 1.36–1.22 (m, 8H), 0.88 (t, *J* = 7.0 Hz, 6H). **^{13}C NMR (100 MHz, CDCl$_3$)** δ (ppm): 52.5, 31.4, 28.6, 22.7, 22.5, 14.1. **HRMS (MALDI)**: calcd for $C_{12}H_{27}OS$ $[M+H]^+$: 219.1777, found: 219.1779.

N-Acetylmethionine sulfoxide (**2q**)[16]

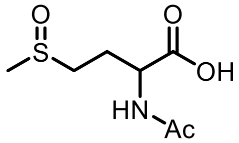

To a 50 mL screw-cap vial, *N*-Acetylmethionine (0.50 mmol), tetrabutylammonium chlorite (170 mg, 0.45 mmol), ethyl acetate (12 mL), and hydrogen chloride in ethyl acetate solution (350 μL, 0.35 mmol) were added, and the solution was stirred for 10 minutes at 25 °C. The reaction was concentrated under reduced pressure, and the mixture was purified by silica gel column chromatography (dichloromethane/Methanol = 3:1, 1 v/v% of acetic acid was added). Yellow liquid (87.2 mg, 0.42 mmol, 84% yield). **^{1}H NMR (400 MHz, DMSO-d$_6$)** δ (ppm): 8.43–8.14 (br, 1H), 4.45–4.14 (m, 1H), 2.87–2.57 (m, 2H), 2.56–2.51 (m, 3H), 2.15–2.00 (m, 1H), 1.98–1.75 (m, 2H), 1.85 (s, 3H). **^{13}C NMR (100 MHz, DMSO-d$_6$)** δ (ppm): 173.0, 172.0, 169.5, 169.4, 51.1, 50.9, 49.6, 49.5, 38.0, 37.9, 24.3, 24.0, 22.4. **HRMS (MALDI)**: calcd for $C_7H_{13}NO_4NaS$ $[M+Na]^+$: 230.0457, found: 230.0458.

4-(Phenylsulfinyl) benzaldehyde (**2r**)[17]

The title compound was prepared according to the general procedure. The mixture was purified by silica gel column chromatography (hexane/ethyl acetate = 3:1). White solid (81.0 mg, 0.36 mmol, 71%

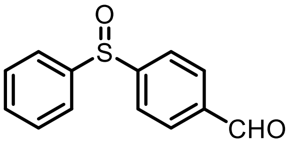

yield). **^{1}H NMR (400 MHz, CDCl$_3$)** δ (ppm): 10.02 (s, 1H), 7.95 (d, *J* = 8.8 Hz, 2H), 7.81 (d, *J* = 8.0 Hz, 2H), 7.71–7.63 (m, 2H), 7.53–7.41 (m, 3H). **^{13}C NMR (100 MHz, CDCl$_3$)** δ (ppm): 191.2, 152.2, 144.9, 138.0, 131.8, 130.5, 129.7, 125.0, 125.0. **HRMS(MALDI)**: calcd for $C_{13}H_{11}O_2S$ $[M+H]^+$: 231.0474, found: 231.0480.

2-(4-(phenylsulfinyl)phenyl)-1,3-dioxolane (**2s**)

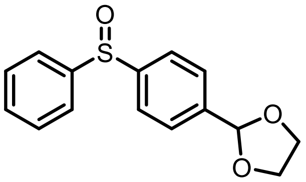

The title compound was prepared according to the general procedure with 0.9 equivalents of hydrogen chloride on 0.40 mmol scale. The mixture was purified by silica gel column chromatography (hexane/ethyl acetate = 3:1). Yellow solid (102 mg, 0.37 mmol, 93% yield). **Mp**: 67.2–67.6°C **^{1}H NMR (400 MHz, CDCl$_3$)** δ (ppm): 7.73–7.60 (m, 4H), 7.57 (d, *J* = 7.6 Hz, 2H), 7.48–7.40 (m, 3H), 5.79 (s, 1H), 4.14–3.98 (m, 4H). **^{13}C NMR (100 MHz, CDCl$_3$)** δ (ppm): 146.5, 145.5, 141.1, 131.2, 129.4, 127.5, 124.9, 124.8, 102.9, 65.5. **HRMS (MALDI)**: calcd for $C_{15}H_{15}O_3S$ $[M+H]^+$: 275.0736, found: 275.0738.

Diphenyl sulfone (**3a**)[18]

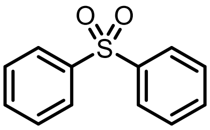

The title compound was prepared according to the general procedure. White solid (41.4 mg, 0.19 mmol, 95% yield). **^{1}H NMR (400 MHz, CDCl$_3$)** δ (ppm): 8.04–7.88 (m, 4H), 7.65–7.43 (m, 6H). **^{13}C NMR (100 MHz, CDCl$_3$)** δ (ppm): 144.4, 132.8, 126.2, 126.0. **HRMS(MALDI)**: calcd for $C_{12}H_{11}O_2S$ $[M+H]^+$: 219.0474, found: 219.0476.

9. NMR spectra

^{1}H NMR (400 MHz, $CDCl_3$, 25 °C) of **1e**

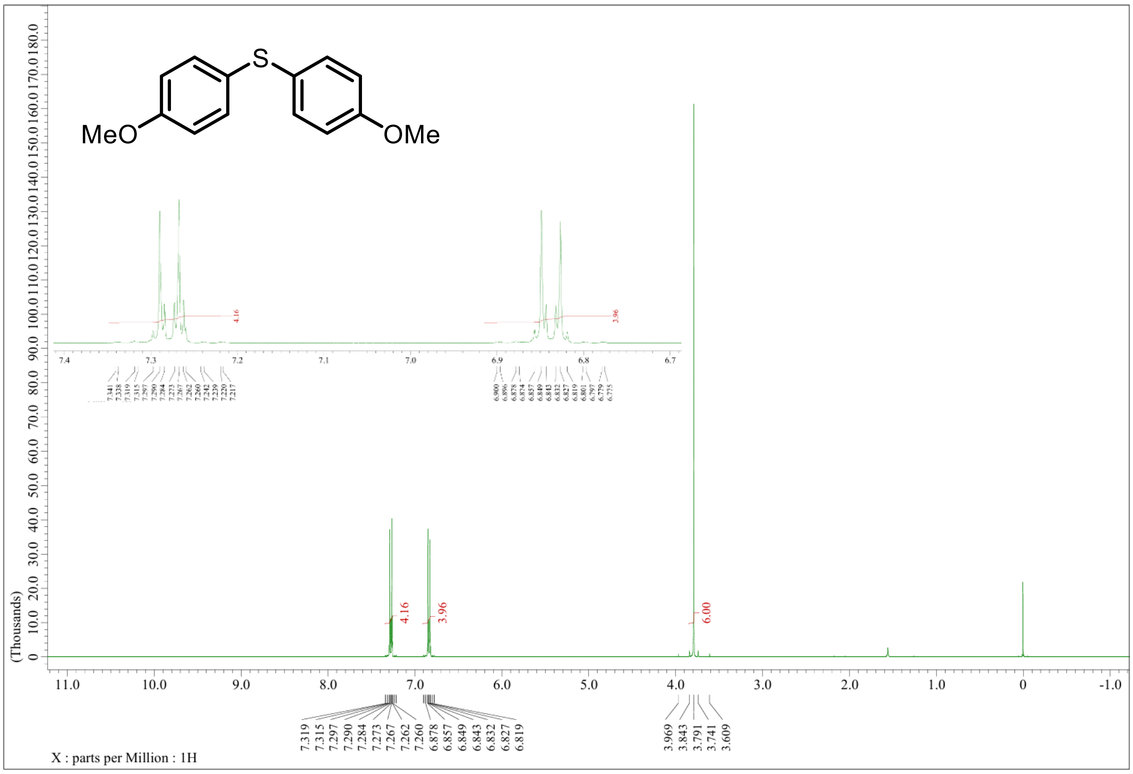

^{13}C NMR (100 MHz, $CDCl_3$, 25 °C) of **1e**

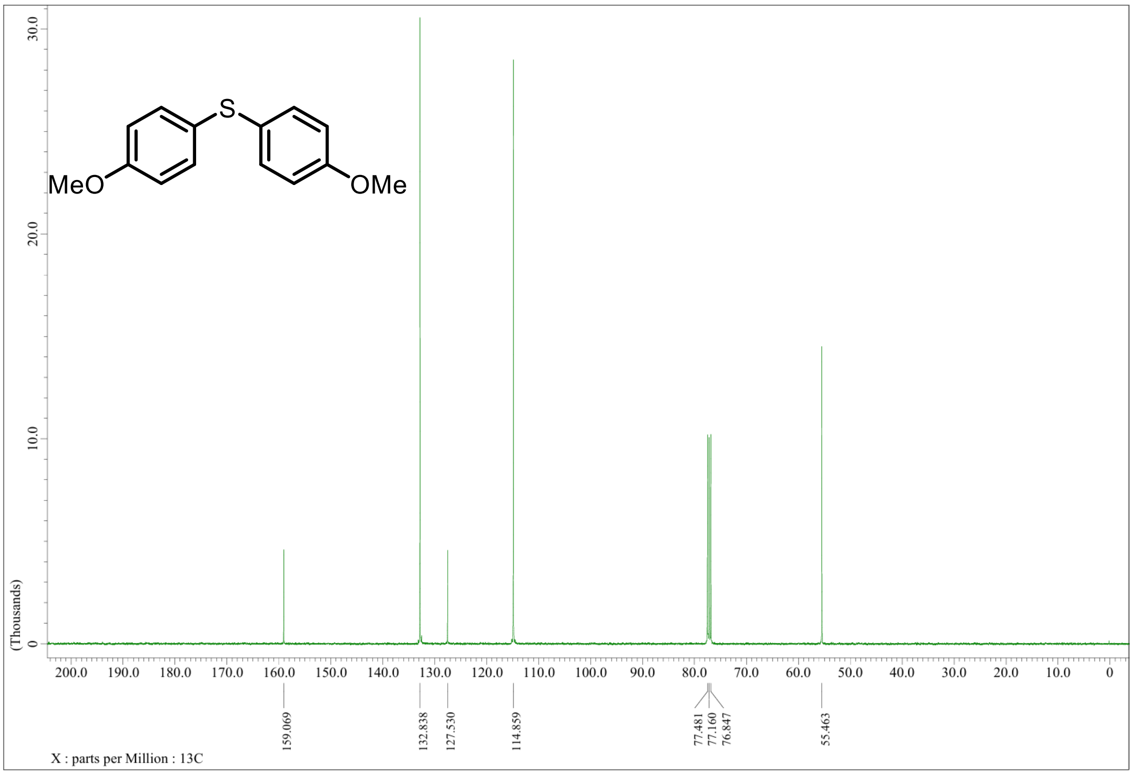

^{1}H NMR (400 MHz, $CDCl_3$, 25 °C) of **1s**

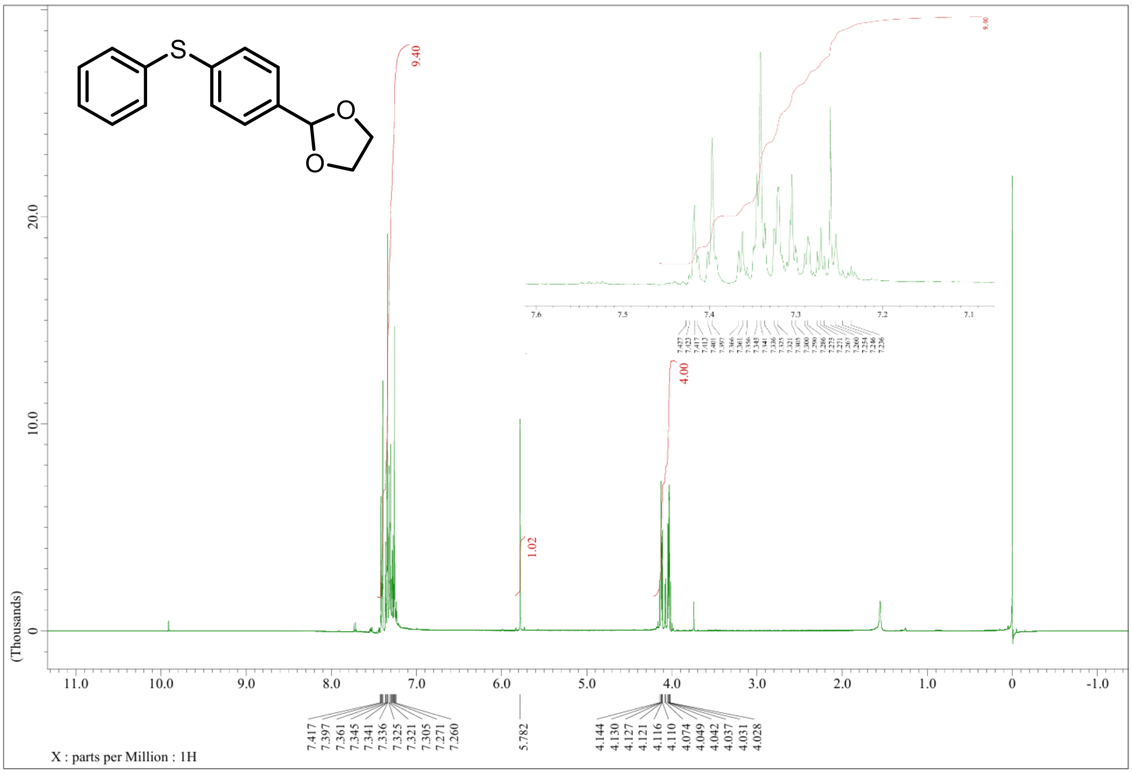

^{13}C NMR (100 MHz, $CDCl_3$, 25 °C) of **1s**

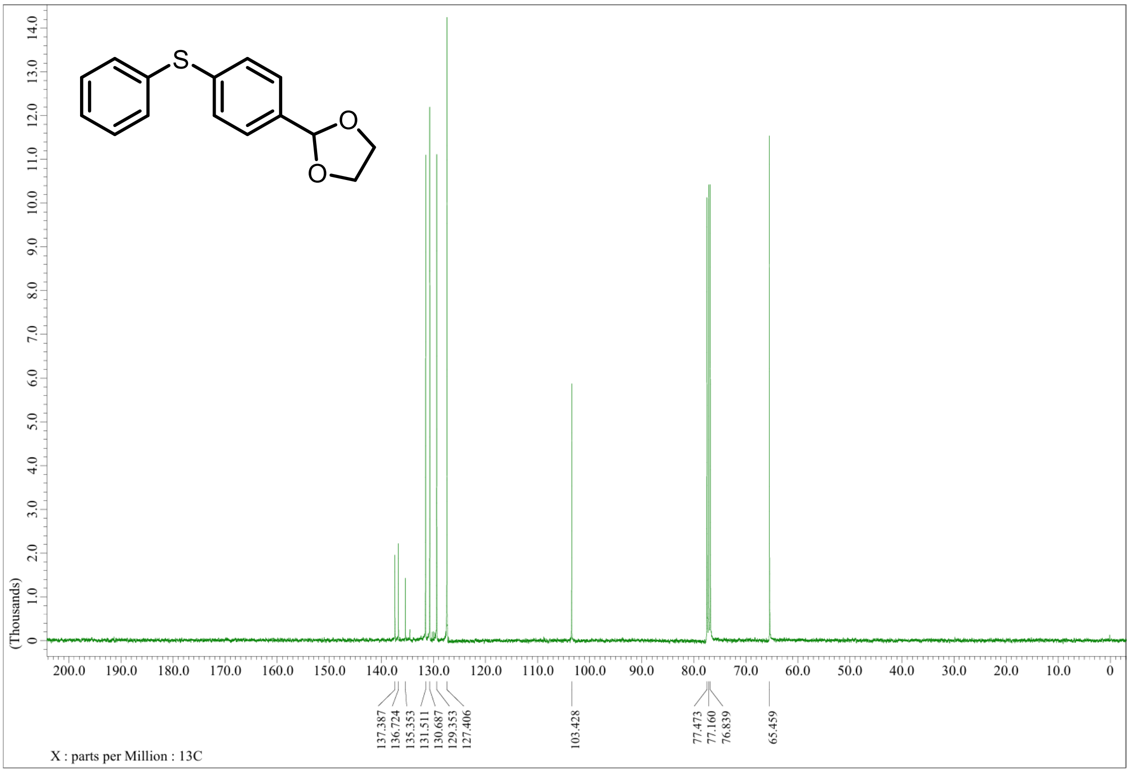

^{1}H NMR (400 MHz, $CDCl_3$, 25 °C) of **2a**

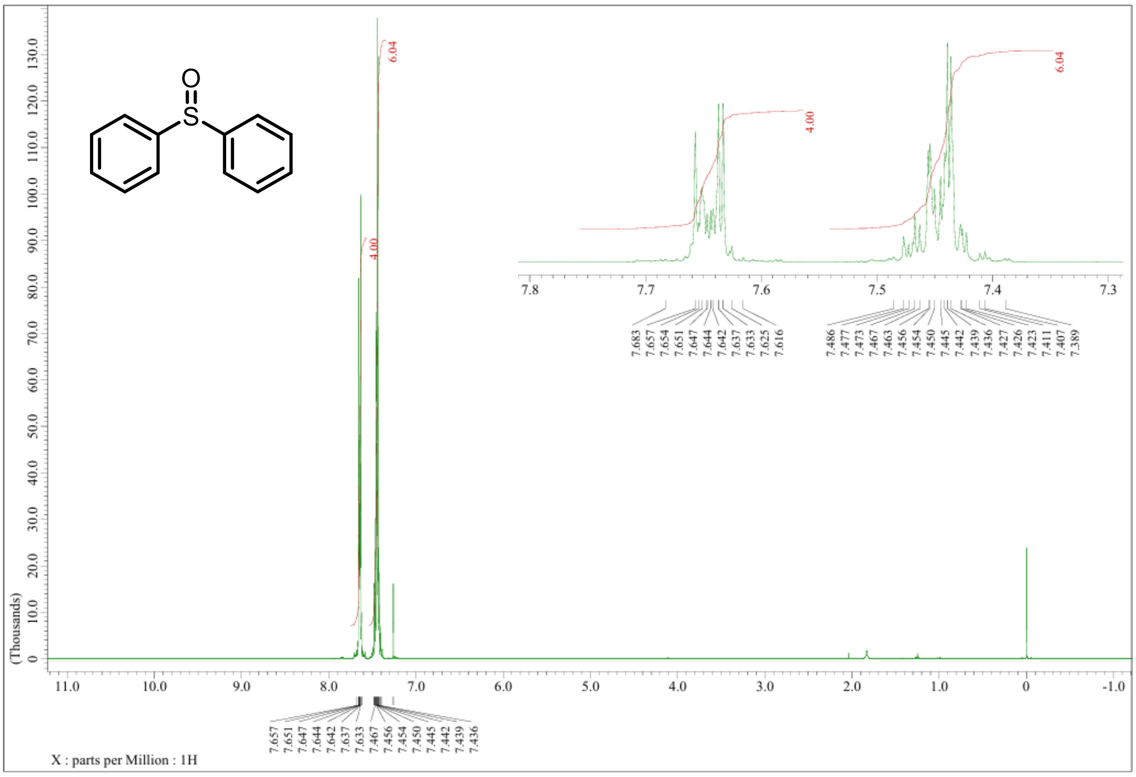

^{13}C NMR (100 MHz, $CDCl_3$, 25 °C) of **2a**

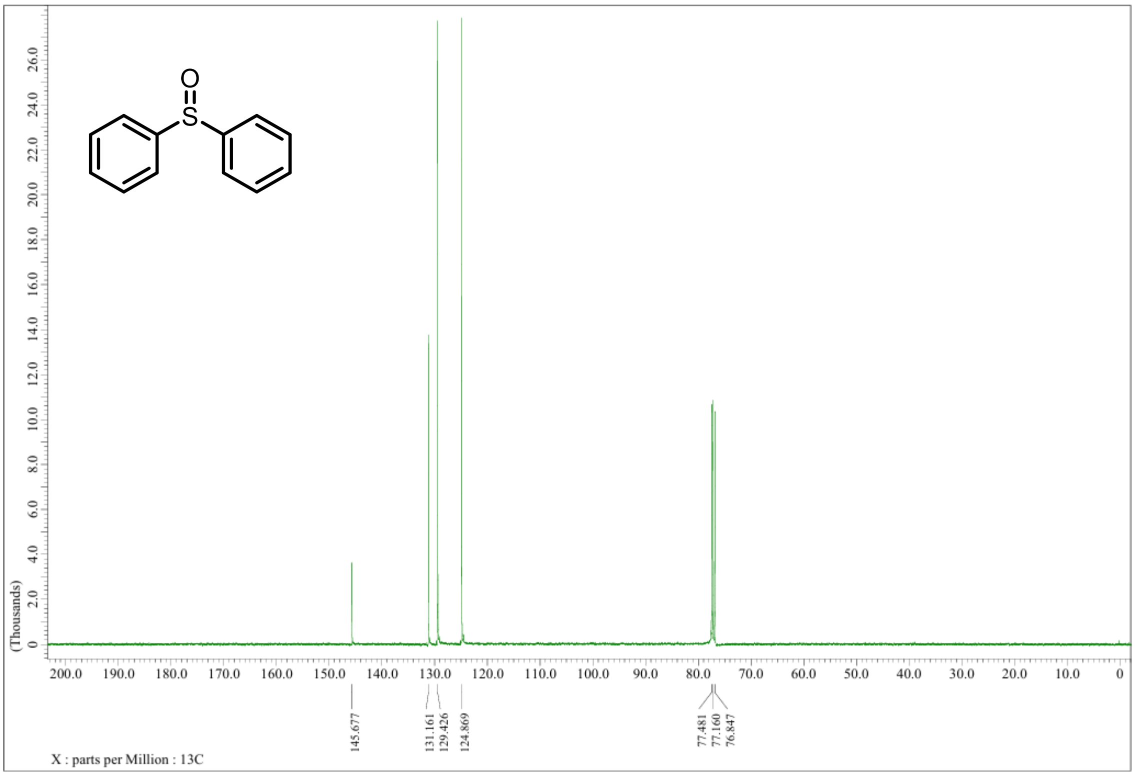

^{1}H NMR (400 MHz, $CDCl_3$, 25 °C) of **2b**

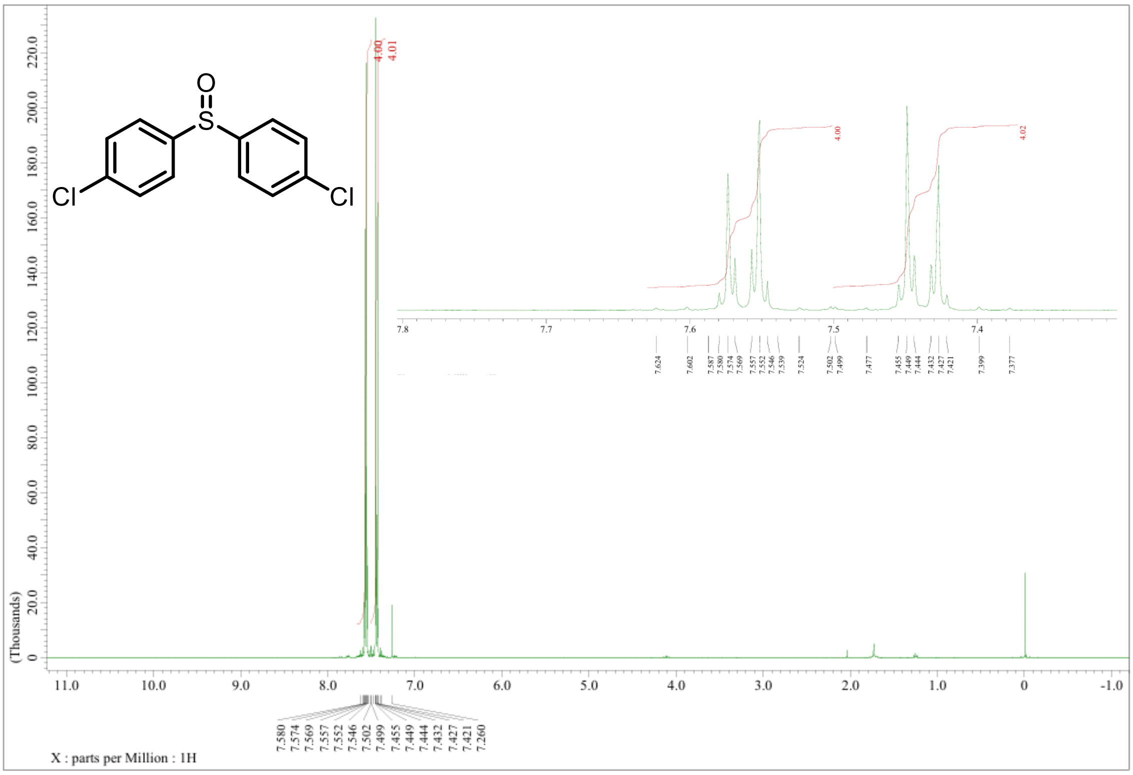

^{13}C NMR (100 MHz, $CDCl_3$, 25 °C) of **2b**

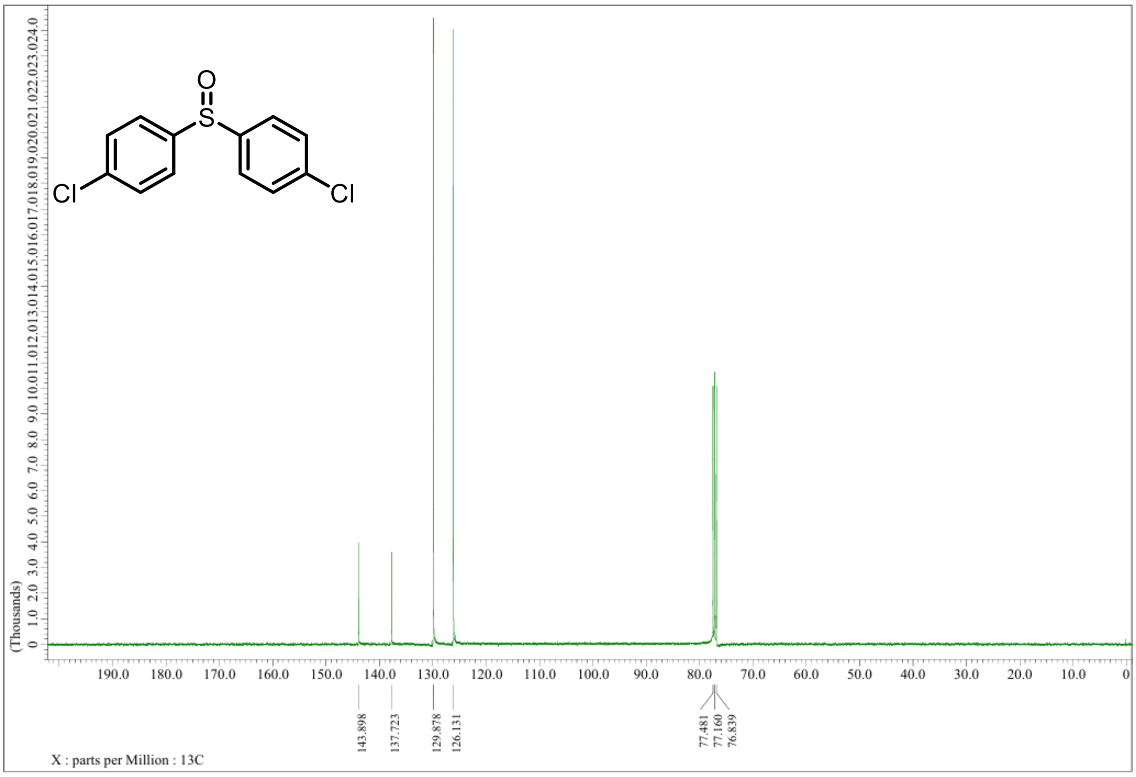

^{1}H NMR (400 MHz, $CDCl_3$, 25 °C) of **2c**

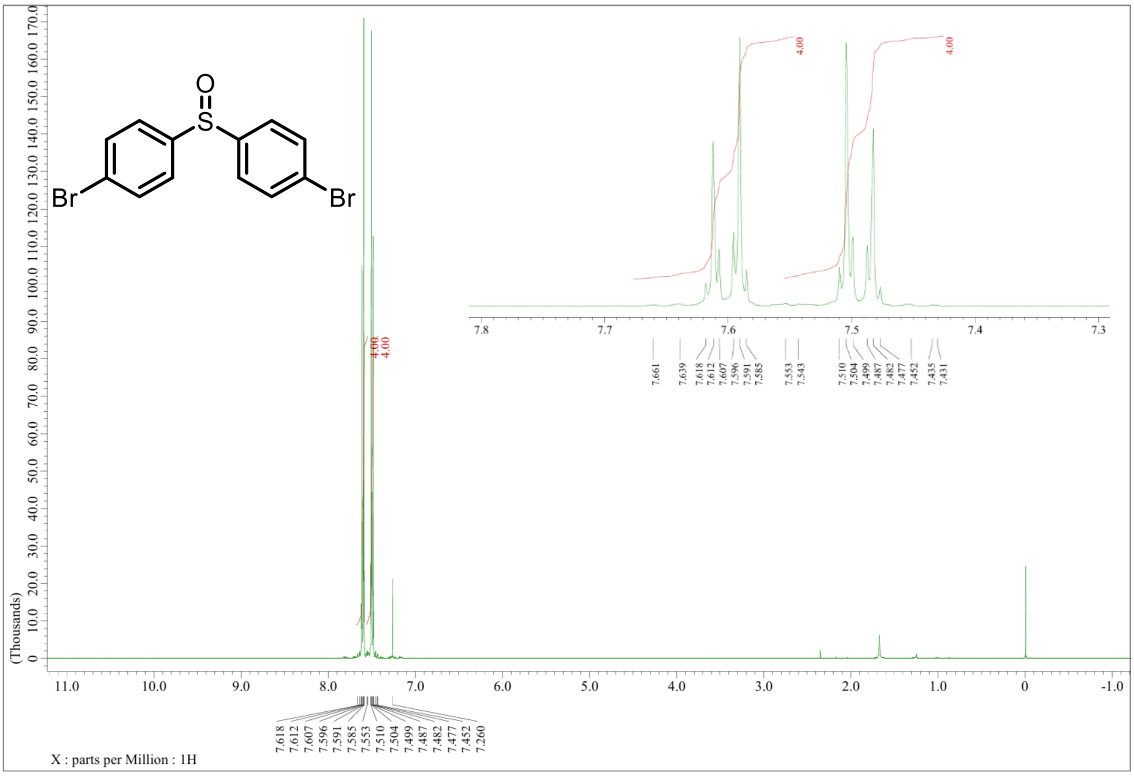

^{13}C NMR (100 MHz, $CDCl_3$, 25 °C) of **2c**

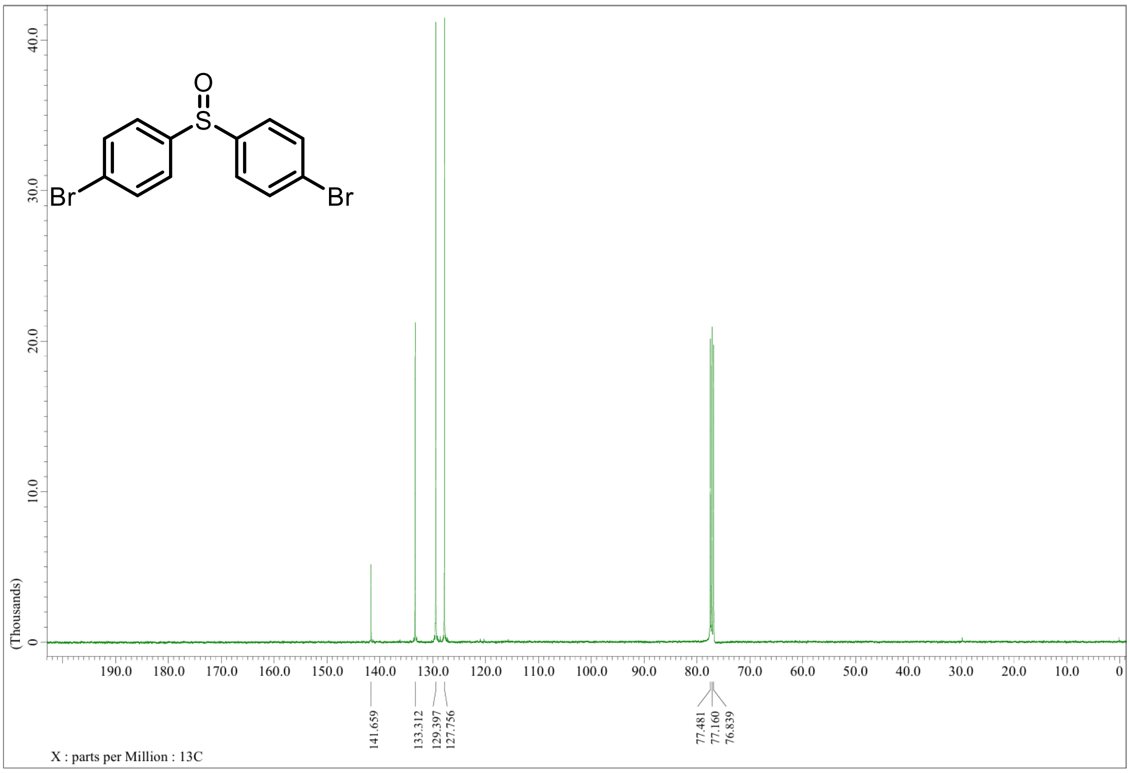

^{1}H NMR (400 MHz, $CDCl_3$, 25 °C) of **2d**

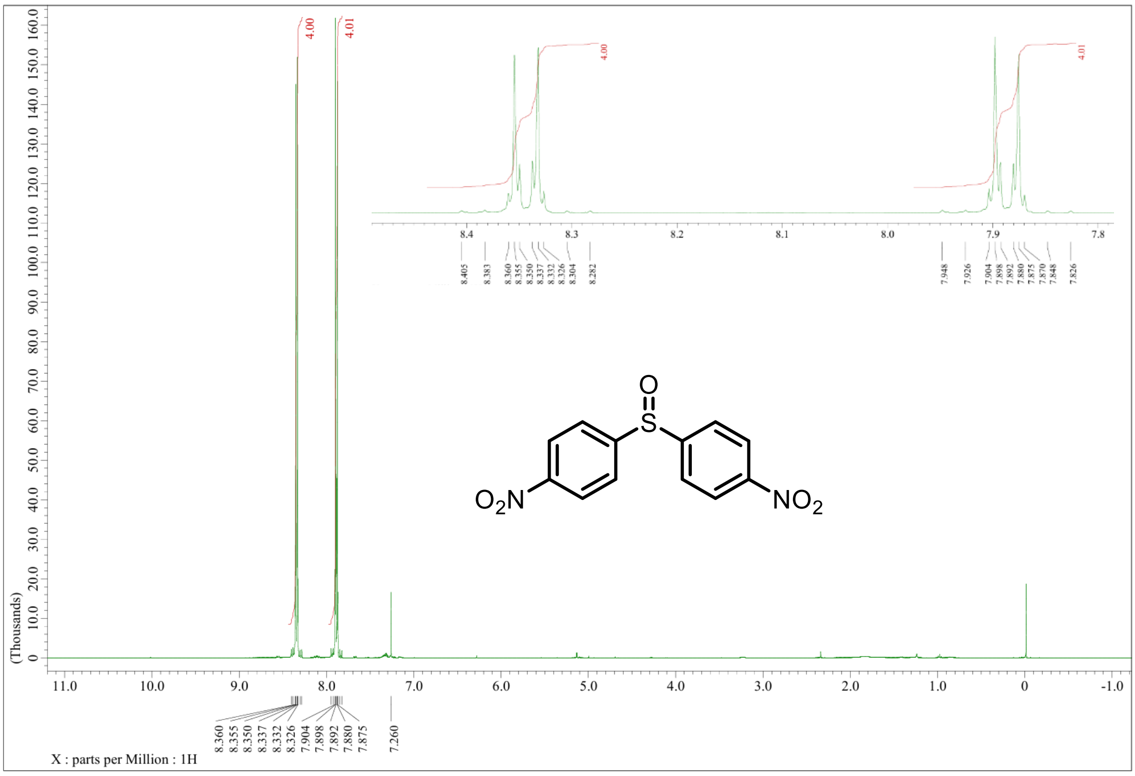

^{13}C NMR (100 MHz, $CDCl_3$, 25 °C) of **2d**

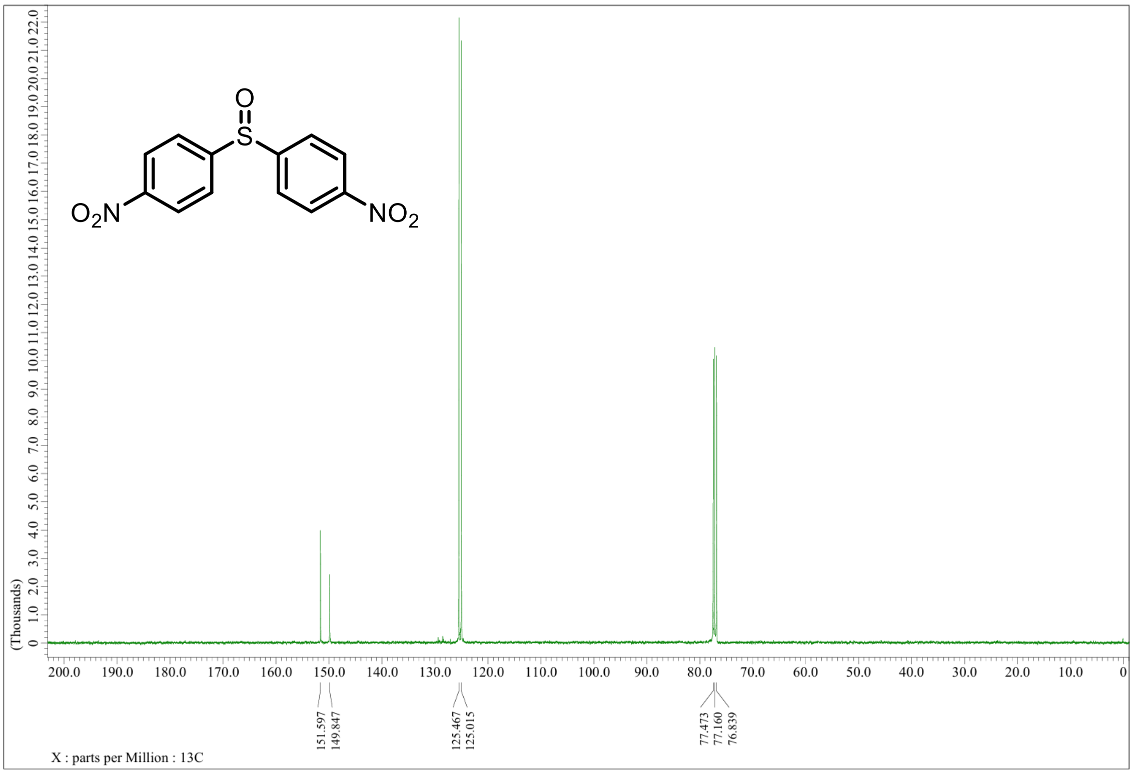

^{1}H NMR (400 MHz, $CDCl_3$, 25 °C) of **2e**

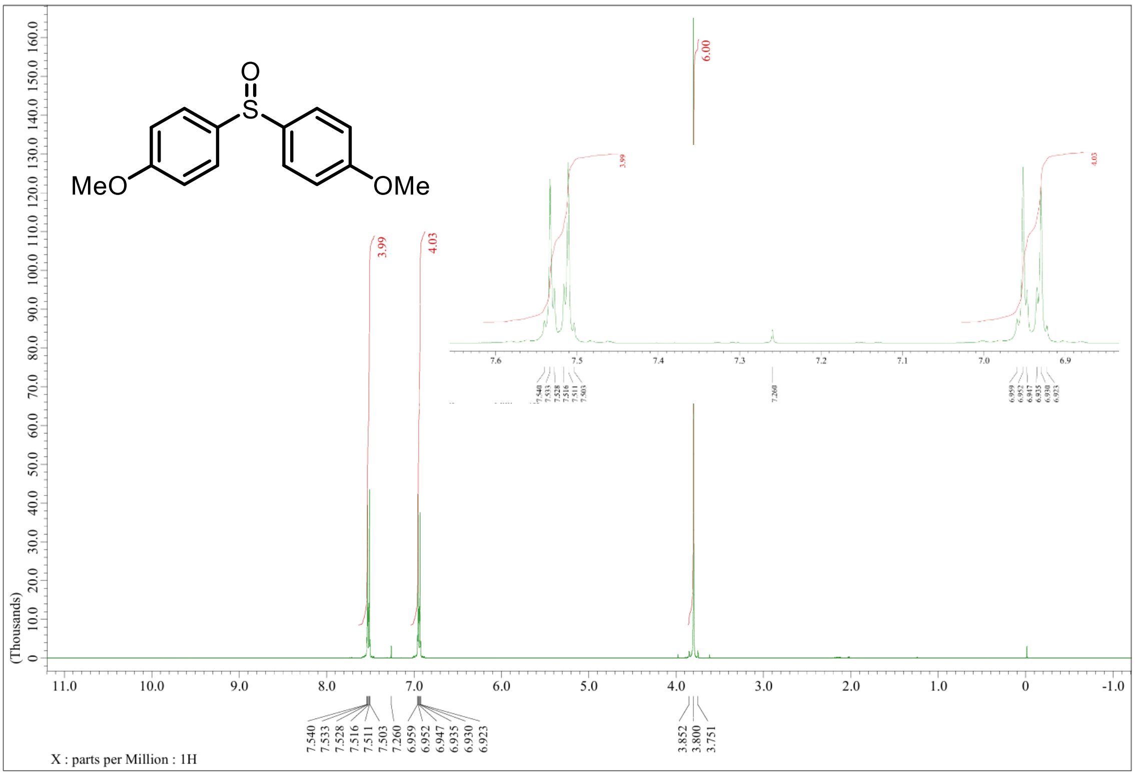

^{13}C NMR (100 MHz, $CDCl_3$, 25 °C) of **2e**

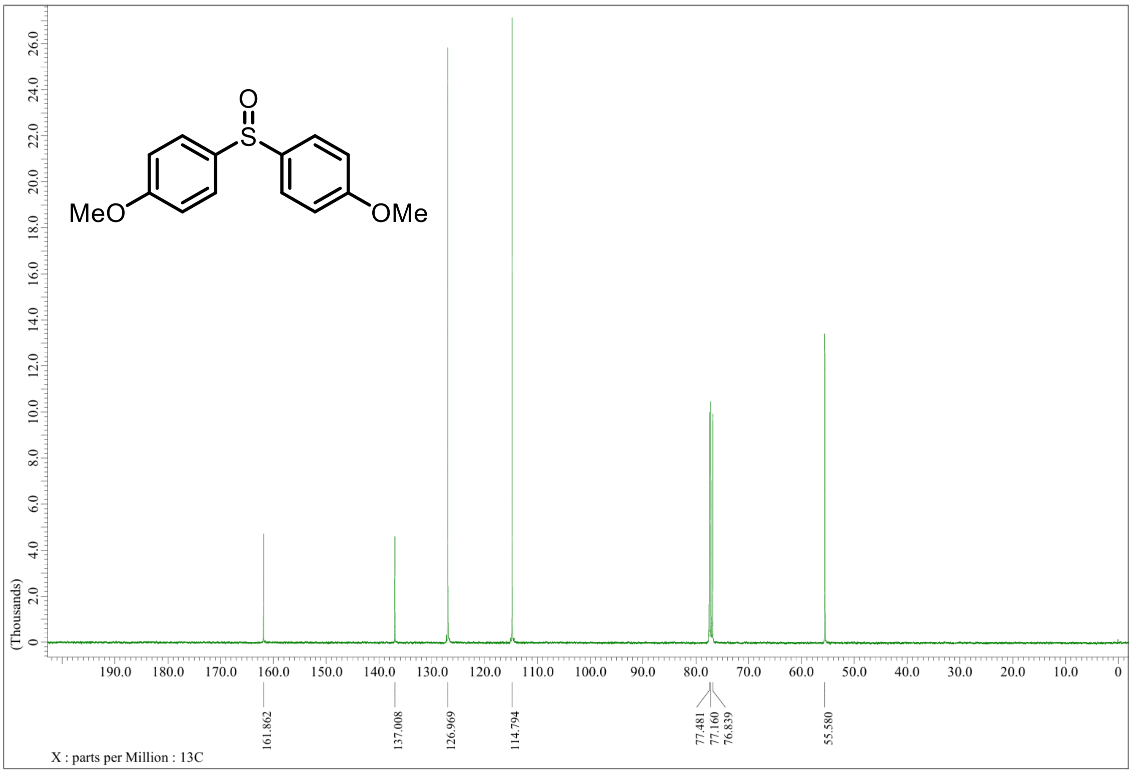

^{1}H NMR (400 MHz, $CDCl_3$, 25 °C) of **2f**

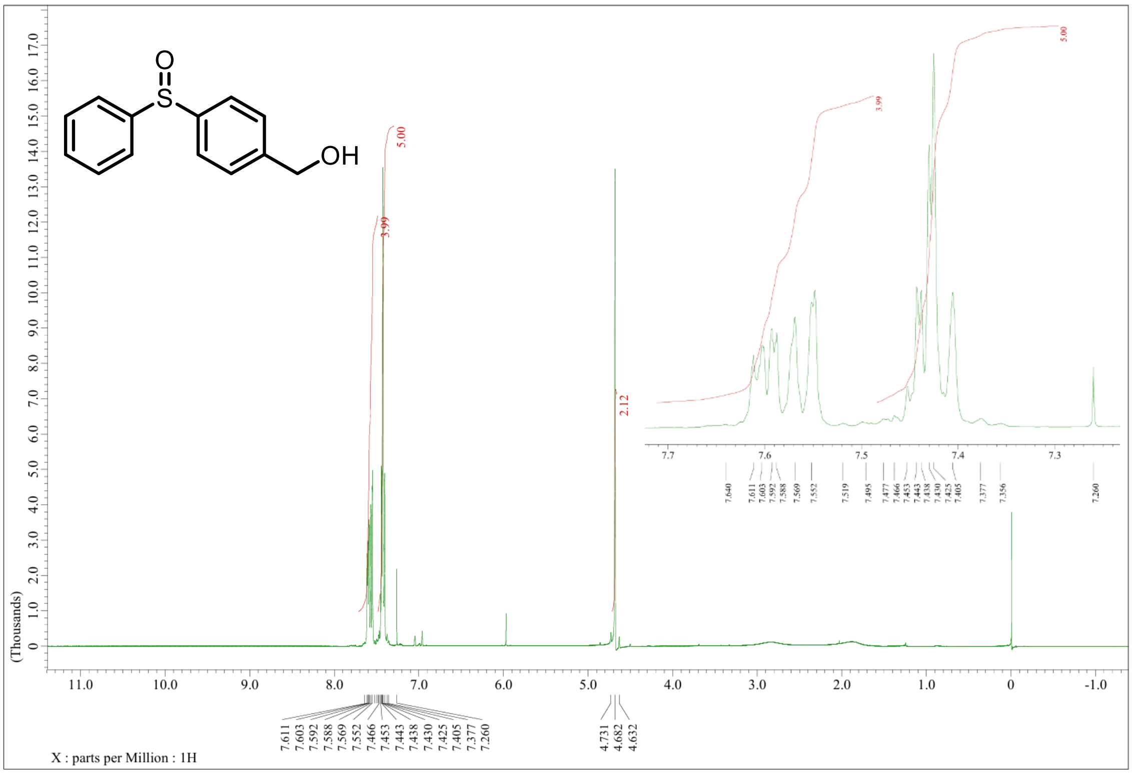

^{13}C NMR (100 MHz, $CDCl_3$, 25 °C) of **2f**

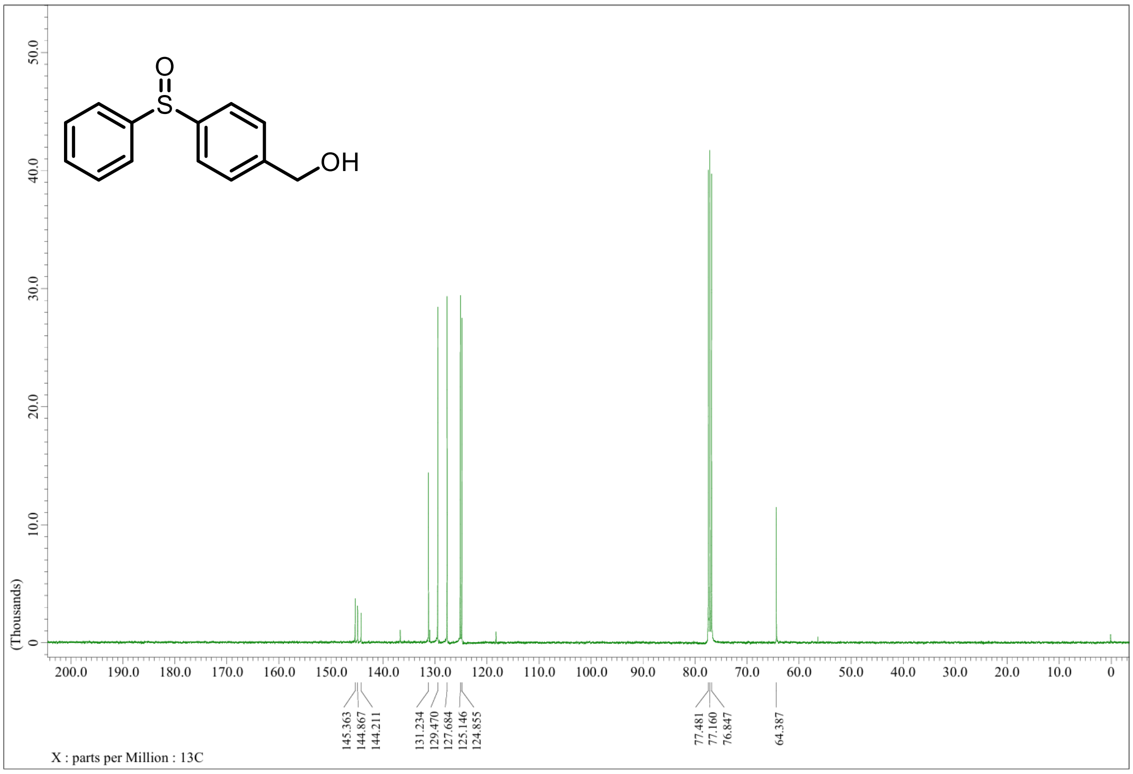

^{1}H NMR (400 MHz, $CDCl_3$, 25 °C) of **2g**

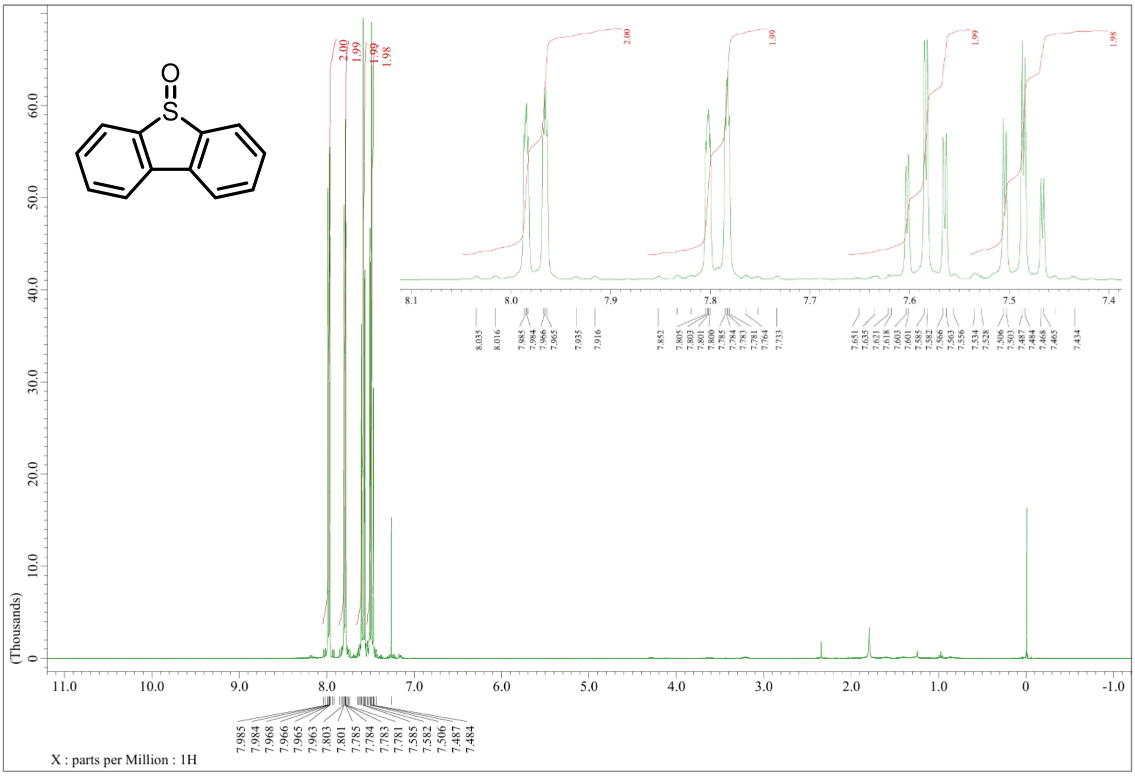

^{13}C NMR (100 MHz, $CDCl_3$, 25 °C) of **2g**

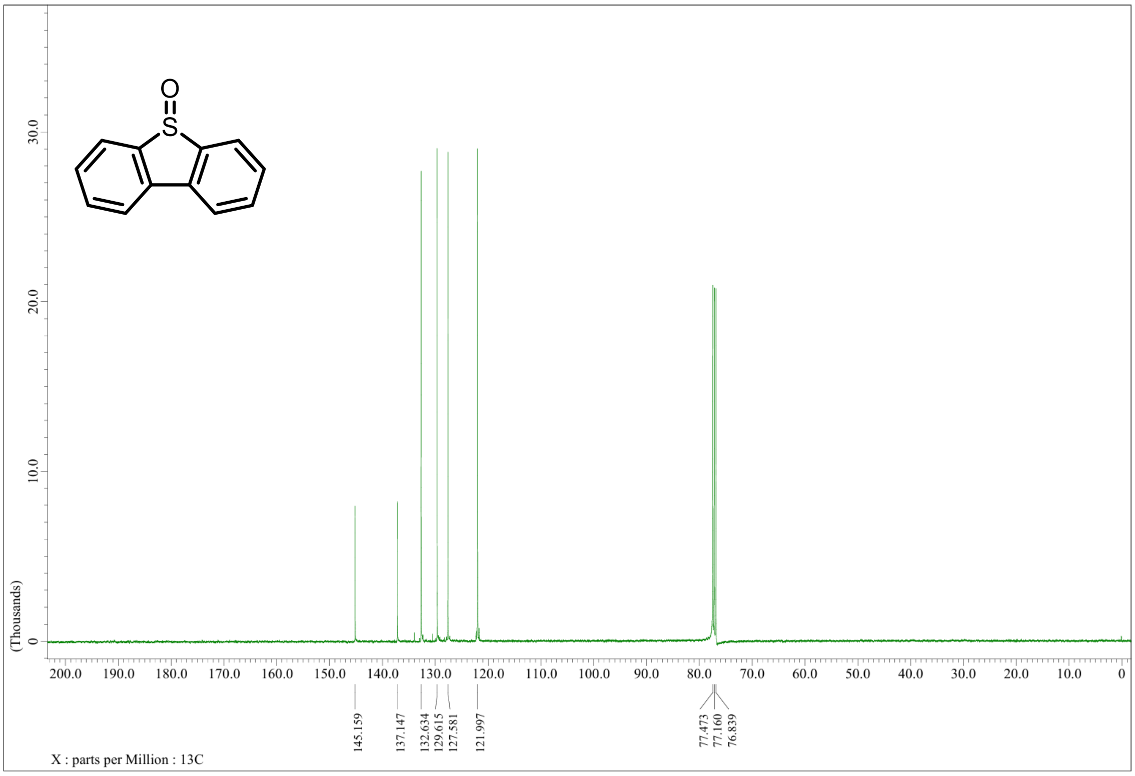

^{1}H NMR (400 MHz, $CDCl_3$, 25 °C) of **2h**

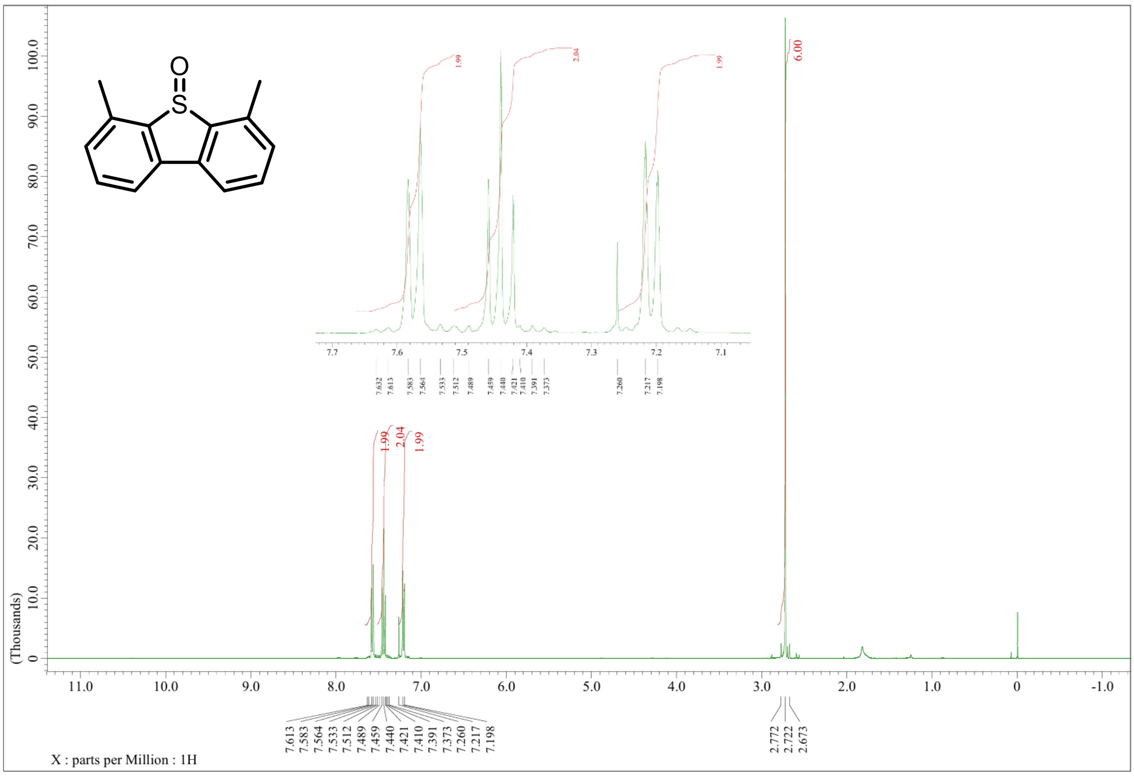

^{13}C NMR (100 MHz, $CDCl_3$, 25 °C) of **2h**

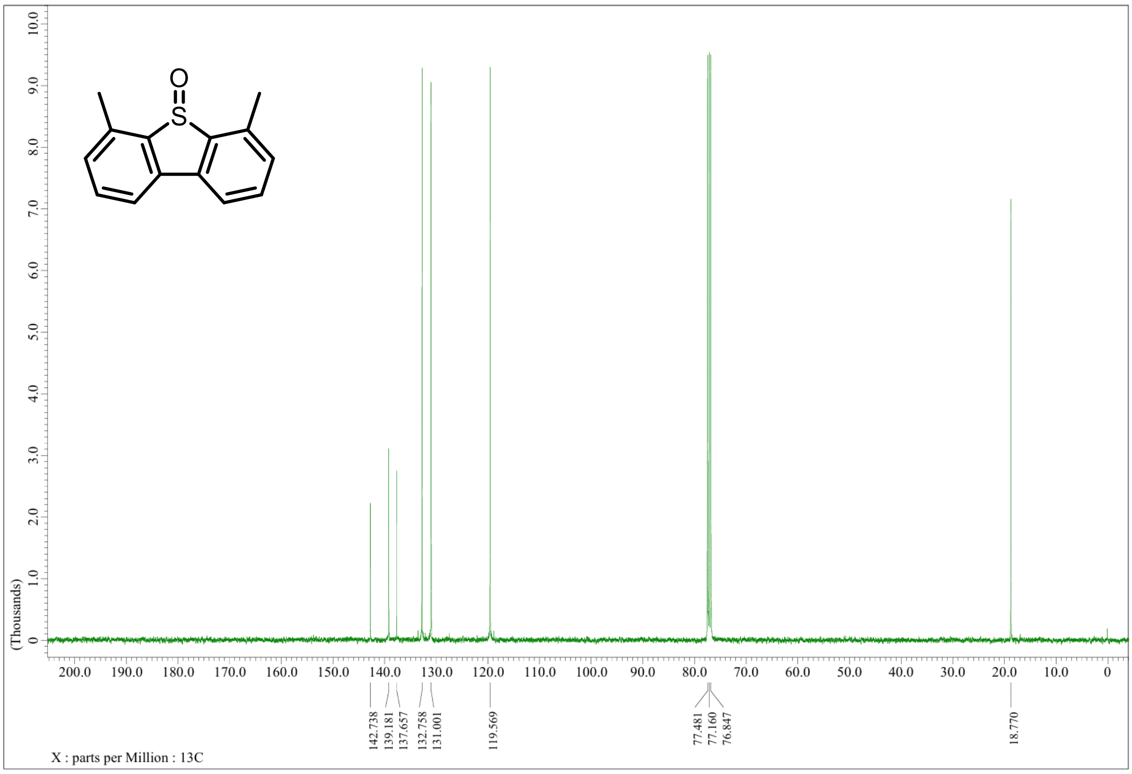

^{1}H NMR (400 MHz, $CDCl_3$, 25 °C) of **2i**

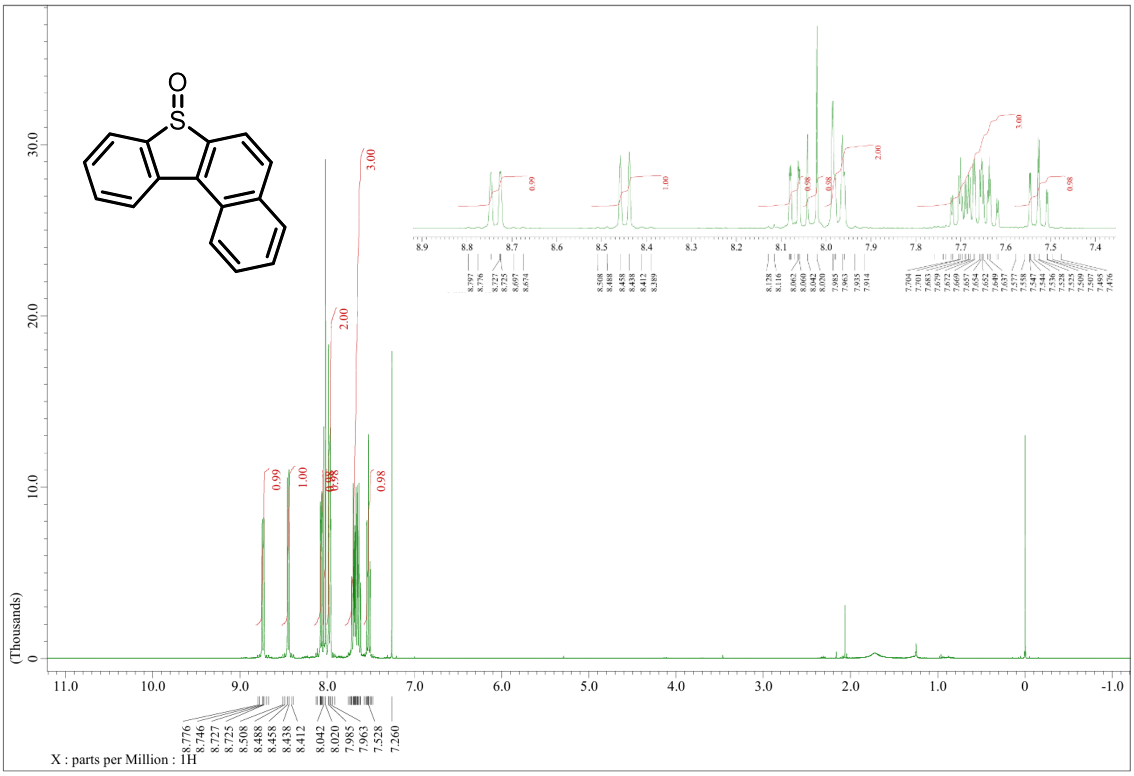

^{13}C NMR (100 MHz, $CDCl_3$, 25 °C) of **2i**

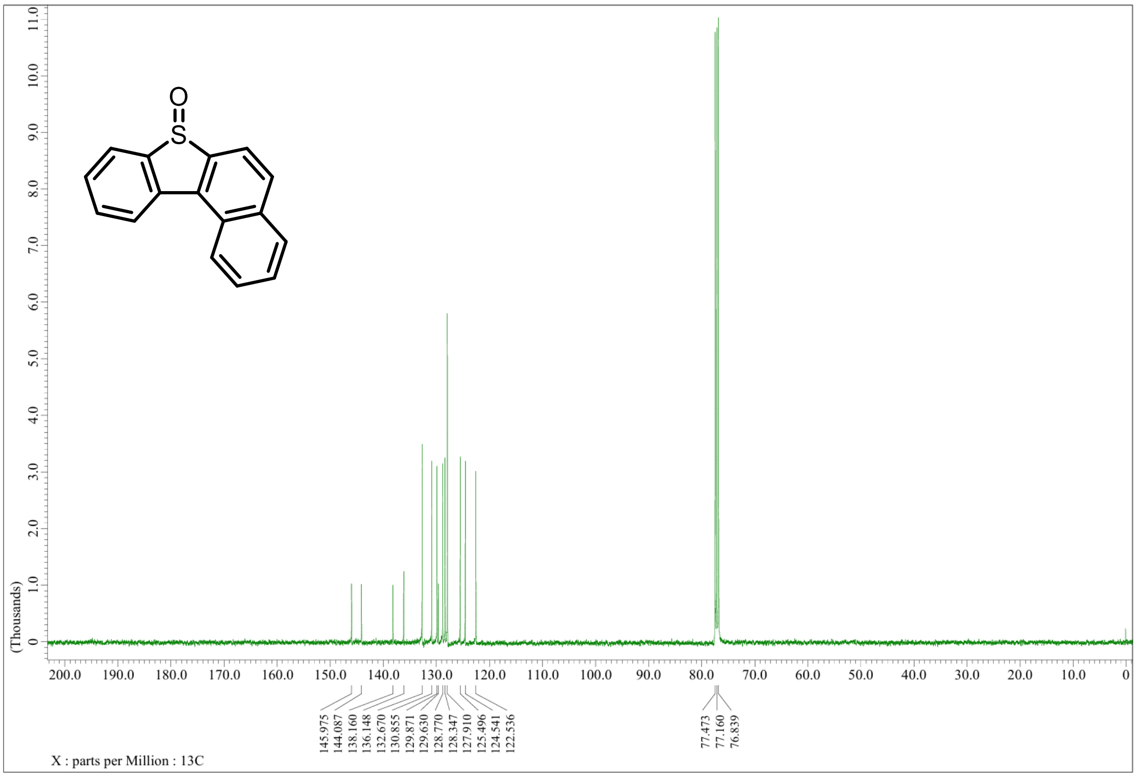

^{1}H NMR (400 MHz, $CDCl_3$, 25 °C) of **2j**

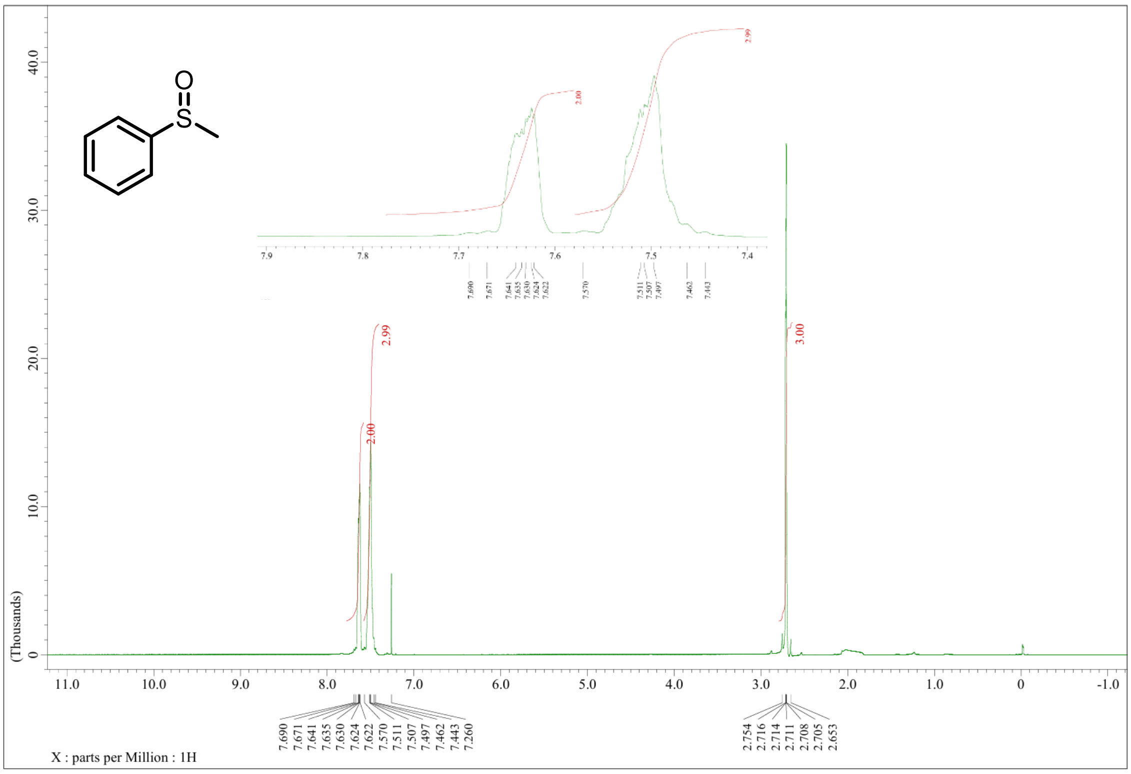

^{13}C NMR (100 MHz, $CDCl_3$, 25 °C) of **2j**

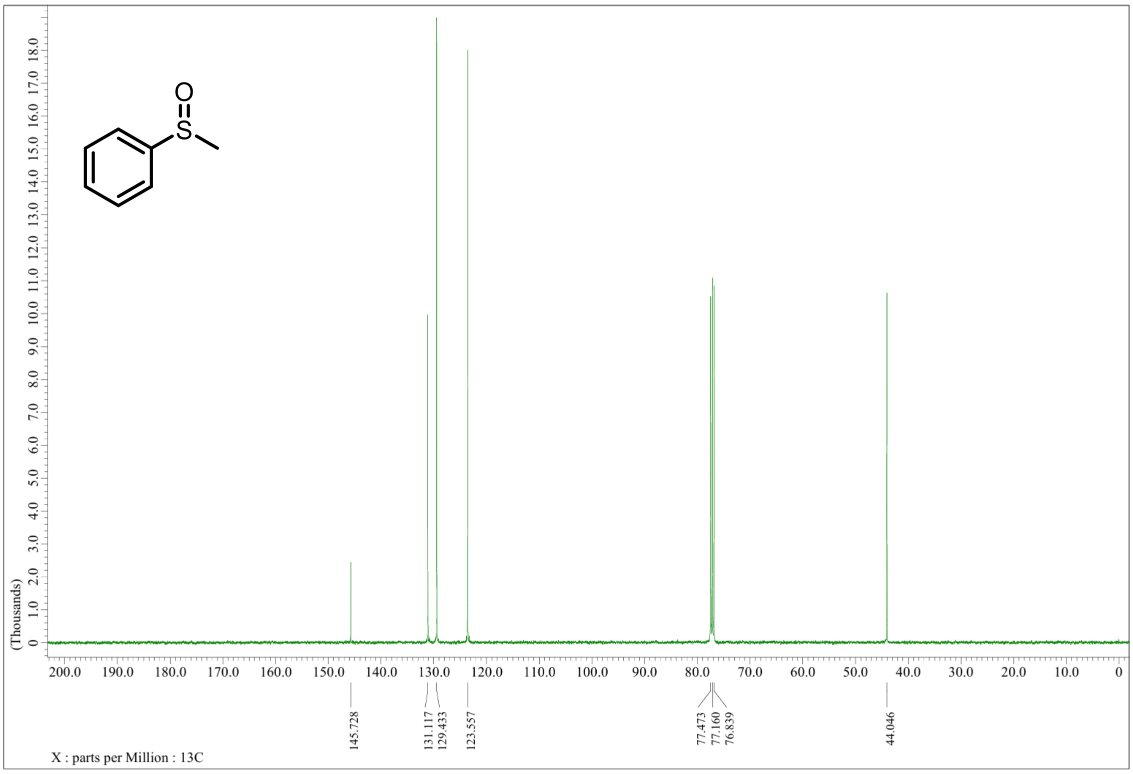

^{1}H NMR (400 MHz, CDCl$_3$, 25 °C) of **2k**

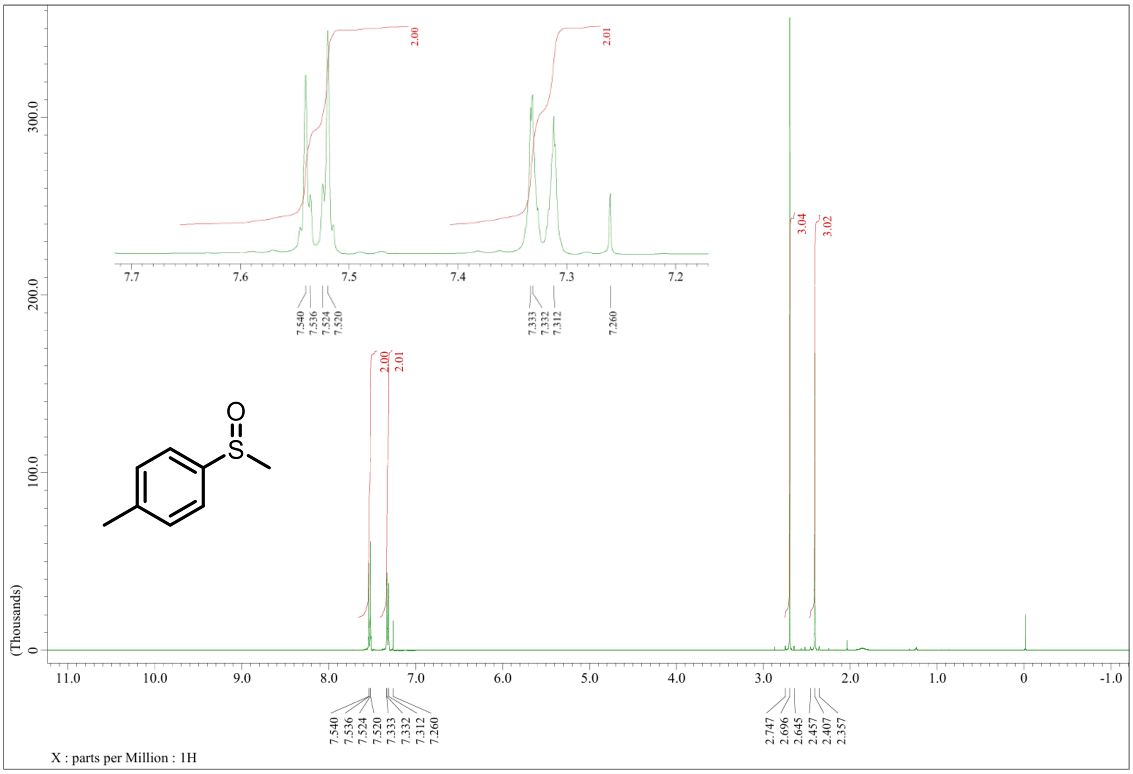

^{13}C NMR (100 MHz, CDCl$_3$, 25 °C) of **2k**

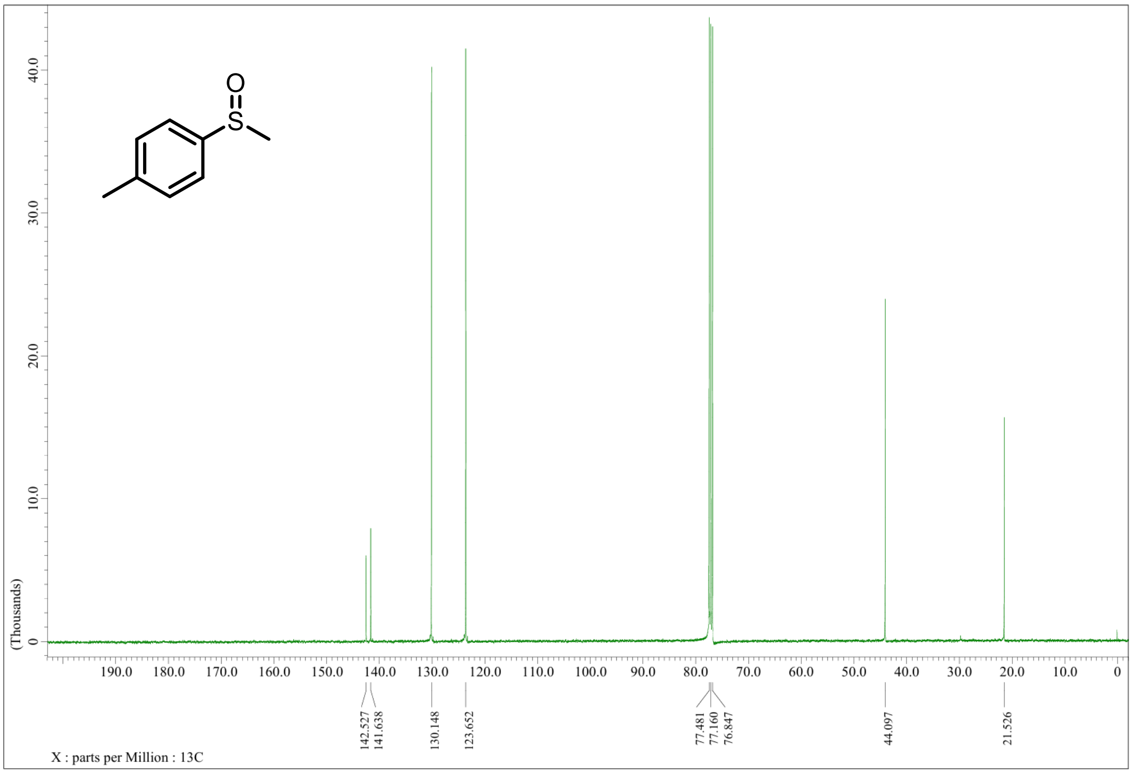

^{1}H NMR (400 MHz, $CDCl_3$, 25 °C) of **2l**

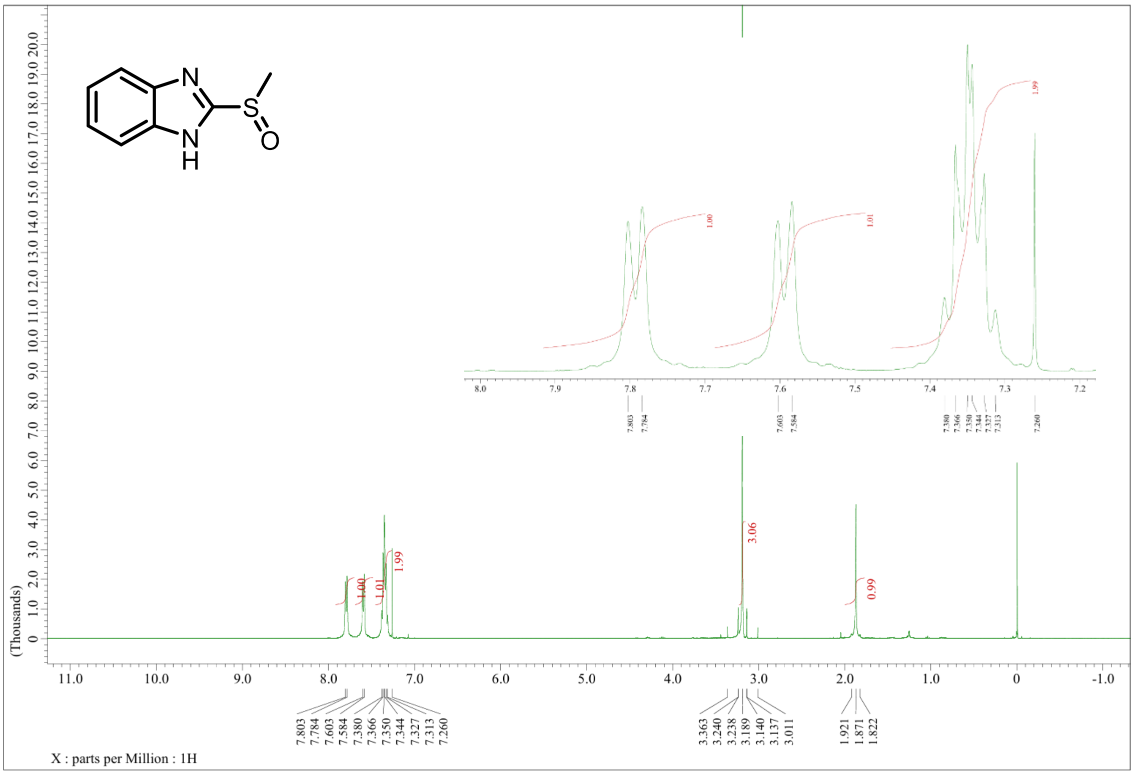

^{13}C NMR (100 MHz, $CDCl_3$, 25 °C) of **2l**

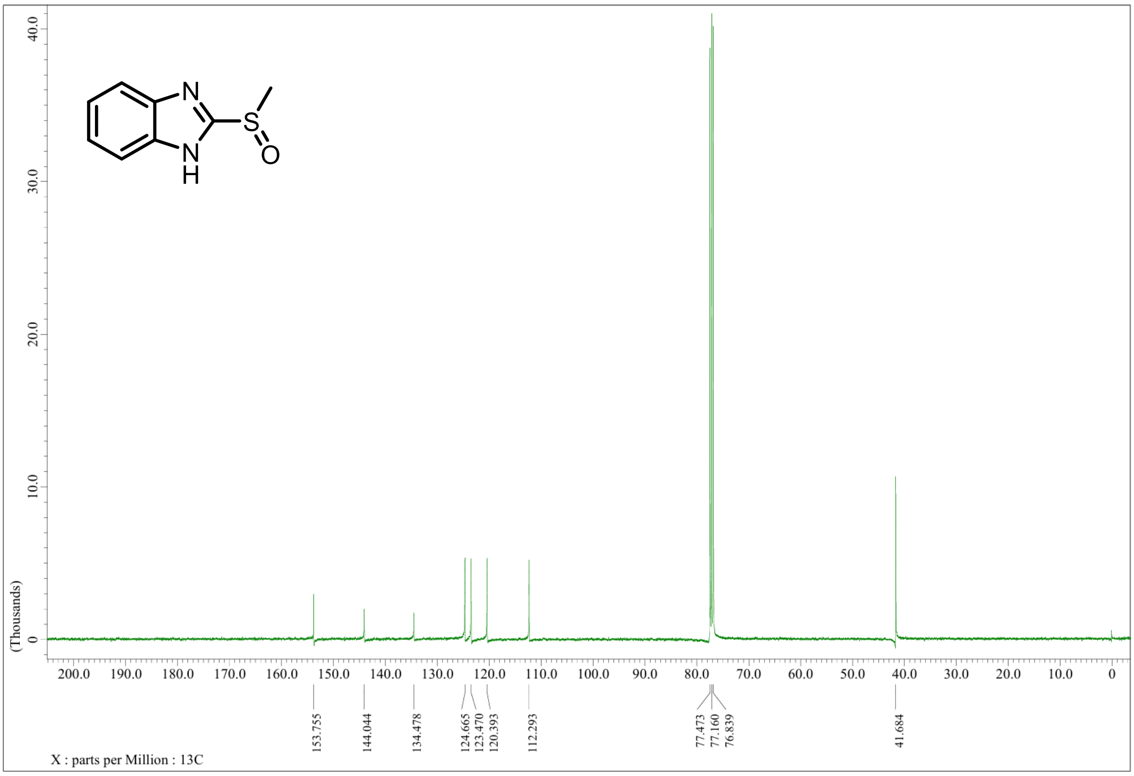

^{1}H NMR (400 MHz, $CDCl_3$, 25 °C) of **2m**

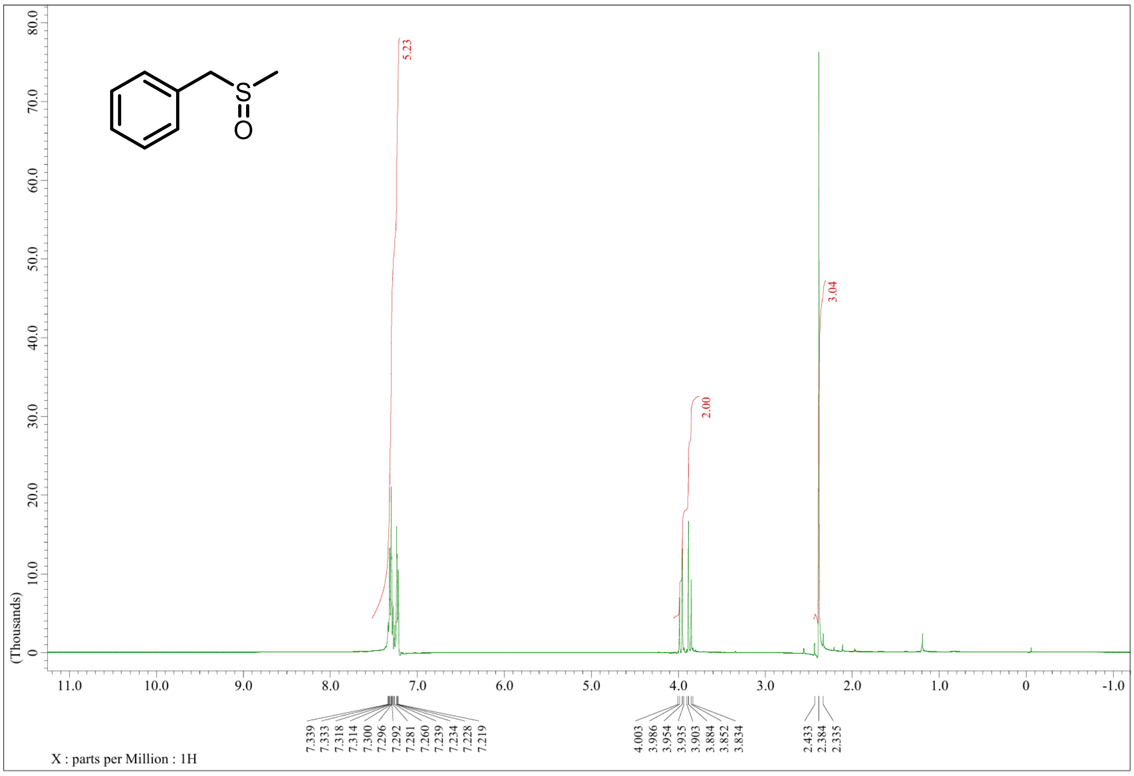

^{13}C NMR (100 MHz, $CDCl_3$, 25 °C) of **2m**

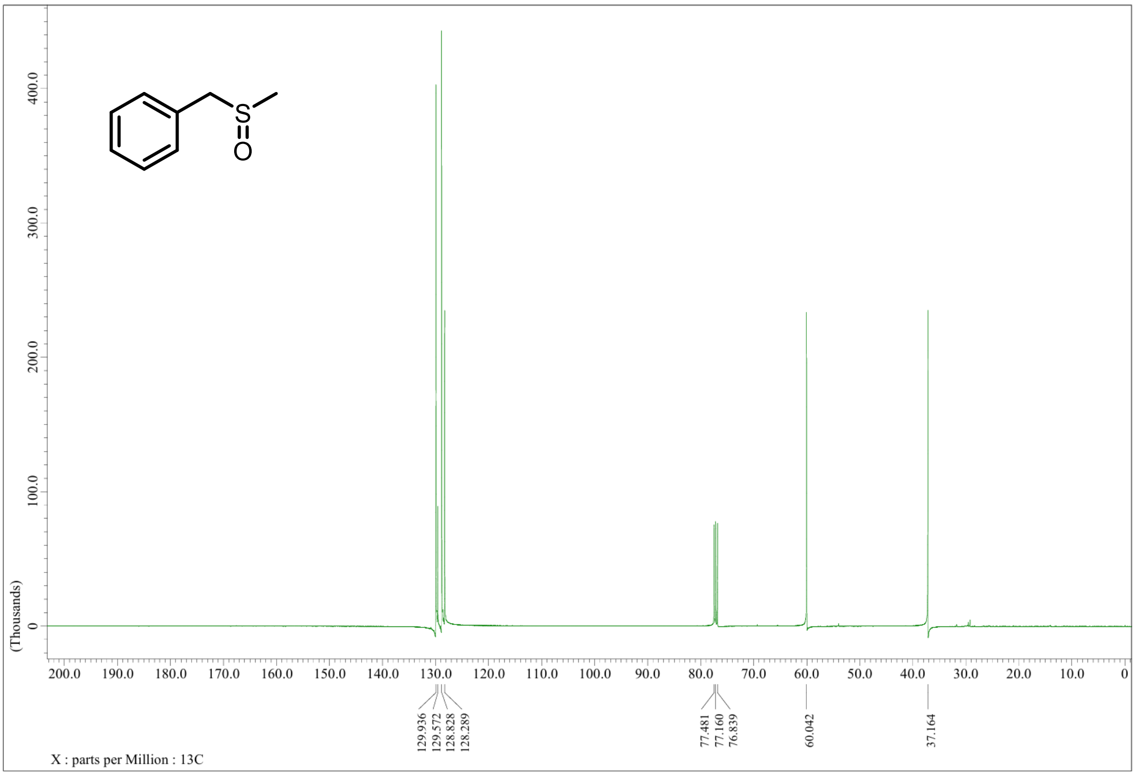

^{1}H NMR (400 MHz, $CDCl_3$, 25 °C) of **2n**

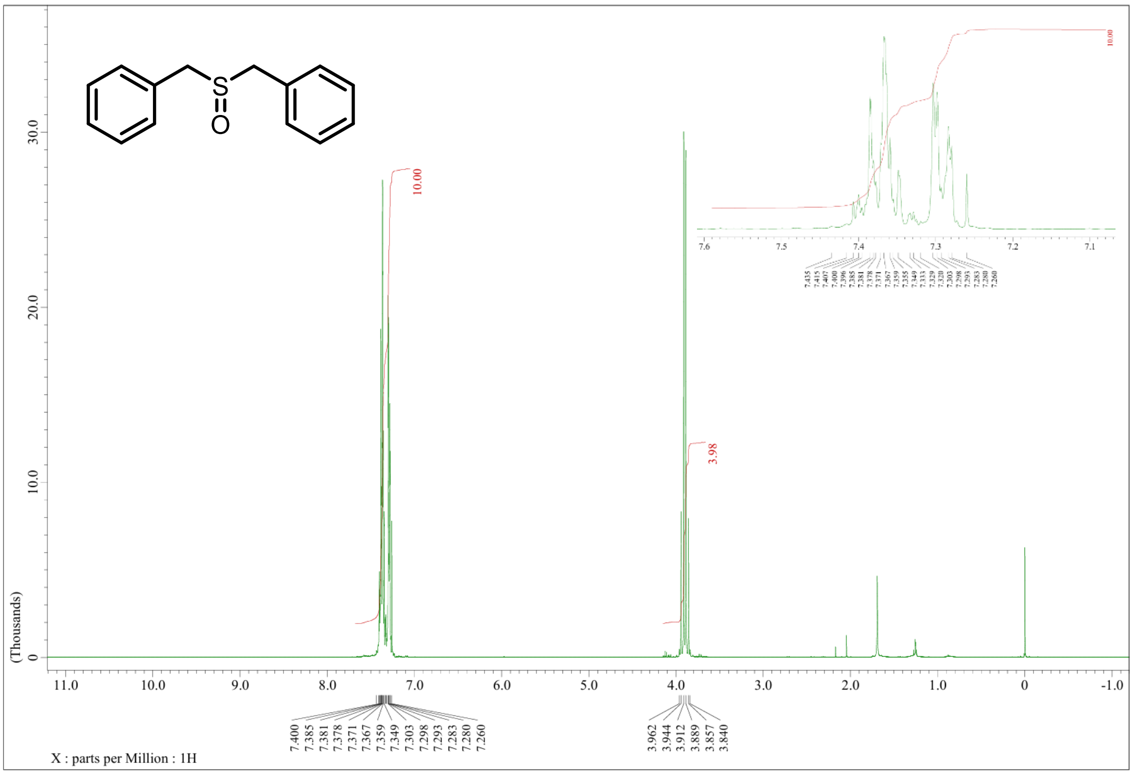

^{13}C NMR (100 MHz, $CDCl_3$, 25 °C) of **2n**

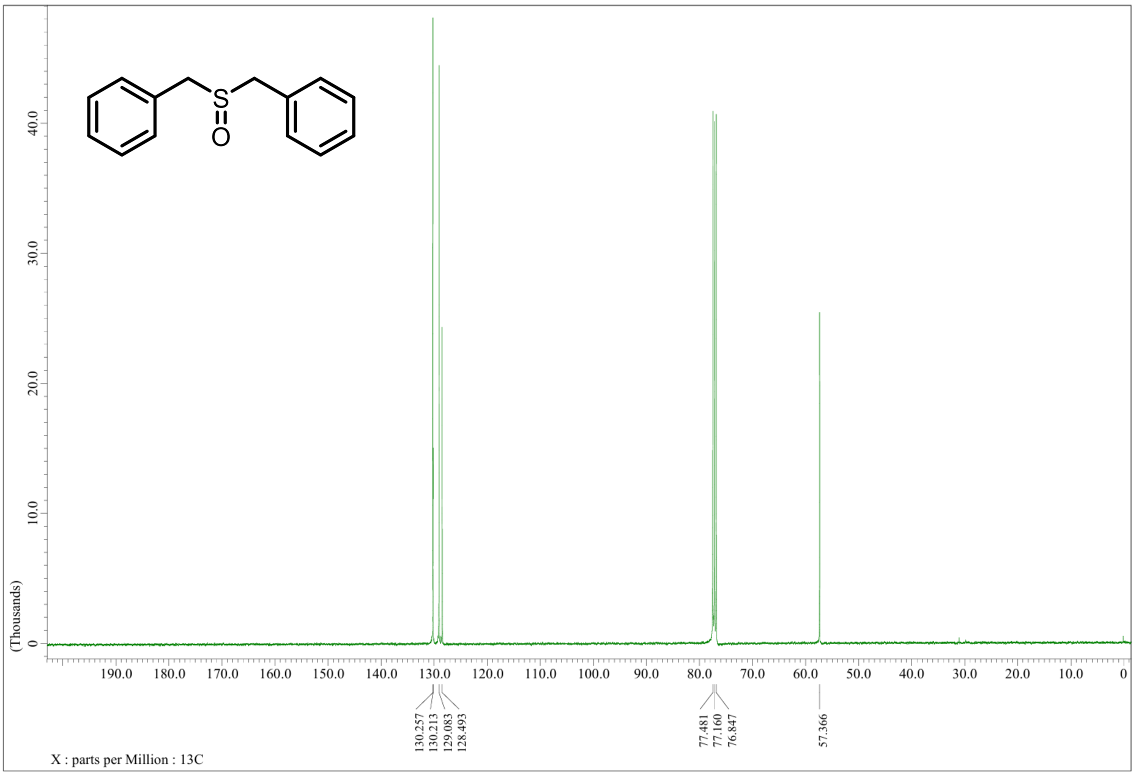

^{1}H NMR (400 MHz, $CDCl_3$, 25 °C) of **2o**

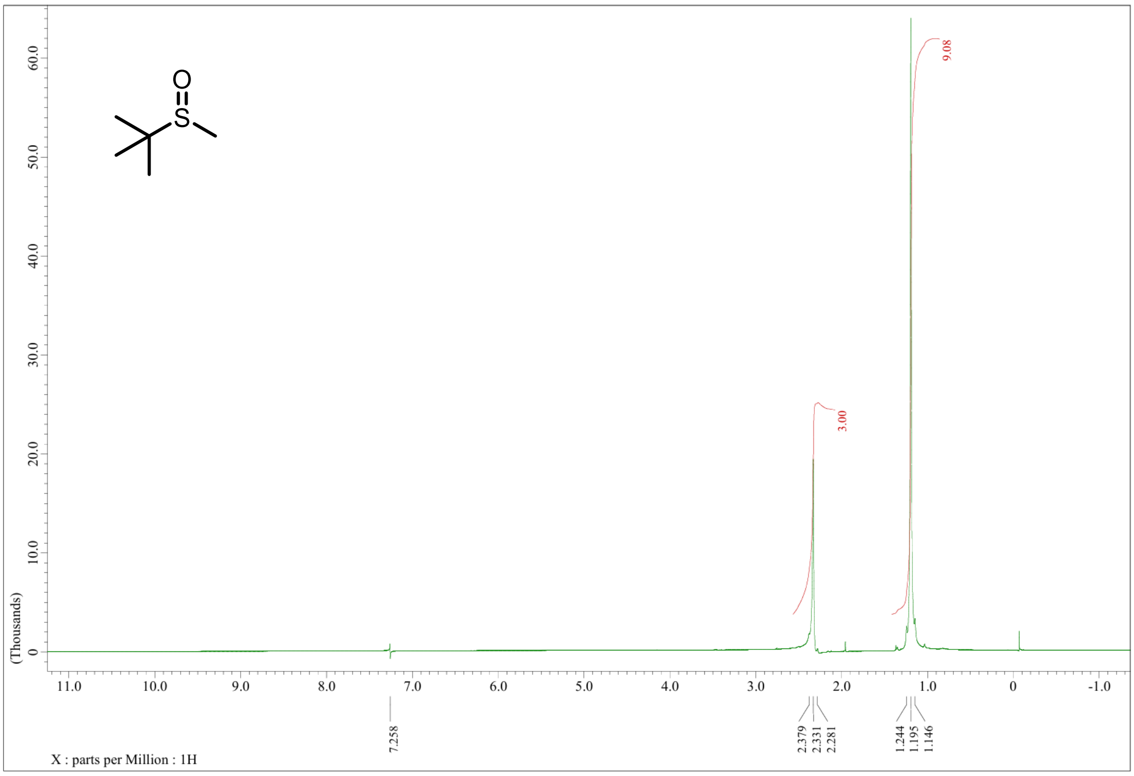

^{13}C NMR (100 MHz, $CDCl_3$, 25 °C) of **2o**

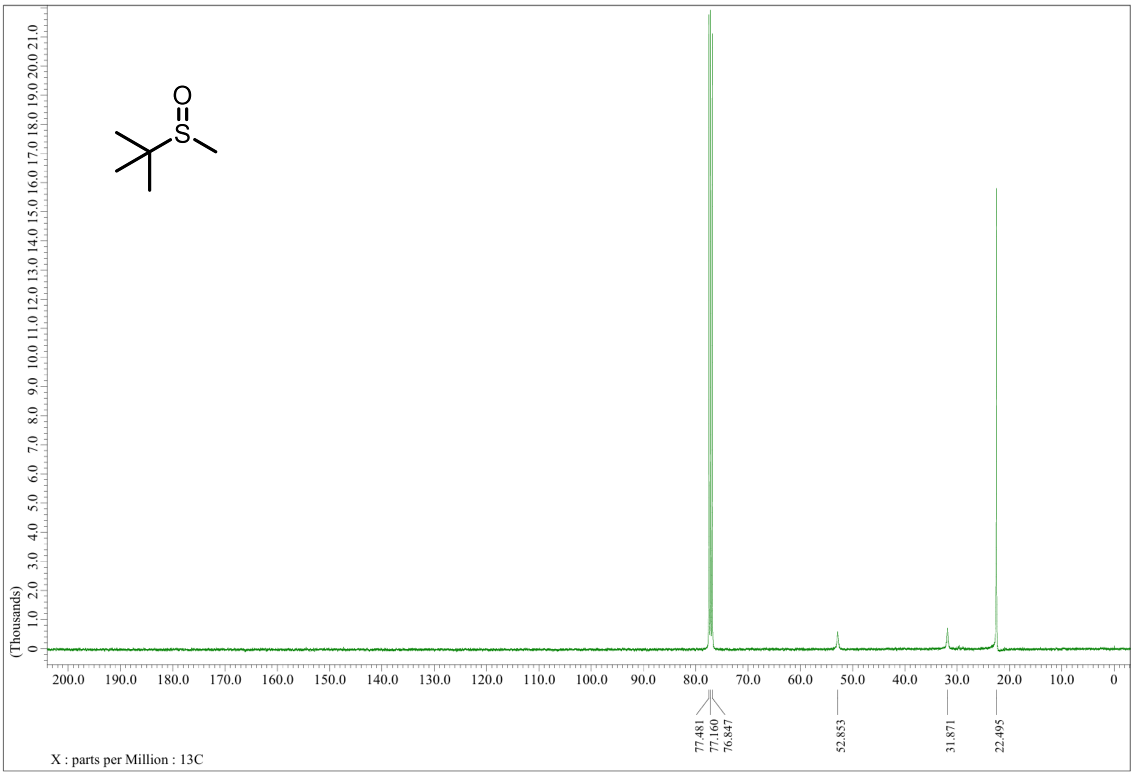

^{1}H NMR (400 MHz, $CDCl_3$, 25 °C) of **2p**

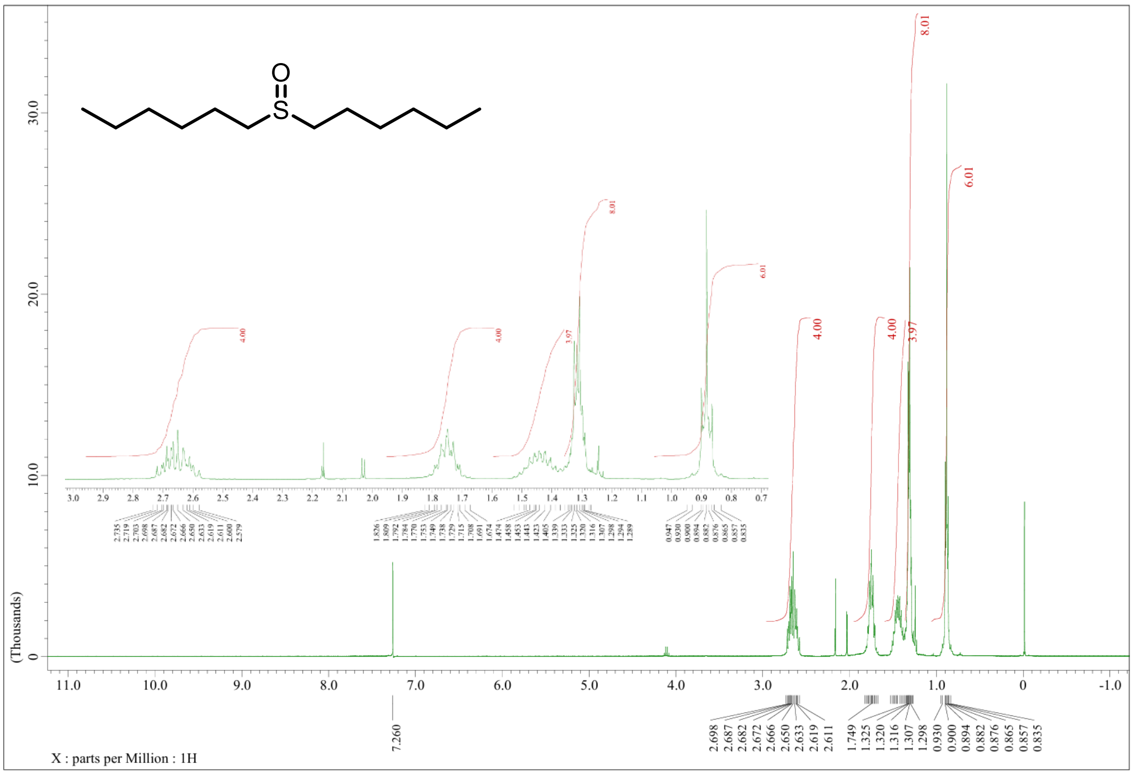

^{13}C NMR (100 MHz, $CDCl_3$, 25 °C) of **2q**

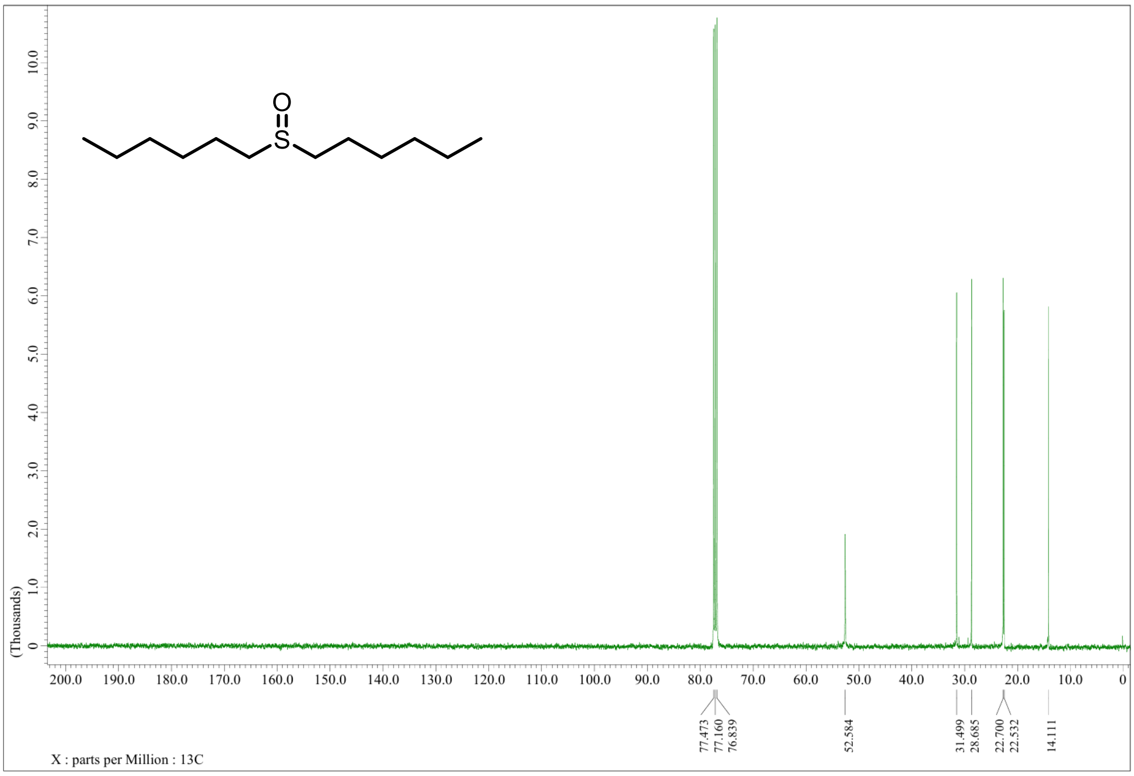

^{1}H NMR (400 MHz, DMSO d$_6$, 25 °C) of **2q**

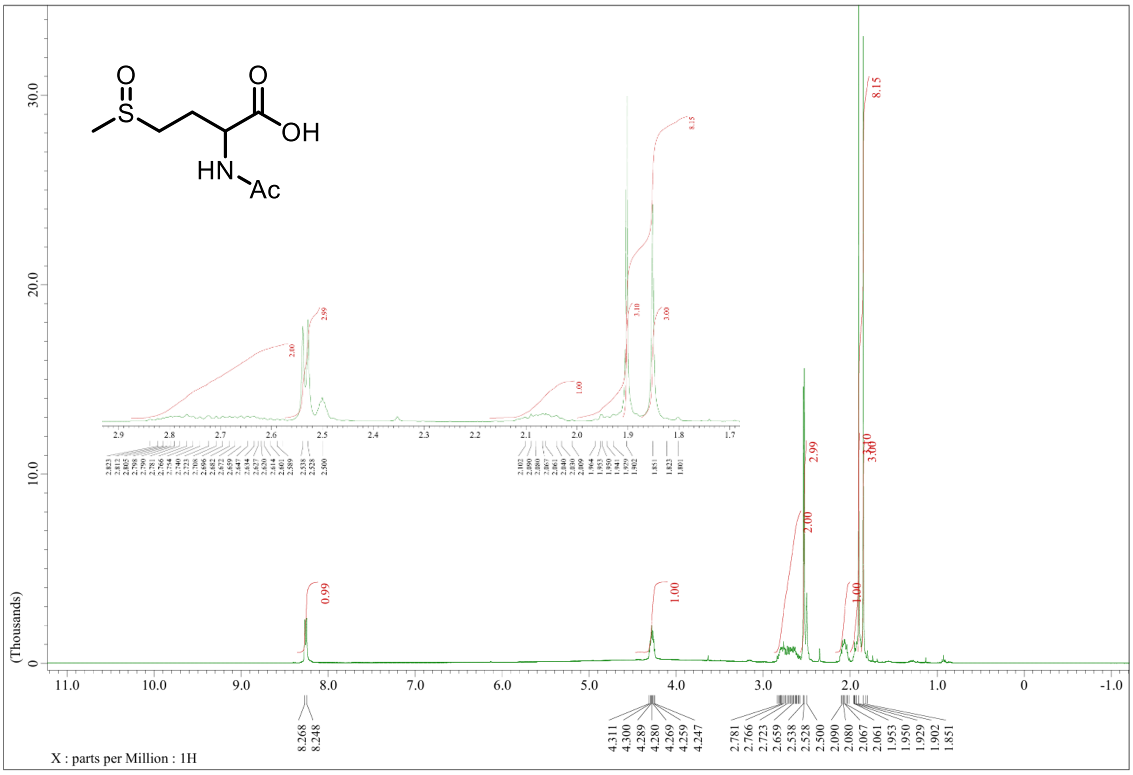

^{13}C NMR (100 MHz, DMSO-d$_6$, 25 °C) of **2r**

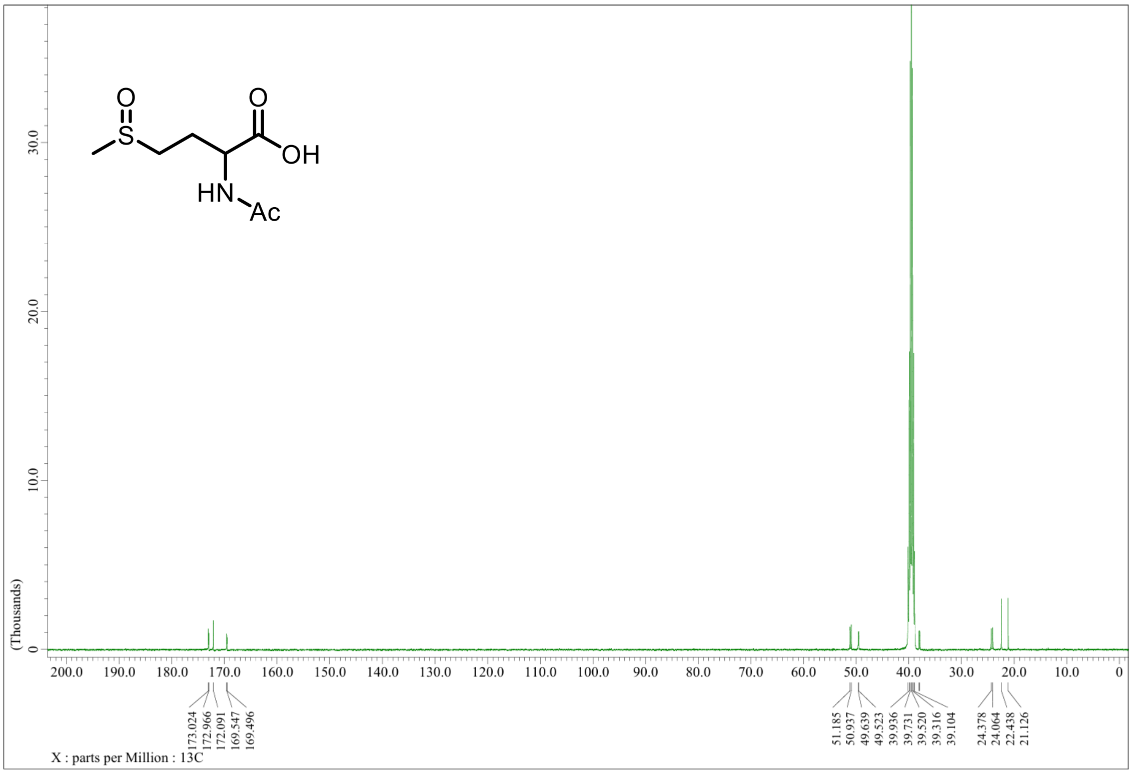

^{1}H NMR (400 MHz, $CDCl_3$, 25 °C) of **2r**

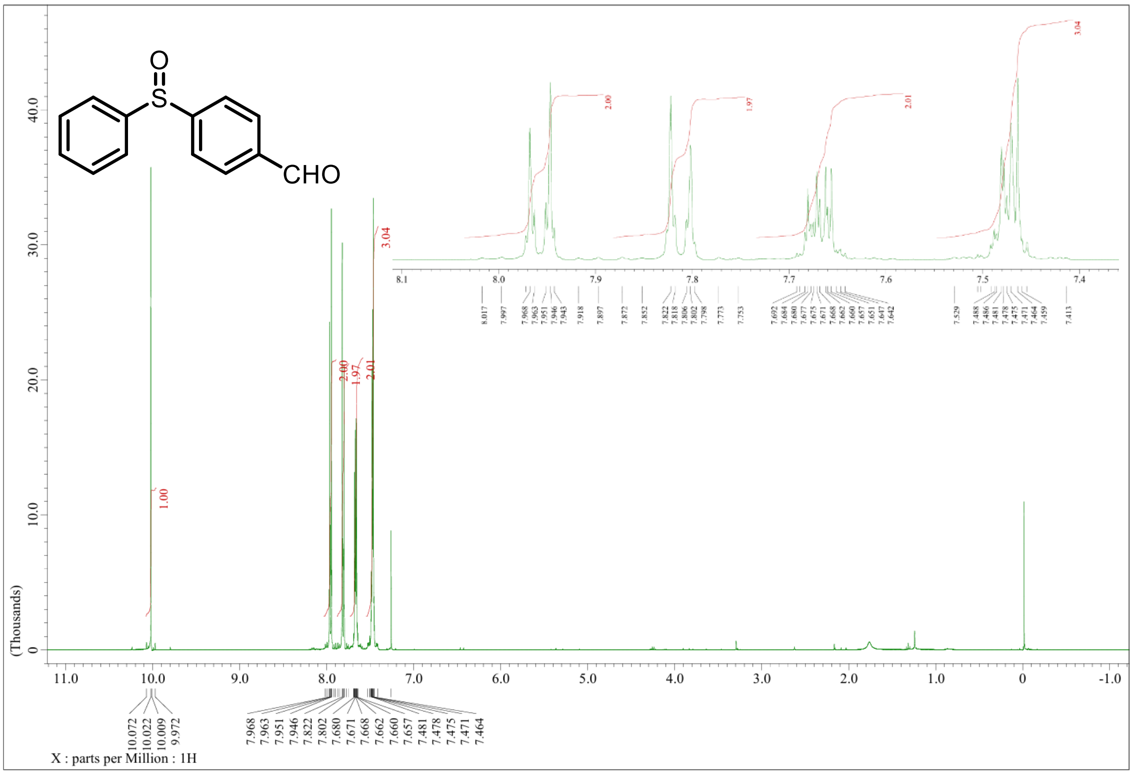

^{13}C NMR (100 MHz, $CDCl_3$, 25 °C) of **2r**

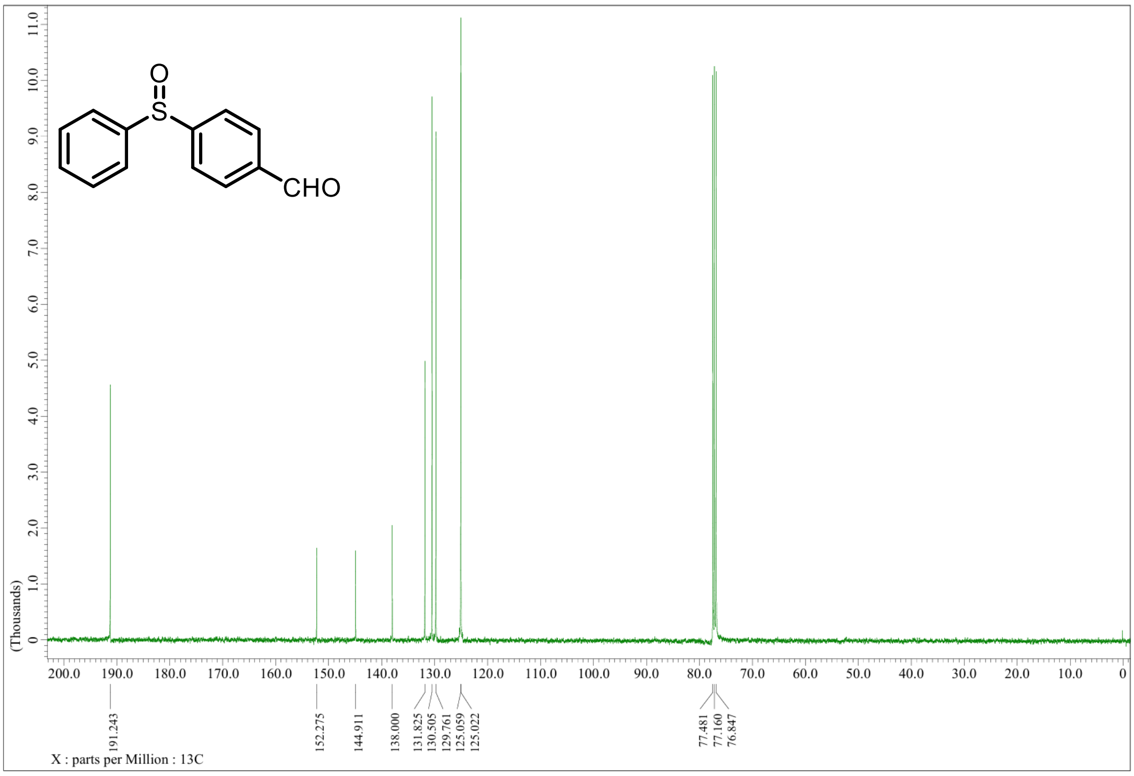

^{1}H NMR (400 MHz, $CDCl_3$, 25 °C) of **2s**

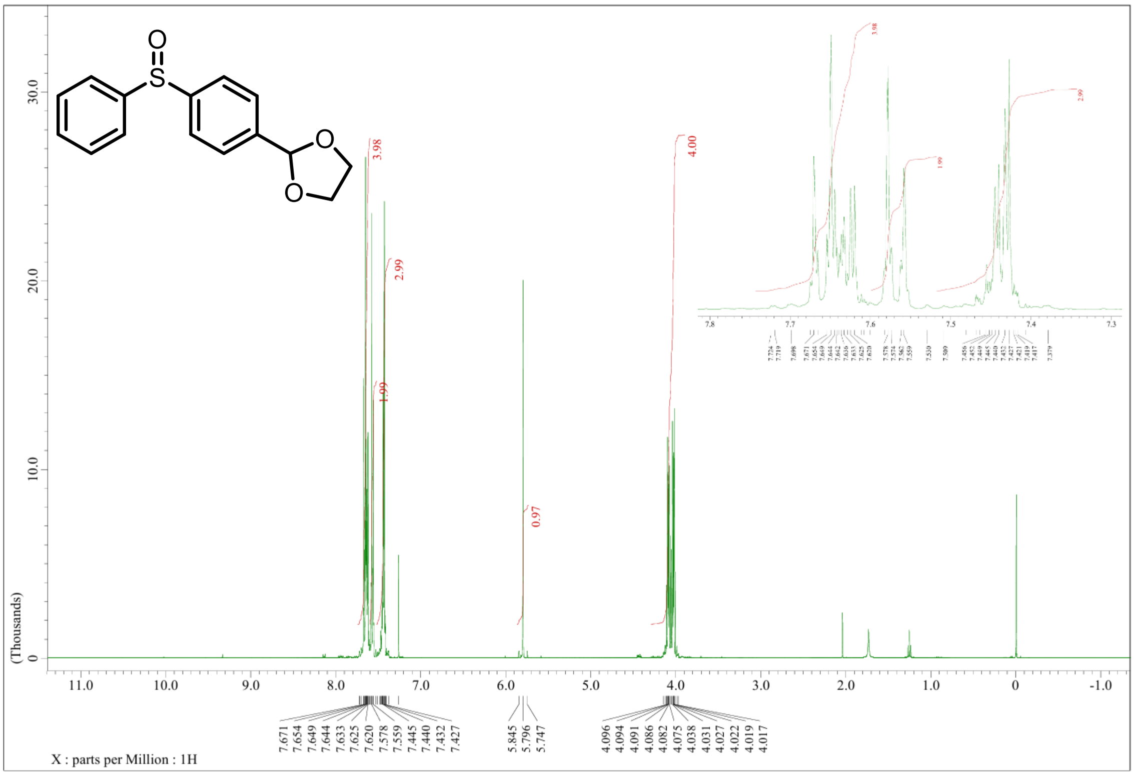

^{13}C NMR (100 MHz, $CDCl_3$, 25 °C) of **2s**

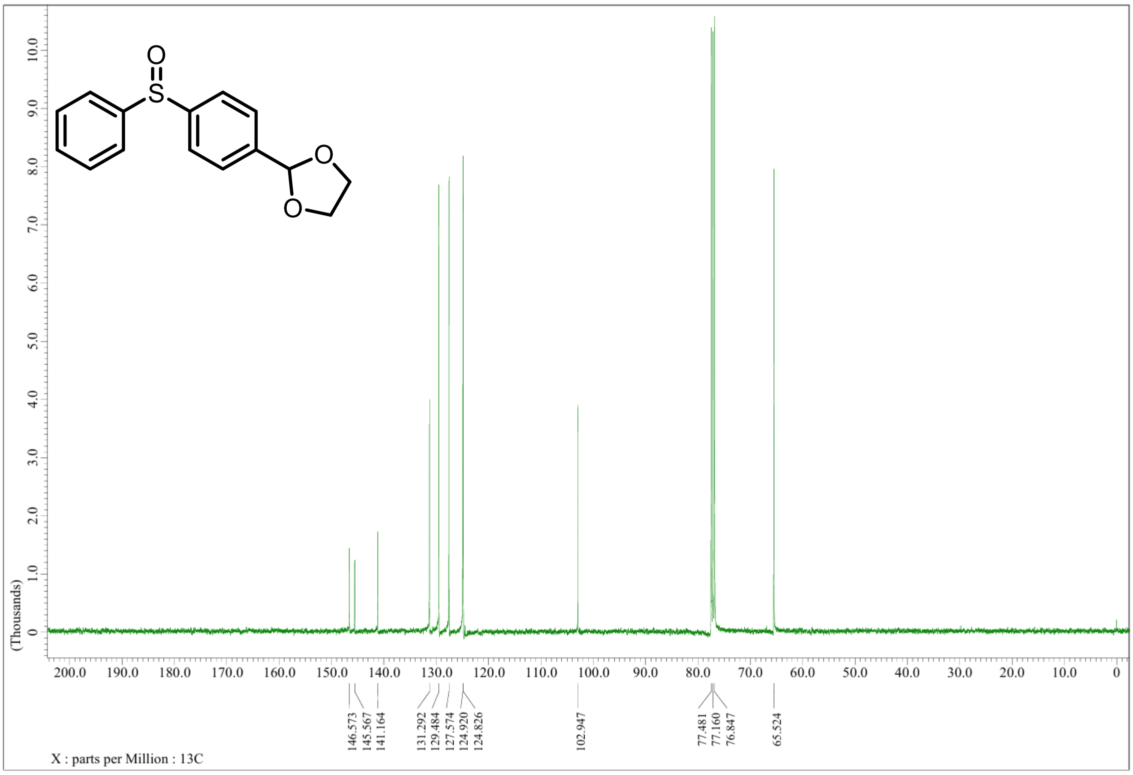

^{1}H NMR (400 MHz, $CDCl_3$, 25 °C) of **3a**

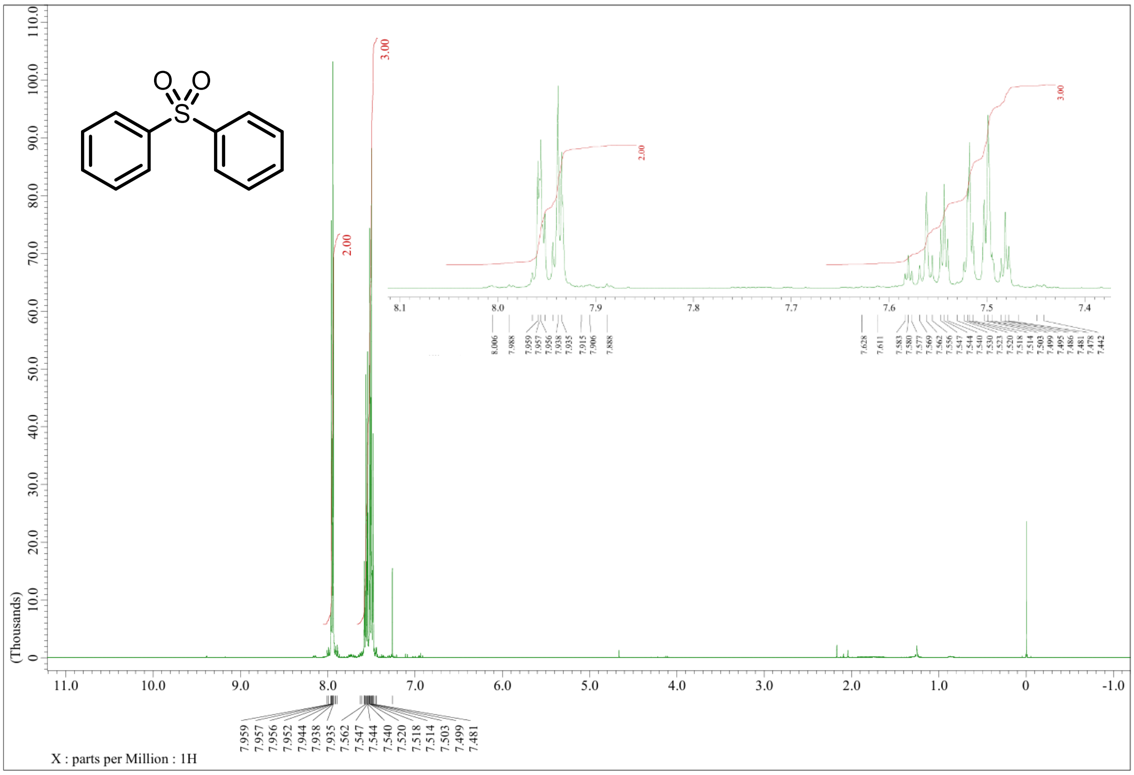

^{13}C NMR (100 MHz, $CDCl_3$, 25 °C) of **3a**

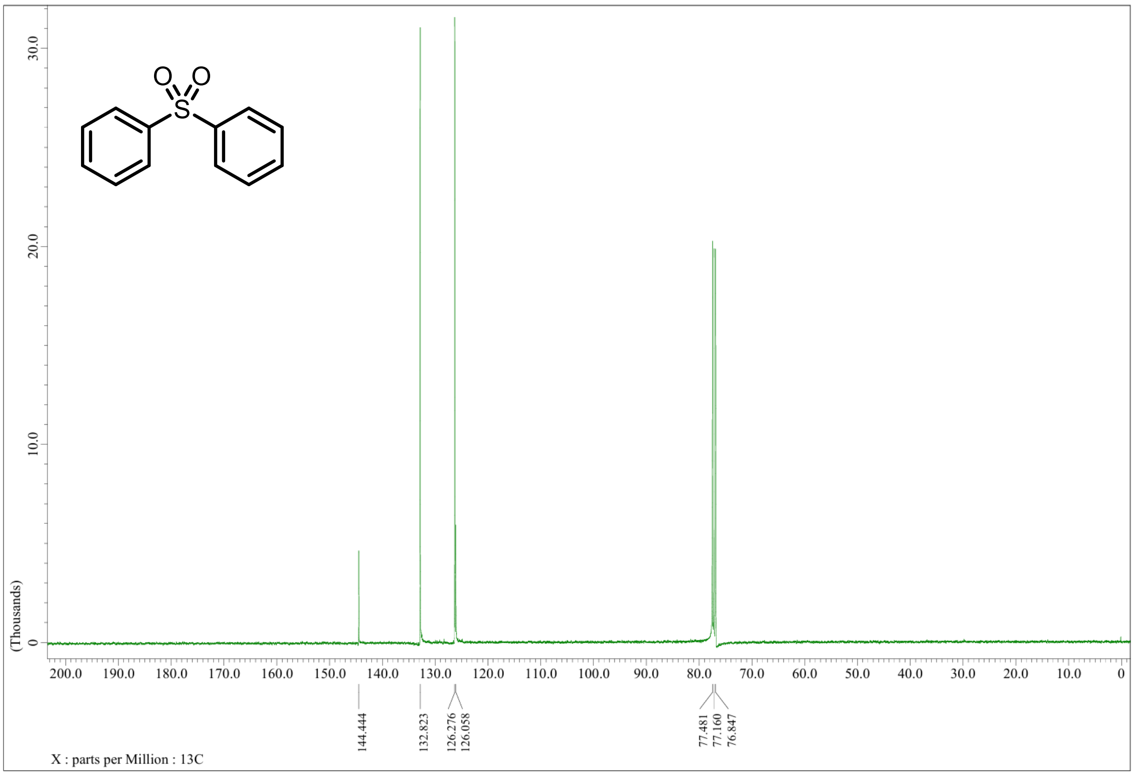

10. References for SI

[1] X. Li, J. Du, Y. Zhang, H. Chang, W. Gao, W. Wei, *Org. Biomol. Chem.* **2019**, *17*, 3048–3055.

[2] R. Noyori, S. Murata, M. Suzuki, *Tetrahedron* **1981**, *37*, 3899–3910.

[3] Gaussian 16, Revision C.02, M. J. Frisch, G. W. Trucks, H. B. Schlegel, G. E. Scuseria, M. A. Robb, J. R. Cheeseman, G. Scalmani, V. Barone, G. A. Petersson, H. Nakatsuji, X. Li, M. Caricato, A. V. Marenich, J. Bloino, B. G. Janesko, R. Gomperts, B. Mennucci, H. P. Hratchian, J. V. Ortiz, A. F. Izmaylov, J. L. Sonnenberg, D. Williams-Young, F. Ding, F. Lipparini, F. Egidi, J. Goings, B. Peng, A. Petrone, T. Henderson, D. Ranasinghe, V. G. Zakrzewski, J. Gao, N. Rega, G. Zheng, W. Liang, M. Hada, M. Ehara, K. Toyota, R. Fukuda, J. Hasegawa, M. Ishida, T. Nakajima, Y. Honda, O. Kitao, H. Nakai, T. Vreven, K. Throssell, J. A. Montgomery, Jr., J. E. Peralta, F. Ogliaro, M. J. Bearpark, J. J. Heyd, E. N. Brothers, K. N. Kudin, V. N. Staroverov, T. A. Keith, R. Kobayashi, J. Normand, K. Raghavachari, A. P. Rendell, J. C. Burant, S. S. Iyengar, J. Tomasi, M. Cossi, J. M. Millam, M. Klene, C. Adamo, R. Cammi, J. W. Ochterski, R. L. Martin, K. Morokuma, O. Farkas, J. B. Foresman, D. J. Fox, Gaussian, Inc. Wallingford CT, **2019**.

[4] H. B. Jeon, K. T. Kim, S. H. Kim, *Tetrahedron Lett.* **2014**, *55*, 3905–3908.

[5] H.-J. Xu, Y.-C. Lin, X. Wan, C.-Y. Yang, Y.-S. Feng, *Tetrahedron* **2010**, 66, 8823–8827.

[6] Z. Cheng, P. Sun, A. Tang, W. Jin, C. Liu, *Org. Lett.* 2019, **21**, 8925–8929.

[7] Q. Fan, L. Zhu, X. Li, H. Ren, G. Wu, H. Zhu, W. Sun, *Green Chem.* **2021**, *23*, 7945–7949.

[8] J. J. Boruah, S. P. Das, S. R. Ankireddy, S. R. Gogoi, N. S. Islam, *Green Chem.* **2013**, *15*, 2944–2959.

[9] I. Tosi, C. Vurchio, M. Abrantes, I. S. Goncalves, M. Pillinger, F. Cavani, F. M. Cordero, A. Brandi, *Catal. Commun.* **2018**, *103*, 60–64.

[10] D. R. Boyd, N. D. Sharma, B. McMurray, S. A. Haughey, C. C. R. Allen, J. T. G. Hamilton, W. C. McRoberts, R. A. M. O'Ferrall, J. N.-Runic, L. A. Coulombel, K. E. O'Connor, *Org. Biomol. Chem.* **2012**, *10*, 782–790.

[11] J. M. Shin, Y. M. Cho, G. Sachs, *J. Am. Chem. Soc.* **2004**, *126*, 7800–7811.

[12] F. Secci, M. Arca, A. Frongia, P. P. Piras, *New J. Chem.* **2014**, *38*, 3622–3629.

[13] M. Zenzola, R. Doran, L. Degennaro, R. Luisi, J. A. Bull, *Angew. Chem. Int. Ed.* **2016**, *55*, 7203–7207.

[14] J. J. Boruah, S. P. Das, S. R. Ankireddy, S. R. Gogoi, N. S. Islam, *Green Chem.* **2013**, *15*, 2944–2959.

[15] T. Neveselý, E. Svobodová, J. Chudoba, M. Sikorski, R. Cibulka, *Adv. Synth. Catal.* **2016**, *358*, 1654–1663.

[16] H. Jiang, T. Jia, M. Zhang, P. J. Walsh, *Org. Lett.* **2016**, *18*, 972–975.

[17] S. K. Aithagani, K. R. Yempalla, G. Munagala, R. A. Vishwakarma, P. P. Singh, *RSC Adv.* **2014**, *4*, 50208.

[18] B. Yu, A.-H. Liu, L.-N. He, B. Li, Z.-F. Diao, Y.-N. Li, *Green chem.* **2012**, *14*, 957–962.
